# DDQ-Promoted Mild and Efficient Metal-Free Oxidative α-Cyanation of *N*-Acyl/Sulfonyl 1,2,3,4-Tetrahydroisoquinolines

**DOI:** 10.3390/molecules23123223

**Published:** 2018-12-06

**Authors:** Hong Pyo Kim, Heesun Yu, Hyoungsu Kim, Seok-Ho Kim, Dongjoo Lee

**Affiliations:** 1 College of Pharmacy and Research Institute of Pharmaceutical Science and Technology (RIPST), Ajou University, 206 Worldcup-ro, Yeongtong-gu, Suwon 16499, Korea; ghim@ajou.ac.kr (H.P.K.); heapang@ajou.ac.kr (H.Y.); hkimajou@ajou.ac.kr (H.K.); 2 Department of Pharmacy, College of Pharmacy and Institute of Pharmaceutical Sciences, CHA University, 120 Haeryong-ro, Gyeonggi-do, Pocheon 11160, Korea

**Keywords:** tetrahydroisoquinoline, oxidation, C(sp^3^)-H activation, α-cyanation

## Abstract

A mild and highly efficient metal-free oxidative α-cyanation of *N*-acyl/sulfonyl 1,2,3,4-tetrahydroisoquinolines (THIQs) has been accomplished at an ambient temperature via DDQ oxidation and subsequent trapping of *N*-acyl/sulfonyl iminium ions with (*n*-Bu)_3_SnCN. Employing readily removable *N*-acyl/sulfonyl groups as protecting groups rather than *N*-aryl ones enables a wide range of applications in natural product synthesis. The synthetic utility of the method was illustrated using a short and efficient formal total synthesis of (±)-calycotomine in three steps.

## 1. Introduction

Direct C(sp^3^)-H functionalization through oxidative coupling is one of the most efficient strategies for the incorporation of functional groups at a proper position [1,2,3,4,5,6] since it does not need the preactivation of a substrate to generate a reactive key intermediate to form a new bond. While this process has been most frequently accomplished through employing transition metal catalysts, significant synthetic endeavors were recently devoted to direct C(sp^3^)-H functionalization under metal-free conditions [7,8,9].

**Figure 1:**
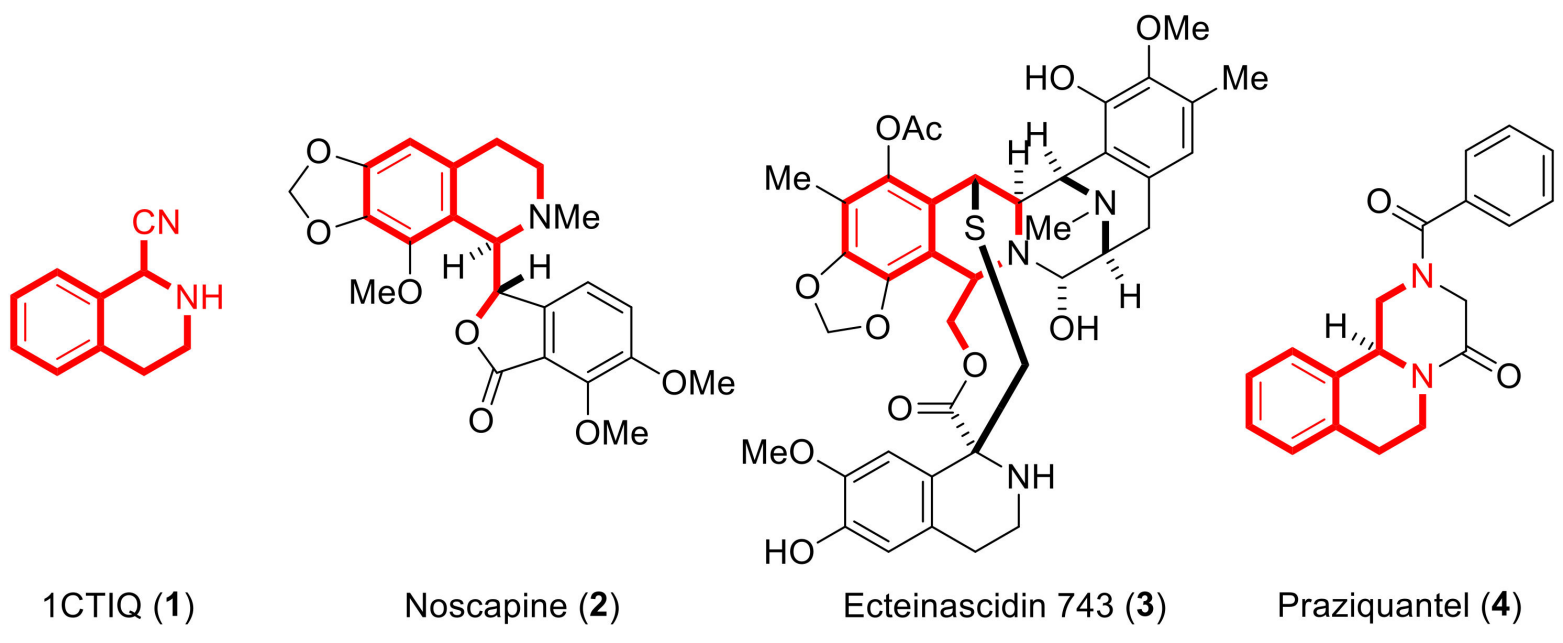
Selected natural products or pharmaceuticals containing α-substituted THIQ moiety.

α-Substituted-1,2,3,4-tetrahydroisoquinoline (THIQ) is a widely distributed structural motif in a wide range of both biologically active natural products and pharmaceutical compounds such as 1CTIQ (**1**, α-cyano-THIQ, monoamine oxidase inhibitor) [10], noscapine (**2**, α-hydroxymethyl THIQ) [11,12], ecteinascidin 743 (**3**, α-hydroxymethyl and α-carboxylic THIQs in the northern and southern parts, respectively, an anticancer agent) [13], and praziquantel (**4**, α-aminomethyl THIQ, an anthelmintic) (Figure 1) [14,15]. In particular, α-cyano THIQ is a highly valuable structural motif and versatile intermediate in that the α-amino nitrile moiety can be easily converted to α-amino carboxylic acid via hydrolysis, along with α-amino aldehydes, ketones by nucleophilic addition, and 1,2-diamines via reduction. Not surprisingly, α-cyano THIQs have attracted considerable attention from synthetic as well as medicinal chemists, which require new and efficient methods for the introduction of a nitrile group at the α-position of THIQs.

**Scheme 1:**
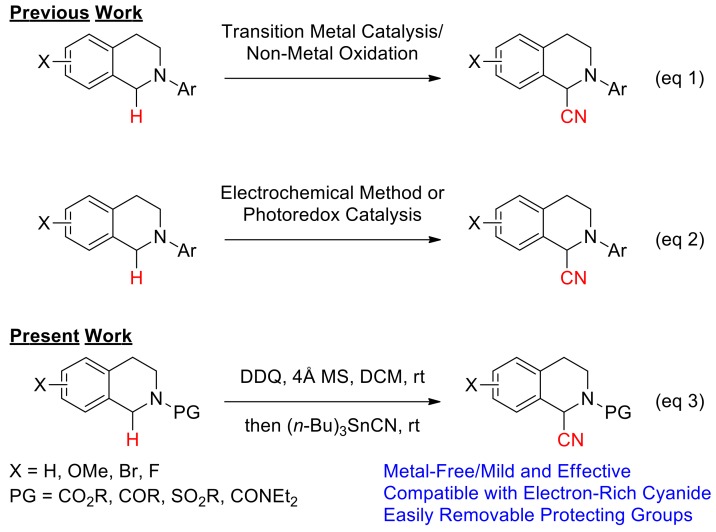
Reported method of oxidative α-cyanation of *N*-protected THIQs.

In recent years, several methods for the direct α-cyanation of *N*-protected THIQs have been developed. Most notable methods involve using a transition metal or metal-free oxidants for the α-cyanation of *N*-aryl THIQs via the direct C(sp^3^)-H functionalization (Scheme 1, Equation (1)) [16,17,18,19,20,21,22,23,24,25]. However, the removal of aryl-protecting groups from the nitrogen in the presence of other functional groups proves to be problematic, which limits the synthetic utility of these approaches. For instance, the removal of a phenyl group from amines required conditions that are only tolerated by a small set of organic compounds (100 equivalent of Li/NH_3_/THF/−40 °C) [26,27,28]. An electrochemical method [16,21] or visible-light photoredox catalysis [18,19,20,23,25] was also developed (Scheme 1, Equation (2)). However, these methods also need specific instrumentation or a catalytic system that is not readily available for general synthetic organic chemistry. Therefore, the development of a new operationally convenient and efficient method for the direct α-cyanation of THIQs bearing an easily removable protecting group instead of an *N*-aryl one would provide an attractive solution for enhancing the scope and utility of α-substituted THIQs, but few examples of such metal-free α-cyanation reactions have been reported to date [29].

Considering that THIQ frameworks are core units within a multitude of biologically active natural products and important pharmaceutical compounds, the development of a practical and efficient method to introduce nitrile group is still a worthwhile project to pursue. Herein, we wish to report our efforts to explore a new mild and efficient method for the direct α-cyanation of THIQs promoted using 2,3-dichloro-5,6-dicyano-1,4-benzoquinone (DDQ) as the oxidant at ambient temperature (Scheme 1, Equation (3)).

## 2. Results and Discussion

### 2.1. Optimization of DDQ-Promoted α-Cyanation of N-Boc THIQ ***5a***

It has been known that the formation of *N*-acyl or *N*-sulfonyl iminium ions is difficult with commonly used oxidants from *N*-acyl or *N*-sulfonyl THIQs, respectively, even in the presence of a transition metal oxidant, thus the selection of the oxidant is important. We selected 2,3-dichloro-5,6-dicyano-1,4-benzoquinone (DDQ) [30,31,32] as an organic oxidizing agent, since it is an inexpensive, stable, readily accessible solid that is easy to handle, and permits more practical as well as mild reaction conditions. To test the viability of the envisioned direct α-cyanation of *N*-acyl/*N*-sulfonyl THIQs at the outset of our studies, *N*-Boc-6,7-dimethoxy THIQ **5a**, which is the most ubiquitous framework in THIQ alkaloids, was selected as a model substrate (Table 1). Treatment of **5a** with DDQ (1.1 equivalent) in the presence of 4 Å molecular sieves (MS) to remove water that might be present in the reaction mixture at room temperature for 30 min, and subsequent addition of a variety of cyanide nucleophiles to trap in situ generated *N*-Boc iminium ion, afforded the desired product (±)-**6a**. Among the cyanide nucleophiles tested, (*n*-Bu)_3_SnCN was found to be the best one (Table 1, entry 4). It is worthwhile to note that a DDQ-mediated direct α-cyanation of *N*-protected THIQs with electron-rich (*n*-Bu)_3_SnCN as the nucleophile has never been attempted, presumably, due to its high propensity for oxidation and loss of nucleophilicity in the presence of oxidizing agents. Trimethylsilyl cyanide (TMSCN) (Table 1, entry 1) also proved to be an effective nucleophile. However, low yield was obtained when *tert*-butyldimethylsilyl cyanide (TBSCN)TBSCN or Zn(CN)_2_ was used as a nucleophile (Table 1, entries 2 and 3). Furthermore, the effects of solvents were also investigated, and the reaction proceeded smoothly in most organic solvents tested including ethyl acetate (EtOAc), toluene, acetone, and tetrahydrofuran (THF) (Table 1, entries 5–8), yet only a moderate yield was obtained when high polarity solvents, such as acetonitrile (MeCN) and *N*,*N*-dimethylformamide (DMF), were used (Table 1, entries 9 and 10). Unlike the result from Wang and co-workers [29], dichlromethane (DCM)DCM, which is non-toxic compared to MeCN, proved to be the best solvent in our experiment.

### 2.2. Reaction Scope with Various N-Protecting Groups of THIQs

**Figure 2:**
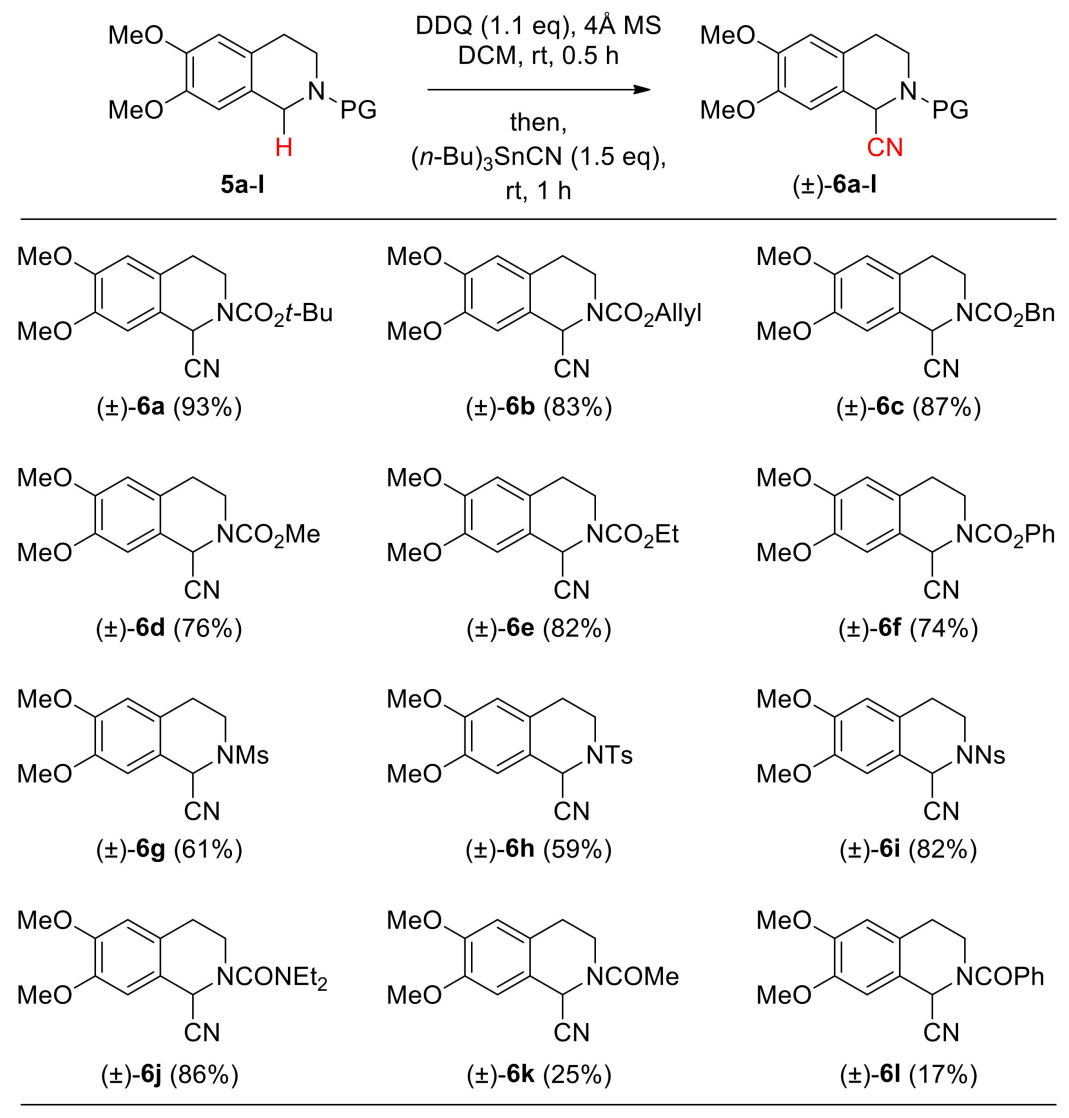
Reaction scope with various *N*-acyl/sulfonyl groups of THIQs ^a,b^. ^a^ Reaction conditions: *N*-Acyl/Sulfonyl-THIQ **5a**-**l** (0.3 mmol, 1 equivalent), DDQ (1.1 equivalent) in DCM (3.0 mL) at room temperature under an argon atmosphere for 0.5 h, then (n-Bu)_3_SnCN (1.5 equivalent) for 1 h. ^b^ Based on the isolated product using chromatography after purification.

With the optimized reaction conditions in hand, we then investigated the scope of *N*-acyl/sulfonyl THIQs of the reaction (Figure 2). The reactions of *t*-butyl carbamate (**5a**), allyl carbamate (**5b**), benzyl carbamate (**5c**), methyl carbamate (**5d**), ethyl carbamate (**5e**), and phenyl carbamate (**5f**) all gave the corresponding products ((±)-**6a**–**f**) with high yields (74–93%). The reactions of 2-nitrophenyl sulfamide (**5g**), 4-tolyl sulfamide (**5h**), and methyl sulfamide (**5i**) also proceeded smoothly to afford the desired products ((±)-**6g**–**i**) with good yields (59–81%). *N*,*N*-Diethyl carboxamide **5j** proved to be an effective substrate to afford the desired product (±)-**6j** with 86% yield. To our surprise, however, both acetamide (**5k**) and benzamide (**5l**) gave the corresponding products ((±)-**6k**) and ((±)-**6l**) with poor yields (25% and 17%, respectively) under the optimized conditions. This result indicates that the *N*-acyliminium intermediates in situ generated from the amide substrates were less stable or less electrophilic than those derived from carbamate or sulfamide ones.

### 2.3. Reaction Scope with Electronically Diverse N-Boc THIQs

**Figure 3:**
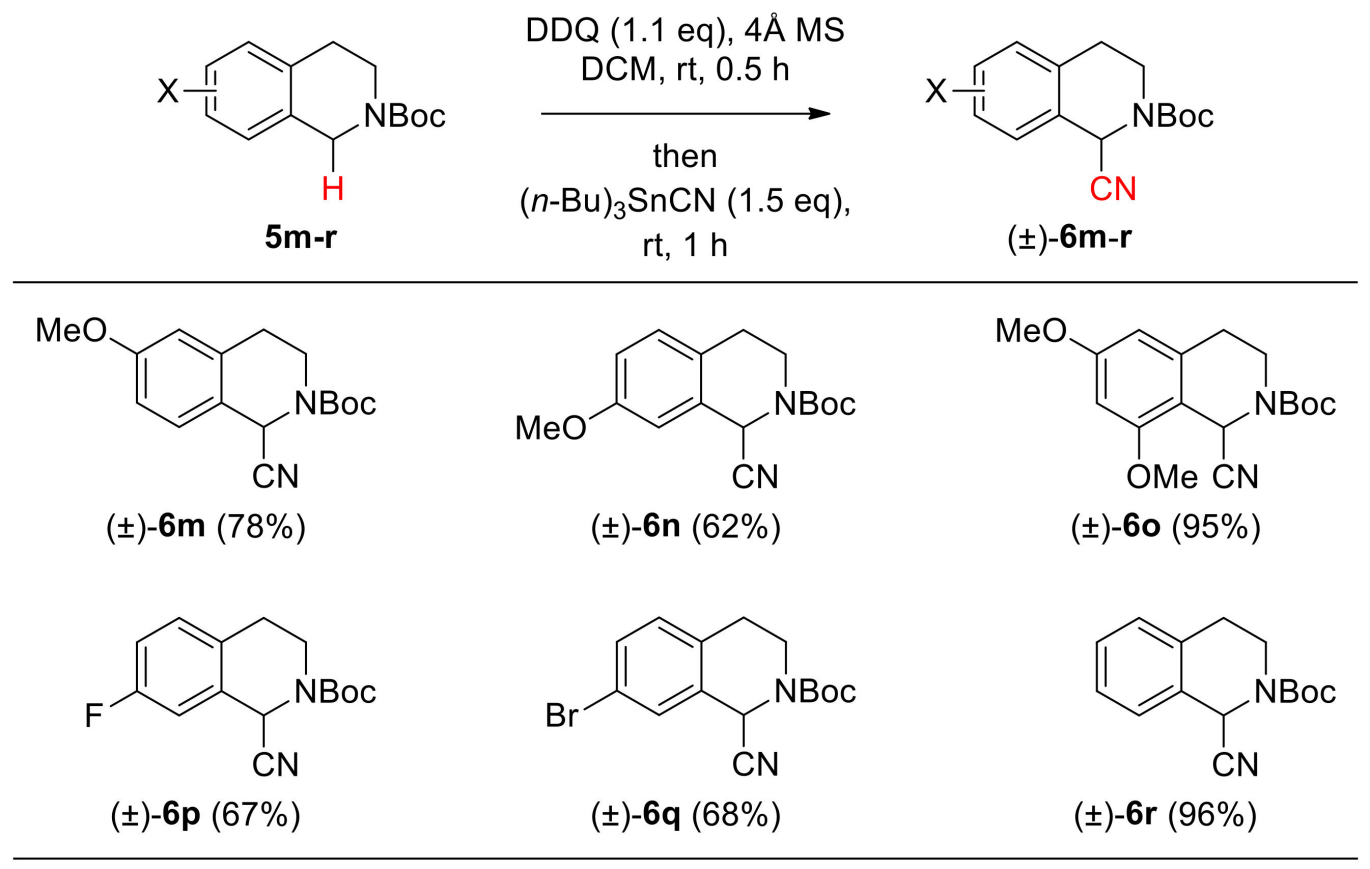
Reaction scope with electronically diverse *N*-Boc THIQs ^a,b^. ^a^ Reaction conditions: *N*-Boc-THIQ **5m**-**r** (0.3 mmol, 1 equivalent), DDQ (1.1 equivalent) in DCM (3.0 mL) at room temperature under argon atmosphere for 0.5 h, then (n-Bu)_3_SnCN (1.5 equivalent) for 1 h. ^b^ Based on the isolated product using chromatography after purification.

We also investigated the broad scope with respect to electronically diverse *N*-Boc THIQs (Figure 3). As expected, direct α-cyanation of *N*-Boc THIQs with electron-donating substituents (**5a**–**c**) proceeded smoothly to afford the corresponding products ((±)-**6a**–**c**) with good to excellent yield (62–95%). Notably, *N*-Boc THIQs bearing electron-withdrawing substituents, such as fluorine (**5p**) and bromine (**5q**), were tolerated to afford the desired products ((±)-**6p**) and ((±)-**6q**) with good yields (67% and 68%, respectively) for further diversifications. Also, *N*-Boc THIQ (**5r**) with hydrogen substituents proved to be a good substrate to afford the corresponding product ((±)-**6r**) with a 96% yield.

**Scheme 2:**
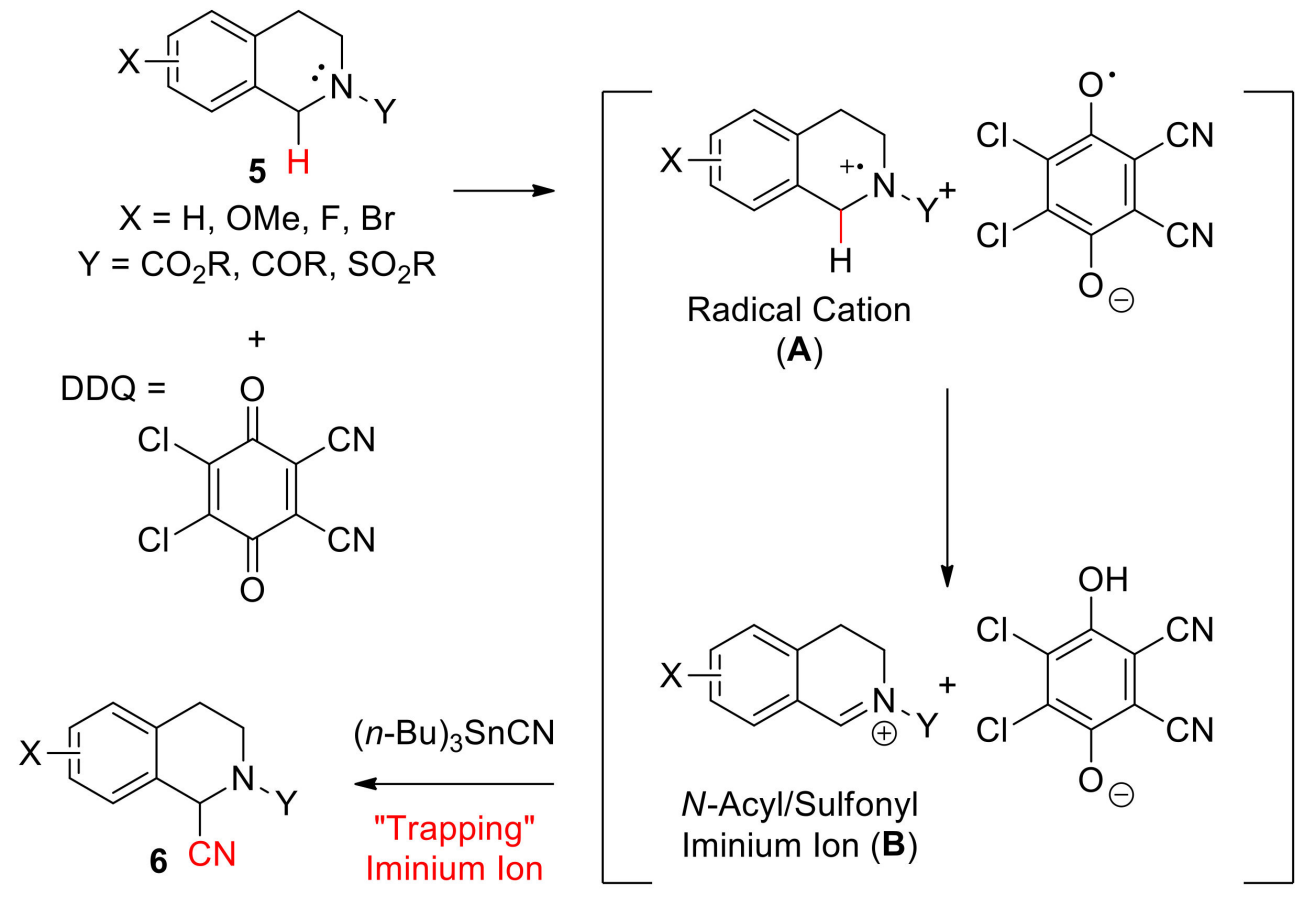
Plausible mechanism for DDQ-promoted a-cyanation of *N*-acyl/sulfonyl THIQ.

A plausible mechanism for the DDQ-promoted oxidative α-cyanation of *N*-acyl/sulfonyl THIQs was proposed in Scheme 2. *N*-acyl/sulfonyl THIQ (**5**) was oxidized to generate a radical cation **A** by a single electron transfer from *N*-acyl/sulfonyl THIQ to DDQ [33]. Then, the DDQ radical oxygen abstracted a H-atom from (**A**) to generate a stable and reactive iminium ion (**B**). Finally, the trapping the iminium ion (**B**) with (*n*-Bu)_3_SnCN afforded the desired *N*-acyl/sulfonyl α-cyanated THIQ (**6**).

**Scheme 3:**
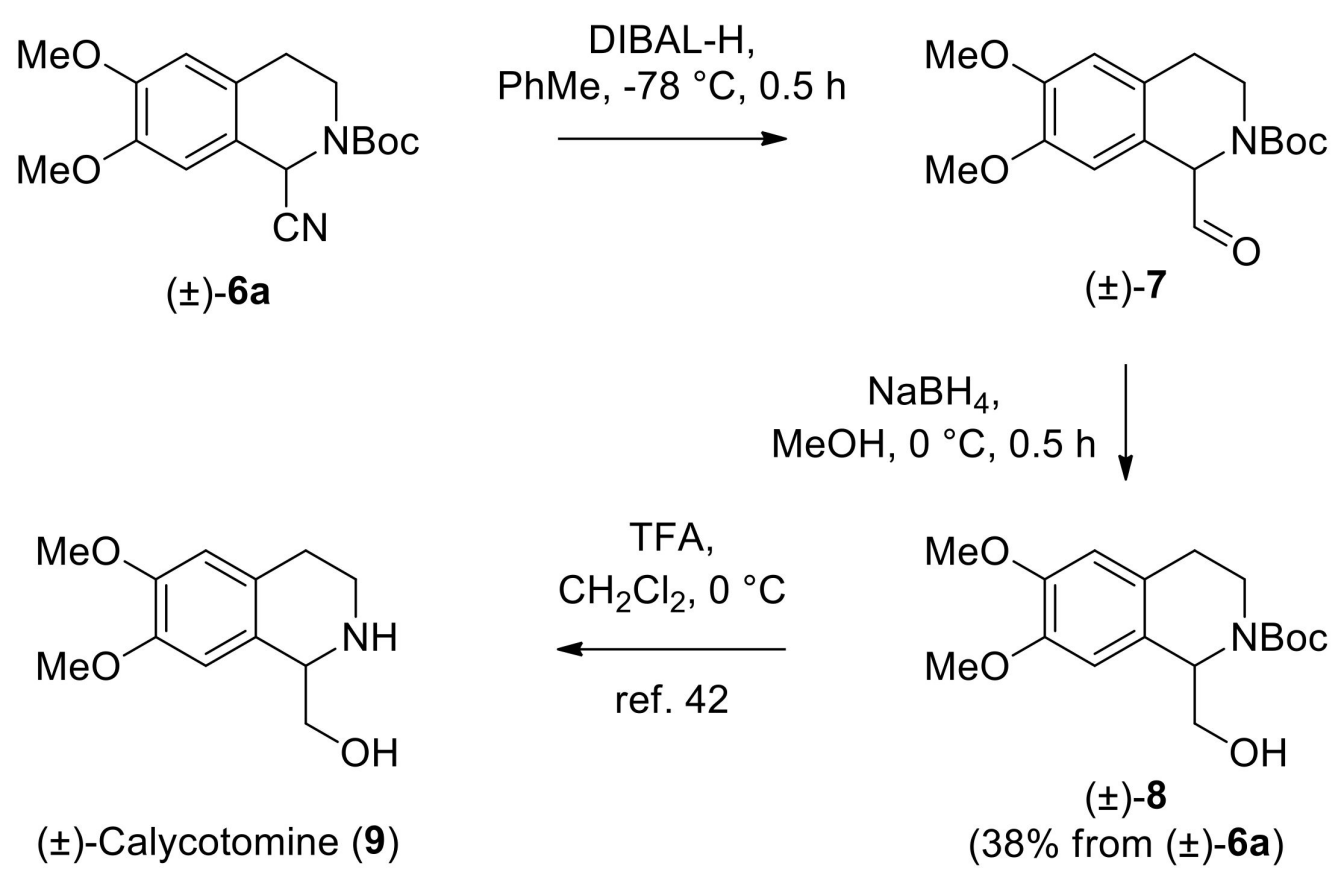
A short and efficient formal total synthesis of (±)-calycotomine (**9**).

We next turned on our attention to a short and efficient formal total synthesis of (±)-calycotomine (**9**) to prove the synthetic utility of this method (Scheme 3). Calycotomine (**9**) is hydroxymethyl THIQ alkaloid and was isolated from many plants including *Calycotome spinosa* Link, Leguminosae, *Cystius proliferus*, *Acacia concinna*, and mainly genus *Genista* [34,35,36,37,38]. This natural product was found to exhibit an antimicrobial activity with minimum inhibitory concentration (MIC) MIC 2–8 mg/mL against *Enterobacteriaceae* and *Pseudomonas aeruginosa*. [38] Not surprisingly, calycotomine (**9**) and its analogues have attracted considerable attention from synthetic and medicinal communities due to its interesting pharmacological activities [35,39,40]. Nucleophilic addition to nitrile of (±)-**6a** with DIBAL-H at −78 °C afforded the resulting aldehyde (±)-**7**, which was directly transformed into the corresponding (±)-*N*-Boc calycotomine (**8**) with a 38% yield over two steps through a subsequent reduction with NaBH_4_ due to its instability. The spectral characteristics of our synthetic material (±)-**8** were in good agreement with those reported for synthetic (±)-*N*-Boc calycotomine (**8**) by Jung and co-workers [40].

## 3. Materials and Methods

### 3.1. General Information

**General Methods**: Except as otherwise noted, reactions were carried out under an argon (Ar) or nitrogen (N_2_) atmosphere in flame- or oven-dried glassware. In aqueous work-up, all organic solutions were separated from the aqueous layer using a separatory funnel and combined organic layers were dried over Na_2_SO_4_ or MgSO_4_, and filtered prior to rotary evaporation at diaphragm pump pressure. Reactions were monitored using TLC (thin layer chromatography) with 0.25 mm E. Merck pre-coated silica gel plates (Kieselgel 60F_254_, Merck, Kenilworth, NJ, USA). Spots were detected by viewing under a UV light, colorizing with charring after dipping in *p*-anisaldehyde staining solution with acetic acid and sulfuric acid and MeOH, or in KMnO_4_ solution with sulfuric acid and ethanol, or ceric ammonium molybdate solution with sulfuric acid and ethanol. We used silica gel of particle size 0.040–0.063 mm (Merck, Kenilworthm, NJ, USA) for flash chromatography. Yields were calculated according to chromatographically and spectroscopically pure compounds unless otherwise indicated.

**Materials**: Commercial reagents and solvents were used without further purification with the following exceptions. All solvents were freshly distilled and dried by standard techniques just before use. Tetrahydrofuran (THF) was distilled from sodium/benzophenone ketyl. Acetonitrile (MeCN), dichloromethane (CH_2_Cl_2_), and toluene (PhMe) were distilled from calcium hydride (CaH_2_). Acetone, ethyl acetate (EtOAc), and *N*,*N*-dimethylformamide (DMF) were distilled from magnesium sulfate (MgSO_4_).

**Instrumentation**: ^1^H and ^13^C spectra were recorded on a Mercury-400BB (Varian, Palo Alto, CA, USA) or JNM-ECZ 600R (JEOL, Tokyo, Japan). Chemical shifts of the compound are reported as δ value relative to CHCl_3_ (δ 7.26 for ^1^H-NMR and δ 77.0 for ^13^C-NMR). Data are represented as follows: chemical shift, multiplicity (s = singlet, d = doublet, t = triplet, q = quartet, m = multiplet, br = broad), integration, coupling constant in Hz, and assignment. Infrared (IR) spectra were measured on a 1600 FT-IR spectrometer (Perkin-Elmer, Waltham, MA, USA) referenced to a polystyrene standard. Data are represented as follows: frequency (cm^−1^), intensity (s = strong, m = medium, w = weak, br = broad), and assignment (where appropriate). High resolution mass spectra were recorded at the center for research facilities of Kyunghee University using JMS-700 (FAB+ or EI+, JEOL, Tokyo, Japan). High resolution values were calculated to four decimal places from the molecular formula, all found values being within a tolerance of 5 ppm. Melting point (m.p.) was obtained using IA9100 (Thermo, Waltham, MA, USA).

### 3.2. Experimental Part Method

#### 3.2.1. General Procedure for the Synthesis of *N*-Protected 1,2,3,4-Tetrahydroisoquinolines

To a stirred solution of 1,2,3,4-THIQ (1.0 equiv.) in CH_2_Cl_2_ (10.0 mL/mmol), triethylamine (1.2 equivalent) was added and then cooled to 0 °C. Acyl chloride (1.2 equivalent), sulfonyl chloride (1.2 equivalent), or diethylcarbamoyl chloride (1.2 equivalent) was added slowly at 0 °C. The resulting reaction mixture was stirred at room temperature for 2 h under an argon atmosphere and then poured onto water (10.0 mL/mmol) and the organic layer was separated. The aqueous layer was extracted two times with CH_2_Cl_2_ (10.0 mL/mmol), and the combined organic layer was washed with brine (5.0 mL/mmol), dried over sodium sulfate, filtered, and concentrated under reduced pressure. Purification of the crude residue by flash column chromatography on silica gel, using the appropriate mixture of eluents, provided the corresponding *N*-protected 1,2,3,4-tetrahydroisoquinoline. Spectral data (^1^H- and ^13^C-NMR) of compounds (**5a**, **5c**, **5d**, **5e**, **5g**, **5h**, **5k**, **5l**, **5m**, **5n**, **5o**, **5q**, **5r**) which were reported previously were compared and found in agreement with literature data. Furthermore, references were represented in supporting information. The characterization of novel compounds is given.

*Allyl 6,7-dimethoxy-3,4-dihydroisoquinoline-2(1H)-carboxylate* (**5b**) Yield 88%, colorless oil; ^1^H-NMR (400 MHz, CDCl_3_) δ 6.61 (s, 1H), 6.58 (s, 1H), 5.97 (ddd, *J* = 16.4, 11.2, 5.6 Hz, 1H), 5.32 (d, *J* = 16.4 Hz, 1H), 5.22 (d, *J* = 11.2 Hz, 1H), 4.64 (d, *J* = 5.6 Hz, 2H), 4.57 (s, 2H), 3.854 (s, 3H), 3.849 (s, 3H), 3.70 (t, *J* = 5.6 Hz, 2H), 2.78 (t, *J* = 5.6 Hz, 2H); ^13^C-NMR (100 MHz, CDCl_3_) δ 154.8, 147.3, 132.8, 126.0, 124.9, 124.4, 117.1, 111.3, 108.8, 65.8, 55.8, 45.2, 41.4, 28.2; FT-IR (thin film, neat) ν_max_ 3059, 2935, 2837, 2306, 1543, 1517, 1463, 1371, 1351, 1257, 1226, 1163, 1116 cm^−1^; HRMS (FAB+) found 278.1389 [calculated for C_15_H_20_NO_4_ ([M + H]^+^): 278.1392].

*Phenyl 6,7-dimethoxy-3,4-dihydroisoquinoline-2(1H)-carboxylate* (**5f**) Yield 91%, white solid; m.p. 103–104 °C; ^1^H-NMR (600 MHz, CDCl_3_, at room temperature, 1:1 ratio amide bond) δ 7.36 (t, *J* = 7.8 Hz, 2H), 7.2 (t, *J* = 7.8 Hz, 1H), 7.13 (d, *J* = 7.8 Hz, 2H), 6.66 (s, 1H), 6.63 (s, 1H), 4.77 (s, 1H), 4.64 (s, 1H), 3.88 (s, 3H), 3.86 (s, 4H), 3.79 (t, *J* = 5.4 Hz, 1H), 2.87 (d, *J* = 7.2 Hz, 1H), 2.86 (d, *J* = 7.2 Hz, 1H); ^13^C-NMR (150 MHz, CDCl_3_, rotameric mixture, resonances for minor rotamer are enclosed in parenthesis) δ 153.5, (153.4), 151.1, 147.5, 128.9, 126.0, (125.7), 124.9, 124.7, (124.2), 121.4, 111.4, (111.2), 108.9, (108.8), 55.6, 45.5, (45.4), (42.0), 41.4, (28.2), 28.0; FT-IR (thin film, neat) ν_max_ 2935, 2836, 1719, 1518, 1425, 1202, 1116, 752 cm^−1^; HRMS (FAB+) found 314.1393 [calculated for C_18_H_20_NO_4_ ([M + H]^+^): 314.1392].

6,7-*dimethoxy-2-((2-nitrophenyl)sulfonyl)-1,2,3,4-tetrahydronaphthalene* (**5i**) Yield 82%, colorless oil; ^1^H-NMR (400 MHz, CDCl_3_) δ 6.62 (s, 1H), 6.58 (s, 1H), 4.32 (s, 2H), 3.86 (s, 6H), 3.45 (t, *J* = 6.0 Hz, 2H), 3.25 (t, *J* = 7.2 Hz, 4H), 2.83 (t, *J* = 6.0 Hz, 2H), 1.15 (t, *J* = 7.2 Hz, 6H); ^13^C-NMR (100 MHz, CDCl_3_) δ 148.1, 147.7, 147.6, 133.6, 131.5, 130.6, 124.9, 124.0, 123.1, 111.4, 108.7, 56.0, 55.9, 46.9, 43.8, 28.5; FT-IR (thin film, neat) ν_max_ 2995, 2937, 2835, 2358, 2341, 1697, 1517, 1433, 1257, 1224, 1095 cm^−1^; HRMS (FAB+) found 378.1012 [calculated for C_17_H_18_N_2_O_6_S ([M]^+^): 378.1011].

*N*,*N*-*Diethyl-6,7-dimethoxy-3,4-dihydroisoquinoline-2(1H)-carboxamide* (**5j**) Yield 99%, colorless oil; ^1^H-NMR (600 MHz, CDCl_3_) δ 6.62 (s, 1H), 6.58 (s, 1H), 4.32 (s, 2H), 3.86 (s, 6H), 3.45 (t, *J* = 6.0 Hz, 2H), 3.25 (t, *J* = 7.2 Hz, 4H), 2.83 (t, *J* = 6.0 Hz, 2H), 1.15 (t, *J* = 7.2 Hz, 6H); ^13^C-NMR (150 MHz, CDCl_3_) δ 164.1, 147.3, 147.2, 126.1, 125.6, 111.3, 108.9, 55.6, 55.5, 48.2, 44.8, 41.5, 27.9, 12.9; FT-IR (thin film, neat) ν_max_ 2968, 2933, 1640, 1518, 1419, 1257, 1228, 1117 cm^−1^; HRMS (FAB+) found 293.1869 [calculated for C_16_H_25_N_2_O_3_ ([M + H]^+^): 293.1865].

*tert*-*Butyl 7-fluoro-3,4-dihydroisoquinoline-2(1H)-carboxylate* (**5p**) Yield 67%, colorless oil; ^1^H-NMR (400 MHz, CDCl_3_) δ 7.07 (dd, *J* = 8.0, 5.6 Hz, 1H), 6.86 (td, *J* = 8.4, 2.8 Hz, 1H), 6.81 (d, *J* = 9.2 Hz, 1H), 4.54 (s, 2H), 3.63 (s, 2H), 2.79 (t, *J* = 5.6 Hz, 2H), 1.49 (s, 9H); ^13^C-NMR (100 MHz, CDCl_3_, rotameric mixture, resonances for minor rotamer are enclosed in parenthesis) δ 162.4, 159.9, 154.7, 130.1, (113.6), 113.4, 112.7, 80.0, 46.0, (45.4), (42.1), 40.9, 28.7, 28.5; FT-IR (thin film, neat) ν_max_ 2973, 1752, 1692, 1514, 1387, 1271, 1205, 1175, 765 cm^−1^; HRMS (EI+) found 251.1318 [calculated for C_14_H_18_FNO_2_ ([M]^+^): 251.1322].

#### 3.2.2. General Procedure for the Synthesis of *N*-protected-1-cyano-1,2,3,4-tetrahydroisoquinolines

To a stirred solution of *N*-protected-1,2,3,4-THIQ (0.30 mmol) in CH_2_Cl_2_ (3.0 mL), 4 Å MS (molecular sieves, 120 mg) was added at room temperature. After the reaction mixture was stirred for 15 min at room temperature, 2,3-dichloro-5,6-dicyano-1,4-benzoquinone (DDQ) (0.45 mmol, 1.1 equivalent) was added portionwise. Then the reaction mixture was stirred at room temperature for another 30 min under an argon atmosphere. Tributyltin cyanide ((*n*-Bu)_3_SnCN) (0.75 mmol, 2.5 equivalent) was added dropwise at room temperature and the reaction mixture was stirred at room temperature for 1 h under argon atmosphere, then quenched with saturated aqueous NaHCO_3_ solution (5 mL) and the layers were separated. The aqueous layer was extracted two times with CH_2_Cl_2_ (20 mL), and the combined organic layer was washed with brine (5 mL), dried over sodium sulfate, filtered, and concentrated in vacuo. Purification of the residue using flash column chromatography on silica gel, using hexanes/EtOAc as an eluent, provided the corresponding *N*-protected-1-cyano-1,2,3,4-tetrahydroisoquinoline.

(±)-*tert*-*Butyl 1-cyano-6,7-dimethoxy-3,4-dihydroisoquinoline-2(1H)-carboxylate* (**6a**) Yield 93%, colorless oil; ^1^H-NMR (400 MHz, CDCl_3_, at room temperature, 1.2:1 ratio amide bond) δ 6.75 (s, 1H), 6.64 (s, 1H), 6.01 (brs, 0.5H), 5.79 (brs, 0.5H), 4.29 (brs, 0.5H), 4.12 (brs, 0.5H), 3.89 (s, 3H), 3.87 (s, 3H), 3.36 (brs, 0.5H), 3.23 (brs, 0.5H), 2.84–2.92 (m, 1H), 2.71–2.75 (m, 1H), 1.53 (s, 9H); ^13^C-NMR (100 MHz, CDCl_3_, rotameric mixture, resonances for minor rotamer are enclosed in parenthesis) δ 153.6, (153.1), 149.1, 148.1, 126.7, (120.1), 119.5, 118.3, 111.5, 109.2, 81.9, (81.5), 56.1, 55.9, 46.4, (45.5), 40.2, (38.8), 28.3, 27.7; FT-IR (thin film, neat) ν_max_ 2977, 2937, 1702, 1521, 1407, 1246, 1160 cm^−1^; HRMS (EI+) found 318.1582 [calculated for C_17_H_22_N_2_O_2_ ([M]^+^): 318.1580].

(±)-*Allyl* *1-cyano-6,7-dimethoxy-3,4-dihydroisoquinoline-2(1H)-carboxylate* (**6b**) Yield 83%, white solid; m.p. 104 °C; ^1^H-NMR (400 MHz, CDCl_3_, at room temperature, 1.2:1 ratio amide bond) δ 6.74 (s, 1H), 6.40 (s, 1H), 5.92–6.03 (m, 2H), 5.23–5.37 (m, 1H), 5.21–5.29 (m, 1H), 4.63–4.70 (m, 2H), 4.33 (brs, 0.45H), 4.21 (brs, 0.55H), 3.89 (s, 3H), 3.87 (s, 3H), 3.44 (brs, 0.55H), 3.32 (brs, 0.45H), 2.88–2.96 (m, 1H), 2.73–2.78 (m, 1H); ^13^C-NMR (100 MHz, CDCl_3_, rotameric mixture, resonances for minor rotamer are enclosed in parenthesis) δ 154.4, (153.6), 149.2, 148.1, 131.9, (126.5), 126.2, 119.6, (119.1), (118.3), 118.1, 117.9, 111.4, 109.1, 67.0, 56.0, 55.9, 45.9, 39.9, (39.4), 27.6; FT-IR (thin film, neat) ν_max_ 3019, 2937, 1701, 1519, 1408, 1222, 1094 cm^-1^; HRMS (EI+) found 302.1264 [calculated for C_16_H_18_N_2_O_4_ ([M]^+^): 302.1267].

(±)-*Benzyl 1-cyano-6,7-dimethoxy-3,4-dihydroisoquinoline-2(1H)-carboxylate* (**6c**) Yield 87%, white solid; m.p. 127 °C; ^1^H-NMR (400 MHz, CDCl_3_, at room temperature, 1.2:1 ratio amide bond) δ 7.32–7.44 (m, 5H), 6.75 (brs, 0.55H), 6.71 (brs, 0.45H), 6.63 (brs, 1H), 6.05 (brs, 0.55H), 5.89 (brs, 0.45H), 5.20–5.26 (m, 2H), ; 4.36 (brs, 0.45H), 4.22 (brs, 0.55H), 3.87 (s, 6H), 3.44 (brs, 0.55H), 3.33 (brs, 0.45H), 2.84–2.98 (m, 1H), 2.50–2.80 (m, 1H); ^13^C-NMR (100 MHz, CDCl_3_, rotameric mixture, resonances for minor rotamer are enclosed in parenthesis) δ 154.7, (153.9), 149.3, 148.2, 135.6, (135.5), 128.5, 128.3, (128.2), 128.0, (126.6), 126.3, 119.7, (119.1), 118.0, 111.5, 109.2, (109.1), 68.4, (68.3), (56.1), 56.0, 46.1, 40.1, (39.6), 27.7, (27.6); FT-IR (thin film, neat) ν_max_ 3019, 2936, 1702, 1519, 1412, 1222, 1093 cm^−1^; HRMS (EI+) found 352.1427 [calculated for C_20_H_20_N_2_O_4_ ([M]^+^): 352.1423].

(±)-*Methyl 1-cyano-6,7-dimethoxy-3,4-dihydroisoquinoline-2(1H)-carboxylate* (**6d**) Yield 76%, colorless oil; ^1^H-NMR (400 MHz, CDCl_3_, at room temperature, 1.2:1 ratio amide bond) δ 6.74 (s, 1H), 6.63 (s, 1H), 6.03 (brs, 0.55H), 5.89 (brs, 0.45H), 4.33 (0.55H), 4.17 (brs, 0.45H), 3.88 (s, 3H), 3.87 (s, 3H), 3.81 (s, 3H), 3.39 (brs, 0.55H), 3.31 (brs, 0.45H), 2.86–2.95 (m, 1H), 2.75–2.77 (m, 0.55H), 2.71–2.73 (brs, 0.45H); ^13^C-NMR (100 MHz, CDCl_3_, rotameric mixture, resonances for minor rotamer are enclosed in parenthesis) δ 155.2, 149.2, 148.1, (126.6), 126.3, 119.7, (119.2), 118.0, 111.4, 109.1, 56.1, 56.0, 53.6, 46.0, 39.9, (39.5), 27.7, (27.5); FT-IR (thin film, neat) ν_max_ 3017, 2955, 1703, 1518, 1443, 1224, 1098 cm^−1^; HRMS (EI+) found 276.1102 [calculated for C_14_H_16_N_2_O_4_ ([M]^+^): 276.1110].

(±)-*Ethyl* *1-cyano-6,7-dimethoxy-3,4-dihydroisoquinoline-2(1H)-carboxylate* (**6e**) Yield 82%, colorless oil; ^1^H-NMR (400 MHz, CDCl_3_, at room temperature, 1.2:1 ratio amide bond) δ 6.78 (s, 1H), 6.66 (s, 1H), 6.03 (brs, 0.55H), 5.92 (brs, 0.45H), 4.13–4.25 (m, 3H), 3.87 (s, 3H), 3.86 (s, 3H), 3.39 (brs, 0.55H), 3.29 (brs, 0.45H), 2.91–2.95 (m, 0.45H), 2.86–2.91 (m, 0.55H), 2.76–2.78 (m, 0.55H), 2.72–2.74 (m, 0.45H), 1.36 (brs, 3H); ^13^C-NMR (100 MHz, CDCl_3_, rotameric mixture, resonances for minor rotamer are enclosed in parenthesis) δ 154.6, (153.9), 149.0, 148.0, 126.5, (126.2), 119.7, (119.2), 117.9, 111.3, 109.0, 62.5, 56.0, 55.8, 45.8, 39.7, (39.2), 27.5, 14.6; FT-IR (thin film, neat) ν_max_ 3020, 2939, 1700, 1519, 1418, 1222, 1098 cm^−1^; HRMS (EI+) found 290.1267 [calculated for C_15_H_18_N_2_O_4_ ([M]^+^): 290.1267].

(±)-*Phenyl 1-cyano-6,7-dimethoxy-3,4-dihydroisoquinoline-2(1H)-carboxylate* (**6f**) Yield 74%, white foam; ^1^H-NMR (600 MHz, CDCl_3_) δ 7.38-7.41 (m, 2H), 7.24–7.27 (m, 1H), 7.15–7.18 (m, 2H), 6.79 (s, 1H), 6.69 (s, 1H), 6.13 (brs, 0.45H), 6.09 (brs, 0.55H), 4.39–4.41 (m, 1H), 3.90 (s, 6H), 3.61–3.65 (m, 0.55H), 3.41–3.46 (m, 0.45H), 2.98–3.08 (m, 1H), 2.81–2.88 (m, 1H); ^13^C-NMR (150 MHz, CDCl_3_) δ 171.2, 153.5, (152.8), 150.9, (150.8), (149.72), 149.66, 148.6, 129.5, (126.1), 126.0, (121.7), 121.6, 119.8, (119.2), 117.9, (111.8), 111.7, 109.4, (109.2), 56.2, 56.1, (46.6), 46.2, 40.7, (39.9), 27.7, (27.5); FT-IR (thin film, neat) ν_max_ 3018, 2938, 1723, 1520, 1411, 1199, 1119, 754 cm^−1^; HRMS (FAB+) found 338.1271 [calculated for C_19_H_18_N_2_O_4_ ([M]^+^): 338.1267].

*(±)-6,7-dimethoxy-2-(methylsulfonyl)-1,2,3,4-tetrahydroisoquinoline-1-carbonitrile* (**6g**) Yield 61%, white solid; m.p. 148 °C; ^1^H-NMR (400 MHz, CDCl_3_) δ 6.71 (s, 1H), 6.64 (s, 1H), 5.75 (s, 1H), 4.00–4.04 (m, 1H), 3.870 (s, 3H), 3.866 (s, 3H), 3.29–3.36 (m, 1H), 3.07 (s, 3H), 3.03–3.11 (m, 1H), 2.78–2.83 (m, 1H); ^13^C-NMR (100 MHz, CDCl_3_) δ 149.5, 148.3, 125.2, 118.7, 117.0, 111.6, 109.0, 56.2, 56.0, 46.9, 40.8, 37.7, 27.9; FT-IR (thin film, neat) ν_max_ 3015, 2937, 1519, 1343, 1228, 1153 cm^−1^; HRMS (EI+) found 296.0833 [calculated for C_13_H_16_N_2_O_4_S ([M]^+^): 296.0831].

(±)-*6,7-dimethoxy-2-tosyl-1,2,3,4-tetrahydroisoquinoline-1-carbonitrile* (**6h**) Yield 59%, colorless oil; ^1^H-NMR (400 MHz, CDCl_3_) δ 7.78 (d, *J* = 8.4 Hz, 2H), 7.35 (d, *J* = 8.4 Hz, 2H), 6.66 (s, 1H), 6.59 (s, 1H), 5.80 (s, 1H), 4.06 (dd, *J* = 12.4, 6.0 Hz, 1H), 3.87 (s, 3H), 3.85 (s, 3H), 3.05 (td, *J* = 12.4, 3.6 Hz, 1H), 3.03 (td, *J* = 16.0, 6.0 Hz, 1H), 2.72 (dd, *J* = 16.0, 3.6 Hz, 1H), 2.44 (s, 3H); ^13^C-NMR (100 MHz, CDCl_3_) δ 149.3, 148.1, 144.4, 134.2, 129.8, 127.5, 125.3, 119.4, 116.1, 111.5, 108.9, 56.0, 55.9, 46.9, 40.9, 27.6, 21.7; FT-IR (thin film, neat) ν_max_ 3018, 2936, 1519, 1348, 1228, 1162 cm^−1^; HRMS (EI+) found 372.1143 [calculated for C_19_H_20_N_2_O_4_S ([M]^+^): 372.1114].

(±)-*6,7-dimethoxy-2-((2-nitrophenyl)sulfonyl)-1,2,3,4-tetrahydronaphthalene-1-carbonitrile* (**6i**) Yield 82%, light yellow foam; ^1^H-NMR (400 MHz, CDCl_3_) δ 8.11 (d, *J* = 9.2 Hz, 1H), 7.71–7.78 (m, 3H), 6.72 (s, 1H), 6.61 (s, 1H), 5.87 (s, 1H), 4.19 (ddd, *J* = 14.0, 6.0, 1.6 Hz, 1H), 3.89 (s, 3H), 3.86 (s, 3H), 3.52 (ddd, *J* = 14.0, 12.4, 4.0 Hz, 1H), 3.05 (ddd, *J* = 16.4, 12.4, 6.0 Hz, 1H), 2.78 (ddd, *J* = 16.4, 4.0, 1.6 Hz, 1H); ^13^C-NMR (100 MHz, CDCl_3_) δ 149.6, 148.4, 147.9, 134.5, 132.3, 131.9, 130.9, 125.3, 124.7, 119.1, 116.8, 111.6, 108.8, 56.2, 56.1, 47.3, 41.9, 27.8; FT-IR (thin film, neat) ν_max_ 3021, 2938, 1543, 1520, 1370, 1168, 1116, 771 cm^−1^; HRMS (EI+) found 403.0834 [calculated for C_18_H_17_N_3_O_6_S ([M]^+^): 403.0838].

(±)-*1-Cyano-N,N-diethyl-6,7-dimethoxy-3,4-dihydroisoquinoline-2(1H)-carboxamide* (**6j**) Yield 86%, white solid; m.p. 136 °C; ^1^H-NMR (600 MHz, CDCl_3_) δ 6.74 (1H), 6.63 (s, 1H), 5.49 (s, 1H), 3.89 (s, 3H), 3.87 (s, 3H), 3.72 (dd, *J* = 13.8, 6.0 Hz, 1H), 3.45 (dd, *J* = 13.8, 12.6 Hz, 1H), 3.30 (q, *J* = 7.2 Hz, 2H), 3.28 (q, *J* = 7.2 Hz, 2H), 3.02 (ddd, *J* = 16.2, 12.6, 6.0 Hz, 1H), 2.73 (d, *J* = 16.2 Hz, 1H), 1.18 (t, *J* = 7.2 Hz, 6H); ^13^C-NMR (150 MHz, CDCl_3_) δ 162.9, 149.4, 148.3, 126.4, 121.2, 119.0, 111.7, 109.5, 56.2, 56.0, 48.5, 44.0, 41.9, 27.6, 13.2; FT-IR (thin film, neat) ν_max_ 2970, 2936, 1648, 1520, 1463, 1421, 1370, 1266, 1228, 1120, 754 cm^−1^; HRMS (FAB+) found 318.1811 [calculated for C_17_H_24_N_3_O_3_ ([M + H]^+^): 318.1818].

(±)-*2-Acetyl-6,7-dimethoxy-1,2,3,4-tetrahydroisoquinoline-1-carbonitrile* (**6k**) Yield 25%, white solid; m.p. 200 °C; ^1^H-NMR (400 MHz, CDCl_3_) δ 6.76 (s, 1H), 6.64 (s, 1H), 6.40 (s, 1H), 4.44 (dd, *J* = 14.0, 5.2 Hz, 1H), 3.89 (s, 3H), 3.87 (s, 3H), 3.58 (ddd, *J* = 14.0, 12.0, 5.2 Hz, 1H), 2.98 (ddd, *J* = 16.0, 12.0, 5.2 Hz, 1H), 2.76 (dd, *J* = 16.0, 5.2 Hz, 1H), 2.21 (s, 3H); ^13^C-NMR (100 MHz, CDCl_3_) δ 169.3, 149.2, 148.3, 125.9, 119.9, 117.9, 111.3, 109.3, 56.2, 56.0, 43.3, 42.2, 28.1, 21.5; FT-IR (thin film, neat) ν_max_ 2936, 1652, 1518, 1409, 1253, 1222, 1117 cm^−1^; HRMS (EI+) found 260.1159 [calculated for C_14_H_16_N_2_O_3_ ([M]^+^): 260.1161].

(±)-*2-Benzoyl-6,7-dimethoxy-1,2,3,4-tetrahydroisoquinoline-1-carbonitrile* (**6l**) Yield 17%, white solid; m.p. 210 °C; ^1^H-NMR (400 MHz, CDCl_3_) δ 7.43–7.47 (m, 5H), 6.80 (brs, 1H), 6.63 (s, 1H), 6.39 (brs, 1H), 3.93–4.02 (m, 1H), 3.91 (s, 3H), 3.85 (s, 3H), 3.52 (brs, 1H), 2.98 (brs, 1H), 2.72 (d, *J* = 15.2 Hz, 1H); ^13^C-NMR (100 MHz, CDCl_3_) δ 170.8, 149.4, 148.5, 133.9, 130.8, 128.8, 127.1, 119.6, 117.9, 111.5, 109.4, 56.2, 56.1, 44.4, 43.4, 28.4; FT-IR (thin film, neat) ν_max_ 2935, 1641, 1518, 1406, 1253, 1223, 1141, 1108 cm^−1^; HRMS (EI+) found 322.1319 [calculated for C_19_H_18_N_2_O_3_ ([M]^+^): 322.1317].

(±)-*tert*-*Butyl 1-cyano-6-methoxy-3,4-dihydroisoquinoline-2(1H)-carboxylate* (**6m**) Yield 78%, colorless oil; ^1^H-NMR (400 MHz, CDCl_3_, at room temperature, 1.1:1 ratio amide bond) δ 7.23 (brs, 0.48H), 7.21 (brs, 0.52H), 6.84 (d, *J* = 2.0 Hz, 0.52H), 6.82 (d, *J* = 2.0 Hz, 0.48H), 6.70 (d, *J* = 2.0 Hz, 1H), 6.03 (brs, 0.48H), 5.80 (brs, 0.52H), 4.20 (brs, 0.52H), 4.03 (brs, 0.48H), 3.80 (s, 3H), 3.41 (brs, 0.48H), 3.28 (brs, 0.52H), 2.94 (dd, *J* = 10.4, 5.6 Hz, 0.48H), 2.90 (dd, *J* = 10.4, 5.6 Hz, 0.52H), 2.83 (t, *J* = 4.0 Hz, 0.52H), 2.79 (t, *J* = 4.0 Hz, 0.48H), 1.53 (s, 9H); ^13^C-NMR (100 MHz, CDCl_3_, rotameric mixture, resonances for minor rotamer are enclosed in parenthesis) δ 159.4, (153.7), 153.2, 135.9, 128.0, (120.6), 120.0, 118.3, 113.8, 113.3, 82.0, (81.6), 55.3, (46.2), 45.3, (40.1), 38.7, 28.5, 28.3; FT-IR (thin film, neat) ν_max_ 2976, 1703, 1612, 1505, 1404, 1238, 1159 cm^−1^; HRMS (EI+) found 288.1472 [calculated for C_16_H_20_N_2_O_3_ ([M]^+^): 288.1474].

(±)-*tert*-*Butyl 1-cyano-7-methoxy-3,4-dihydroisoquinoline-2(1H)-carboxylate* (**6n**) Yield 62%, white solid; m.p. 114 °C; ^1^H-NMR (400 MHz, CDCl_3_, at room temperature, 1.1:1 ratio amide bond) δ 7.10 (d, *J* = 8.0 Hz, 1H), 6.85 (d, *J* = 8.8 Hz, 1H), 6.81 (s, 1H), 6.06 (brs, 0.52H), 5.82 (brs, 0.48H), 4.26 (brs, 0.52H), 4.10 (brs, 0.48H), 3.81 (s, 3H), 2.90 (dd, *J* = 10.4, 5.6 Hz, 0.48H), 2.86 (dd, *J* = 10.4, 5.6 Hz, 0.52H), 2.78 (t, *J* = 4.0 Hz, 0.52H), 2.74 (t, *J* = 4.0 Hz, 0.48H), 1.57 (s, 9H); ^13^C-NMR (100 MHz, CDCl_3_, rotameric mixture, resonances for minor rotamer are enclosed in parenthesis) δ 158.6, 150.6, 139.4, 130.6, 126.7, 118.3, 115.7, 111.6, 47.1, (46.1), (40.7), 39.4, 28.7, 27.7; FT-IR (thin film, neat) ν_max_ 2976, 2935, 1703, 1507, 1404, 1251, 1161 cm^−1^; HRMS (EI+) found 288.1471 [calculated for C_16_H_20_N_2_O_3_ ([M]^+^): 288.1474].

(±)-*tert*-*Butyl 1-cyano-6,8-dimethoxy-3,4-dihydroisoquinoline-2(1H)-carboxylate* (**6o**) Yield 95%, colorless oil; ^1^H-NMR (400 MHz, CDCl_3_, at room temperature, 1.1:1 ratio amide bond) δ 6.35 (s, 1H), 6.28 (s, 1H), 6.10 (brs, 0.48H), 5.82 (brs, 0.52H), 4.26 (brs, 0.52H), 4.10 (brs, 0.52H), 3.89 (brs, 3H), 3.79 (s, 3H), 3.38 (brs, 0.48H), 3.24 (brs, 0.52H), 2.90 (brs, 1H), 2.73–2.77 (m, 1H), 1.53 (brs, 0.52H), 1.51 (brs, 0.48H); ^13^C-NMR (100 MHz, CDCl_3_, rotameric mixture, resonances for minor rotamer are enclosed in parenthesis) δ 160.7, 156.9, 153.4, 136.7, (136.3), 118.1, 104.6, 96.8, 81.9, (81.5), 55.8, 55.5, 42.7, (41.8), (39.9), 38.6, 28.4; FT-IR (thin film, neat) ν_max_ 2975, 1703, 1609, 1405, 1160 cm^−1^; HRMS (EI+) found 318.1580 [calculated for C_17_H_22_N_2_O_4_ ([M]^+^): 318.1580].

(±)-*tert-Butyl 1-cyano-7-fluoro-3,4-dihydroisoquinoline-2(1H)-carboxylate* (**6p**) Yield 67%, white solid; m.p. 124 °C; ^1^H-NMR (400 MHz, CDCl_3_, at room temperature, 1.1:1 ratio amide bond) δ 7.16–7.19 (m, 1H), 7.00-7.05 (m, 2H), 6.08 (brs, 0.48H), 5.84 (brs, 0.52H), 4.29 (brs, 0.52H), 4.13 (brs, 0.48H), 3.38 (brs, 0.48H), 3.24 (brs, 0.52H), 2.93 (dd, *J* = 10.4, 5.6 Hz, 0.48H), 2.89 (dd, *J* = 10.4, 5.6 Hz, 0.52H), 2.83 (t, *J* = 3.6 Hz, 0.52H), 2.79 (t, *J* = 3.6 Hz, 0.48H), 1.53 (s, 9H); ^13^C-NMR (100 MHz, CDCl_3_, rotameric mixture, resonances for minor rotamer are enclosed in parenthesis) δ 162.5, 160.0, (153.7), 153.1, 131.0, 130.3, (129.6), 117.7, 116.3, (116.1), 113.9, (113.7), (82.6), 82.1, (46.6), 45.7, (40.2), 38.9, 28.5, 27.7; FT-IR (thin film, neat) ν_max_ 2978, 1702, 1503, 1404, 1246, 1161 cm^−1^; HRMS (EI+) found 276.1273 [calculated for C_15_H_17_FN_2_O_2_ ([M]^+^): 276.1274].

(±)-*tert-Butyl 7-bromo-1-cyano-3,4-dihydroisoquinoline-2(1H)-carboxylate* (**6q**) Yield 68%, white solid; m.p. 157 °C; ^1^H-NMR (400 MHz, CDCl_3_, at room temperature, 1.2:1 ratio amide bond) δ 7.48 (brs, 1H), 7.41 (dd, *J* = 8.0, 1.6 Hz, 1H), 7.08 (d, *J* = 8.0 Hz, 1H); 6.08 (brs, 0.45H), 5.84 (brs, 0.55H), 4.28 (brs, 0.55H), 4.12 (brs, 0.45H), 3.37 (brs, 0.45H), 3.24 (brs, 0.55H), 2.77–2.93 (m, 2H), 1.53 (s, 9H); ^13^C-NMR (100 MHz, CDCl_3_, rotameric mixture, resonances for minor rotamer are enclosed in parenthesis) δ 132.0, 131.1, 130.0, 120.6, 117.7, 46.3, (45.4), (40.1), 38.7, 28.5, 27.9; FT-IR (thin film, neat) ν_max_ 3327, 3005, 2954, 1743, 1680, 1613, 1513, 1392, 1262, 1202, 1149, 1097, 756 cm^−1^; HRMS (EI+) found 336.0472 [calculated for C_15_H_17_BrN_2_O_2_ ([M]^+^): 336.0473].

(±)-*tert-Butyl 1-cyano-3,4-dihydroisoquinoline-2(1H)-carboxylate* (**6r**) Yield 96%, white solid; m.p. 87–77 °C; ^1^H-NMR (600 MHz, CDCl_3_, at room temperature, 1.2:1 ratio amide bond) δ 7.27–7.32 (m, 3H), 7.20–7.21 (m, 1H), 6.10 (brs, 0.45H), 5.87 (brs, 0.55H), 4.26 (brs, 0.55H), 4.09 (brs, 0.45H), 3.42 (brs, 0.45H), 3.28 (brs, 0.45H), 2.93–2.98 (m, 1H), 2.86 (t, *J* = 4.2 Hz, 0.55H), 2.84 (t, *J* = 4.2 Hz, 0.45H), 1.54 (9s, 9H); ^13^C-NMR (150 MHz, CDCl_3_, rotameric mixture, resonances for minor rotamer are enclosed in parenthesis) δ 154.0, (153.4), 134.8, (134.6), 129.5, 128.8, 128.3, 127.24, 127.17, 118.3, 82.3, (81.9), (46.7), 45.7, 40.2, (38.8), 28.4, 28.2; FT-IR (thin film, neat) ν_max_ 2977, 1701, 1404, 1161 cm^−1^; HRMS (FAB+) found 259.1446 [calculated for C_15_H_19_N_2_O_2_ ([M + H]^+^): 259.1447].

(±)-*tert-Butyl 1-(hydroxymethyl)-6,7-dimethoxy-3,4-dihydroisoquinoline-2(1H)-carboxylate* (**8**). To a cooled (−78 °C) solution of α-cyano tetrahydroisoquinoline (±)-**6a** (176.5 mg, 0.554 mmol) in dry toluene (5.60 mL), a solution of diisobutylaluminum hydride (DIBAL-H^®^, 1.39 mmol, 1.39 mL; 1.0 M solution in toluene) was added dropwise. The reaction mixture was stirred for 30 min at −78 °C under argon atmosphere, then quenched with saturated aqueous Rochelle’s salt solution (5 mL) and diluted with EtOAc (5 mL) and the layers were separated. The aqueous layer was extracted with EtOAc (10 mL × 2), and the combined organic layer was washed with brine (5 mL), dried over Na_2_SO_4_, filtered, and concentrated in vacuo to afford the unstable aldehyde (±)-**7**, which was used directly in the next reaction without further purification.

To an ice-cooled (0 °C) solution of aldehyde (±)-**7** in dry MeOH (5.60 mL), NaBH_4_ (62.8 mg, 1.66 mmol) was added portionwise. The reaction mixture was stirred for 30 min at 0 °C, then quenched with saturated aqueous NH_4_Cl solution (5 mL) and diluted with EtOAc (5 mL), and the layers were separated. The aqueous layer was extracted two times with EtOAc (10 mL), and the combined organic layer was washed with brine (5 mL), dried over sodium sulfate, filtered, and concentrated under reduced pressure. Purification of the crude material using flash column chromatography on silica gel, using hexanes/EtOAc (2:1 to 1:1) as an eluent, provided (±)-*N*-Boc calycotomine (**8**) (68.1 mg, 38% from (±)-**6a** over two steps) as a colorless oil; ^1^H-NMR (400 MHz, CDCl_3_, at room temperature, 2:1 ratio amide bond) δ 6.67 (s, 1H), 6.62 (s, 1H), 5.22 (brs, 0.67H), 5.07 (brs, 0.33H), 3.86 (s, 6H), 3.67–3.80 (m, 2H), 3.43 (brs, 0.67H), 3.26 (0.33H), 2.71–2.91 (m, 4H), 1.50 (s, 9H); ^13^C-NMR (100 MHz, CDCl_3_, rotameric mixture, resonances for minor rotamer are enclosed in parenthesis) δ 156.0, (154.7), 147.6, 147.1, (127.0), 126.7, 125.3, 111.1, 110.1, 79.9, 66.5, (65.5), (56.3), 55.8, 55.7, 56.0, 39.4, (37.6), 28.3, 28.1; IR (thin film, neat) *ν*_max_ 3448, 2934, 1670, 1516, 1422, 1365, 1247, 1160 cm^−1^; HRMS (EI+) found 323.1731 [calculated for C_17_H_25_NO_5_ ([M]^+^): 323.1733].

## 4. Conclusions

In conclusion, we have developed a highly mild and efficient metal-free cyanation at the α-position of a variety of *N*-acyl/sulfonyl and electronically diverse tetrahydroisoquinolines (THIQs) with (*n*-Bu)_3_SnCN under oxidative reaction conditions. *N*-Acyl/sulfonyl iminium ions generated by DDQ oxidation were found to be very effective and compatible with an electron-rich cyanide nucleophile. This reaction provides a convenient method for the synthesis of structurally diverse THIQ natural products and pharmacologically useful compounds.

## Figures and Tables

**Table 1:**
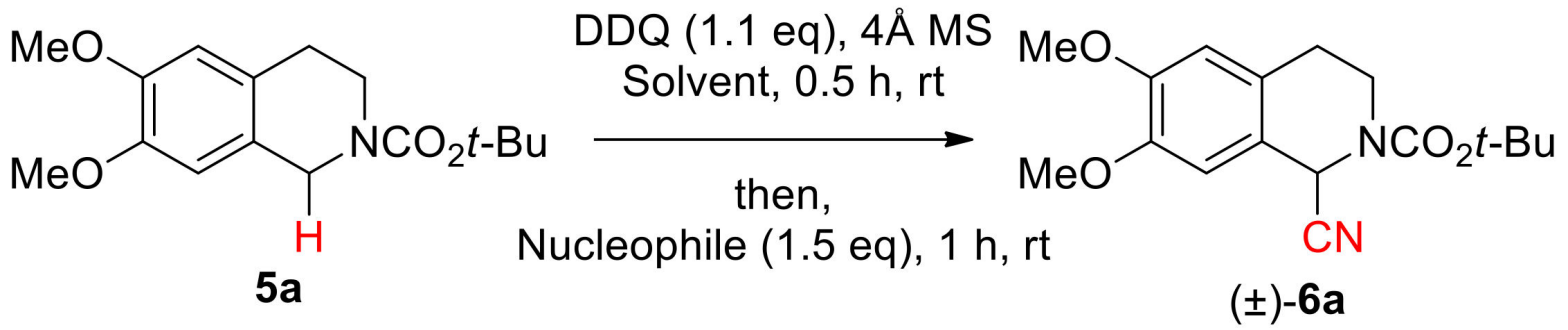
Optimization of DDQ-promoted α-cyanation of *N*-Boc THIQ **5a** ^a^.

Entry	Nucleophile	Solvent	Yield (%) ^b^
1	TMSCN	DCM	95
2	TBSCN	DCM	21
3	Zn(CN)_2_	DMF	10
4	(*n*-Bu)_3_SnCN	DCM	99
5	(*n*-Bu)_3_SnCN	EtOAc	96
6	(*n*-Bu)_3_SnCN	toluene	95
7	(*n*-Bu)_3_SnCN	acetone	95
8	(*n*-Bu)_3_SnCN	THF	87
9	(*n*-Bu)_3_SnCN	MeCN	78
10	(*n*-Bu)_3_SnCN	DMF	56

^a^ Reaction conditions: N-Boc THIQ **5a** (0.3 mmol, 1 equivalent), DDQ (1.1 equivalent) in solvent (3.0 mL) at room temperature under an argon atmosphere for 0.5 h, then cyanide nucleophile (1.5 equivalent) for 1 h. ^b^ Based on the isolated product using chromatography after purification.

## Supplementary Materials

The following are available online at http://www.mdpi.com/1420-3049/23/12/3223/s1. The ^1^H and ^13^C-NMR spectra can be found in the SI.
