# Supplementary material for: DDQ-Promoted Mild and Efficient Metal-Free Oxidative α-Cyanation of N-Acyl/Sulfonyl 1,2,3,4-Tetrahydroisoquinolines
Source: Molecules. 2018 Dec 6;23(12):3223. doi: 10.3390/molecules23123223 (PMC6321290; doi:10.3390/molecules23123223)
Supplement: Supplementary file 1 [file molecules-23-03223-s001.pdf]

-Supporting Information-

DDQ-Promoted Mild and Efficient Metal-Free Oxidative α-Cyanation of *N*-Acyl/Sulfonyl 1,2,3,4-Tetrahydroisoquinolines

Hong Pyo Kim[1,†], Heesun Yu[1,†], Hyoungsu Kim[1], Seok-Ho Kim[2,*] and Dongjoo Lee[1,*]

[1] College of Pharmacy, Research Institute of Pharmaceutical Science and Technology (RIPST), Ajou University, 206 Worldcup-ro, Yeongtong-gu, Suwon 16499, Republic of Korea
[2] Department of Pharmacy, College of Pharmacy and Institute of Pharmaceutical Sciences, CHA University, 120 Haeryong-ro, Pocheon 11160, Gyeonggi-do, Republic of Korea

E-mail: ksh3410@cha.ac.kr and dongjoo@ajou.ac.kr

(S01-S52)
Copies of ^{1}H and ^{13}C NMR

Table of Contents

Compound **5b**: ^{1}H NMR and ^{13}C NMR **S5-S6**

Compound **5f**: ^{1}H NMR and ^{13}C NMR **S7-S8**

Compound **5i**: ^{1}H NMR and ^{13}C NMR **S9-S10**

Compound **5j**: ^{1}H NMR and ^{13}C NMR **S11-S12**

Compound **5p**: ^{1}H NMR and ^{13}C NMR **S13-S14**

Compound (±)-**6a**: ^{1}H NMR and ^{13}C NMR **S15-S16**

Compound (±)-**6b**: ^{1}H NMR and ^{13}C NMR **S17-S18**

Compound (±)-**6c**: ^{1}H NMR and ^{13}C NMR **S19-S20**

Compound (±)-**6d**: ^{1}H NMR and ^{13}C NMR **S21-S22**

Compound (±)-**6e**: ^{1}H NMR and ^{13}C NMR **S23-S24**

Compound (±)-**6f**: ^{1}H NMR and ^{13}C NMR **S25-S26**

Compound (±)-**6g**: ^{1}H NMR and ^{13}C NMR **S27-S28**

Table of Contents

Compound (±)-**6h**: ^{1}H NMR and ^{13}C NMR	**S29**-**S30**
Compound (±)-**6i**: ^{1}H NMR and ^{13}C NMR	**S31**-**S32**
Compound (±)-**6j**: ^{1}H NMR and ^{13}C NMR	**S33**-**S34**
Compound (±)-**6k**: ^{1}H NMR and ^{13}C NMR	**S35**-**S36**
Compound (±)-**6l**: ^{1}H NMR and ^{13}C NMR	**S37**-**S38**
Compound (±)-**6m**: ^{1}H NMR and ^{13}C NMR	**S39**-**S40**
Compound (±)-**6n**: ^{1}H NMR and ^{13}C NMR	**S41**-**S42**
Compound (±)-**6o**: ^{1}H NMR and ^{13}C NMR	**S43**-**S44**
Compound (±)-**6p**: ^{1}H NMR and ^{13}C NMR	**S45**-**S46**
Compound (±)-**6q**: ^{1}H NMR and ^{13}C NMR	**S47**-**S48**
Compound (±)-**6r**: ^{1}H NMR and ^{13}C NMR	**S49**-**S50**
Compound (±)-**8**: ^{1}H NMR and ^{13}C NMR	**S51**-**S52**

Substrate	Reference	Substrate	Reference	Substrate	Reference
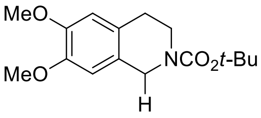 **5a**	*Chem. Commun.* **2014**, *50*, 1238-1240	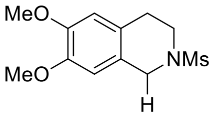 **5g**	*Tetrahedron Lett.* **2001**, *42*, 6251-6253	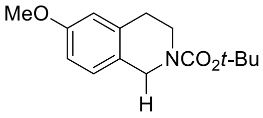 **5m**	*Adv. Synth. Catal.* **2016**, *358*, 4049-4056
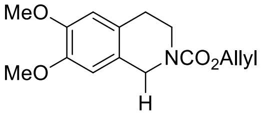 **5b**	**Unknown**	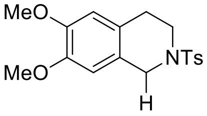 **5h**	*Tetrahedron Lett.* **2010**, *51*, 435-438	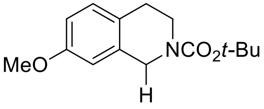 **5n**	*Bioorg. Med. Chem.* **2008**, *16*, 2499-2512
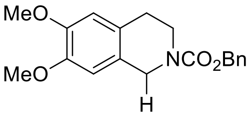 **5c**	*Chem. Commun.* **2005**, *41*, 4465-4467	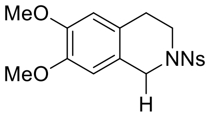 **5i**	**Unknown**	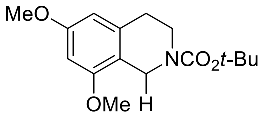 **5o**	*Adv. Synth. Catal.* **2016**, *358*, 4049-4056
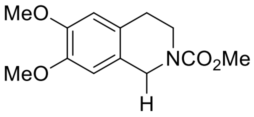 **5d**	*Bioorg. Med. Chem.* **2004**, *12*, 871-882	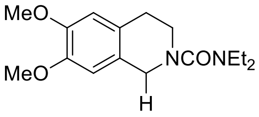 **5j**	**Unknown**	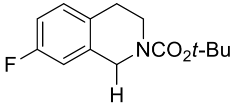 **5p**	**Unknown**
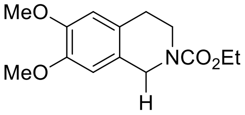 **5e**	*Helv. Chim. Acta* **1980**, *63*, 938-961	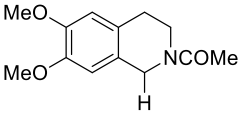 **5k**	*Bioorg. Med. Chem. Lett.* **2010**, *20*, 4999-5003	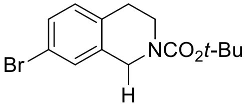 **5q**	*Bioorg. Med. Chem. Lett.* **2018**, *28*, 3050-3056
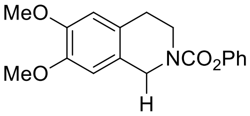 **5f**	**Unknown**	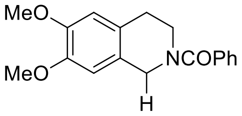 **5l**	*Synthetic Commun.* **1992**, *22*, 3235-3242	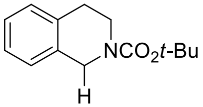 **5r**	*J. Org. Chem.* **2011**, *76*, 6703-6714

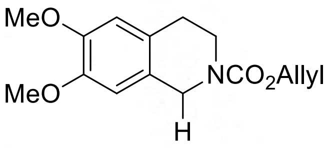

5b

(400MHz, ^{1}H NMR, $CDCl_3$)

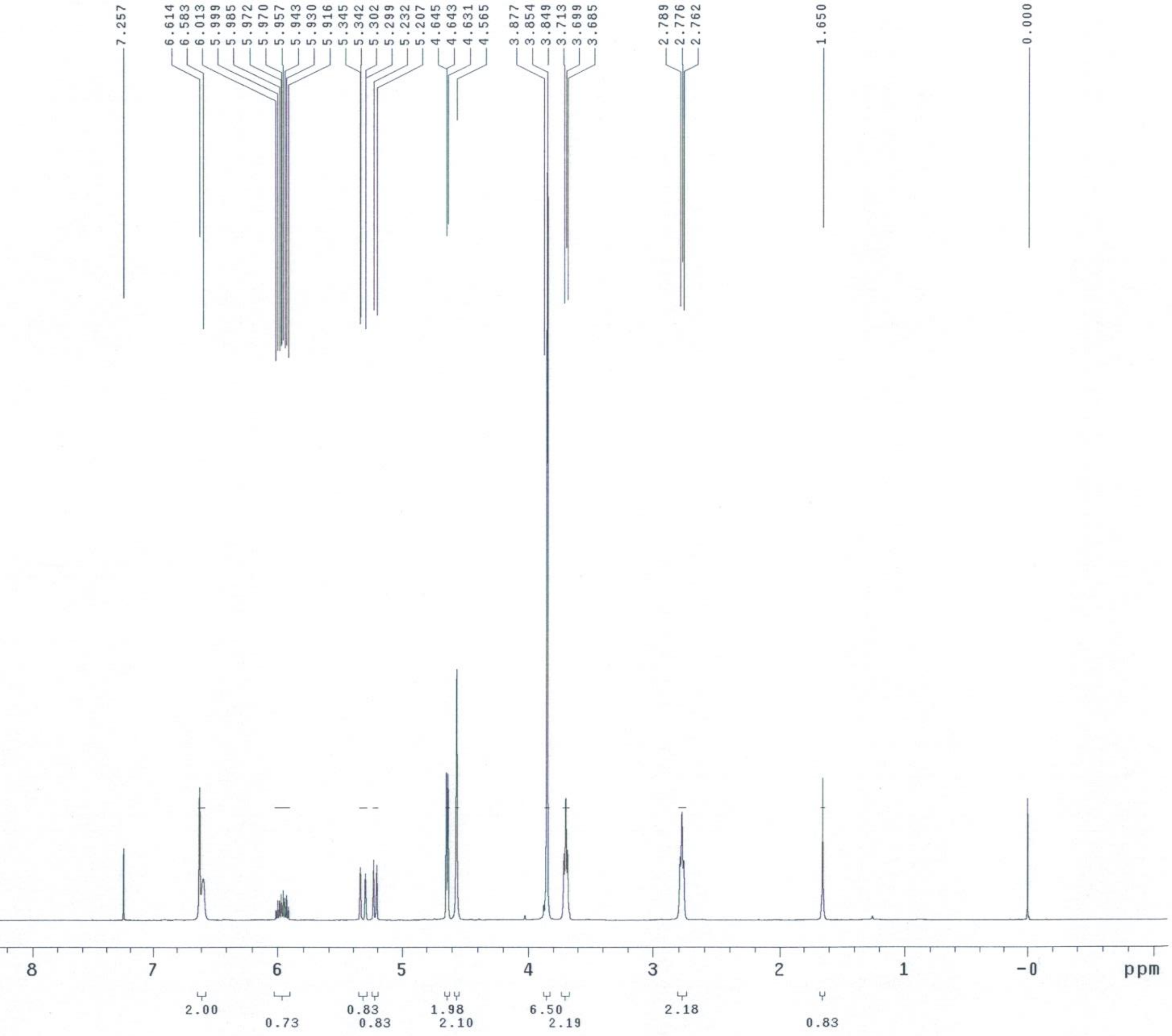

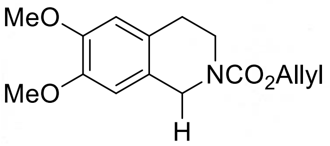

5b

(100MHz, ^{13}C NMR, $CDCl_3$)

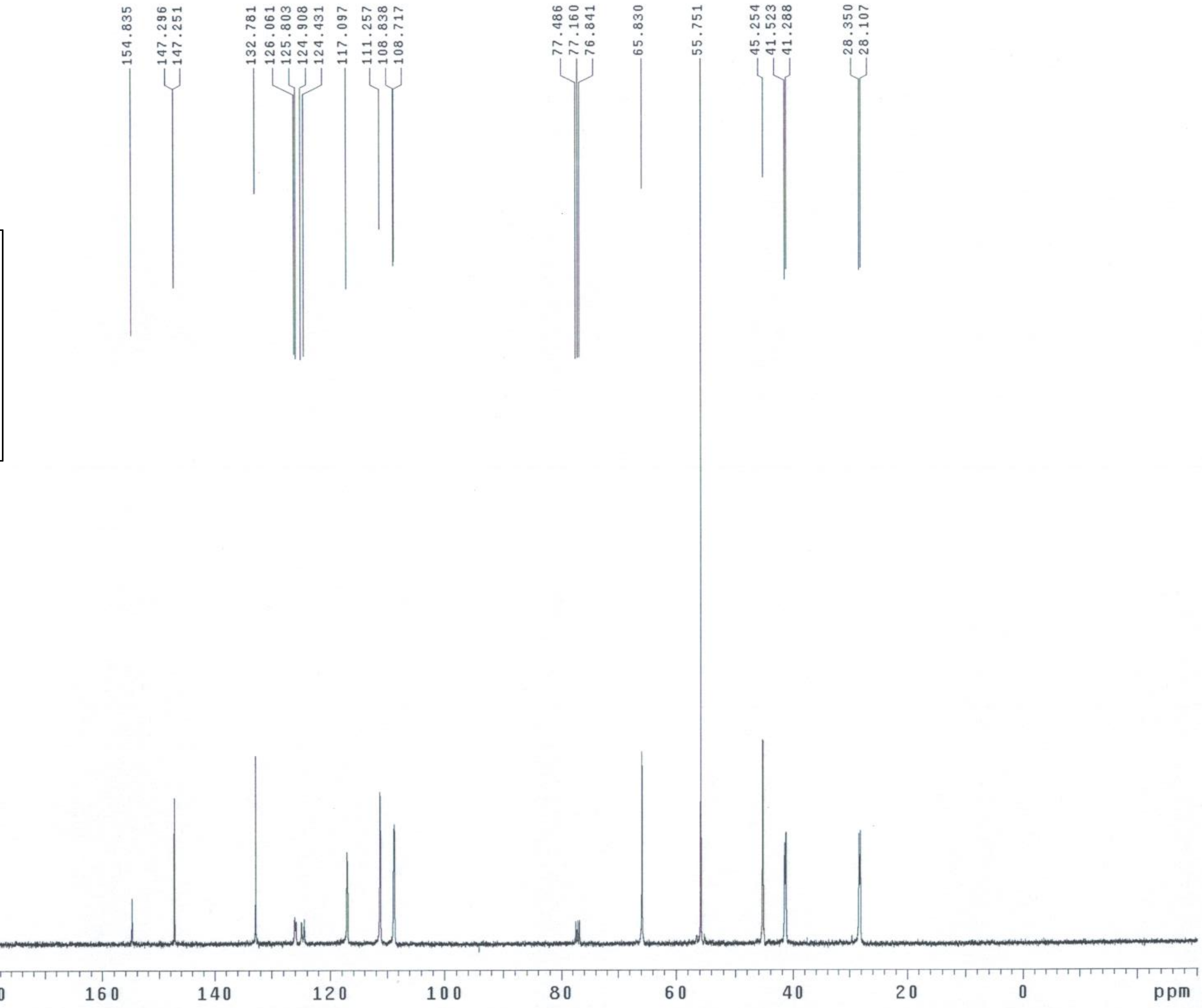

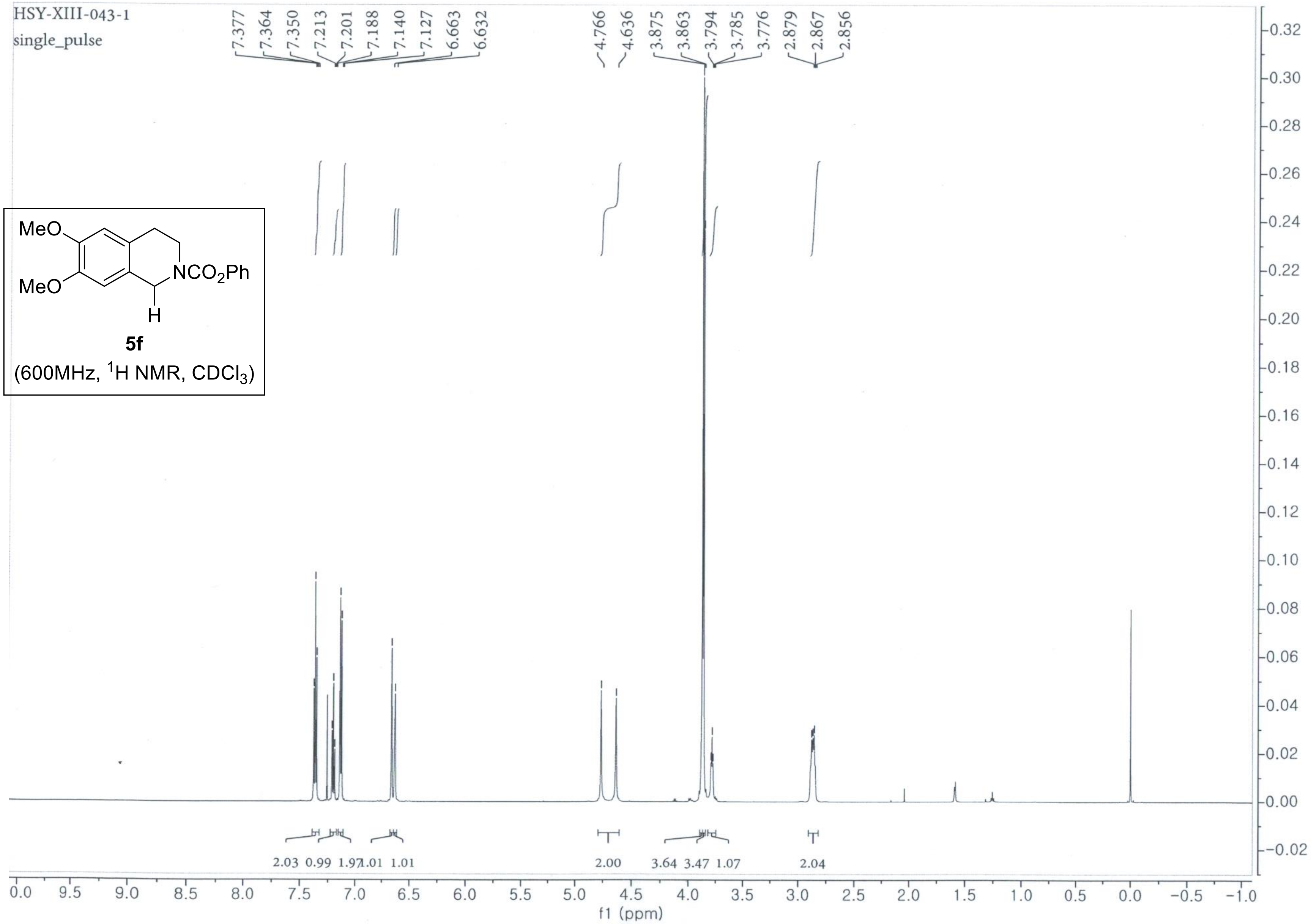
HSY-XIII-043-1
single_pulse
7.377
7.364
7.350
7.213
7.201
7.188
7.140
7.127
6.663
6.632
4.766
4.636
3.875
3.863
3.794
3.785
3.776
2.879
2.867
2.856
MeO
MeO
NCO2Ph
H
5f
(600MHz, 1H NMR, CDCl3)
2.03 0.99 1.97 1.01 1.01
2.00
3.64 3.47 1.07
2.04
f1 (ppm)

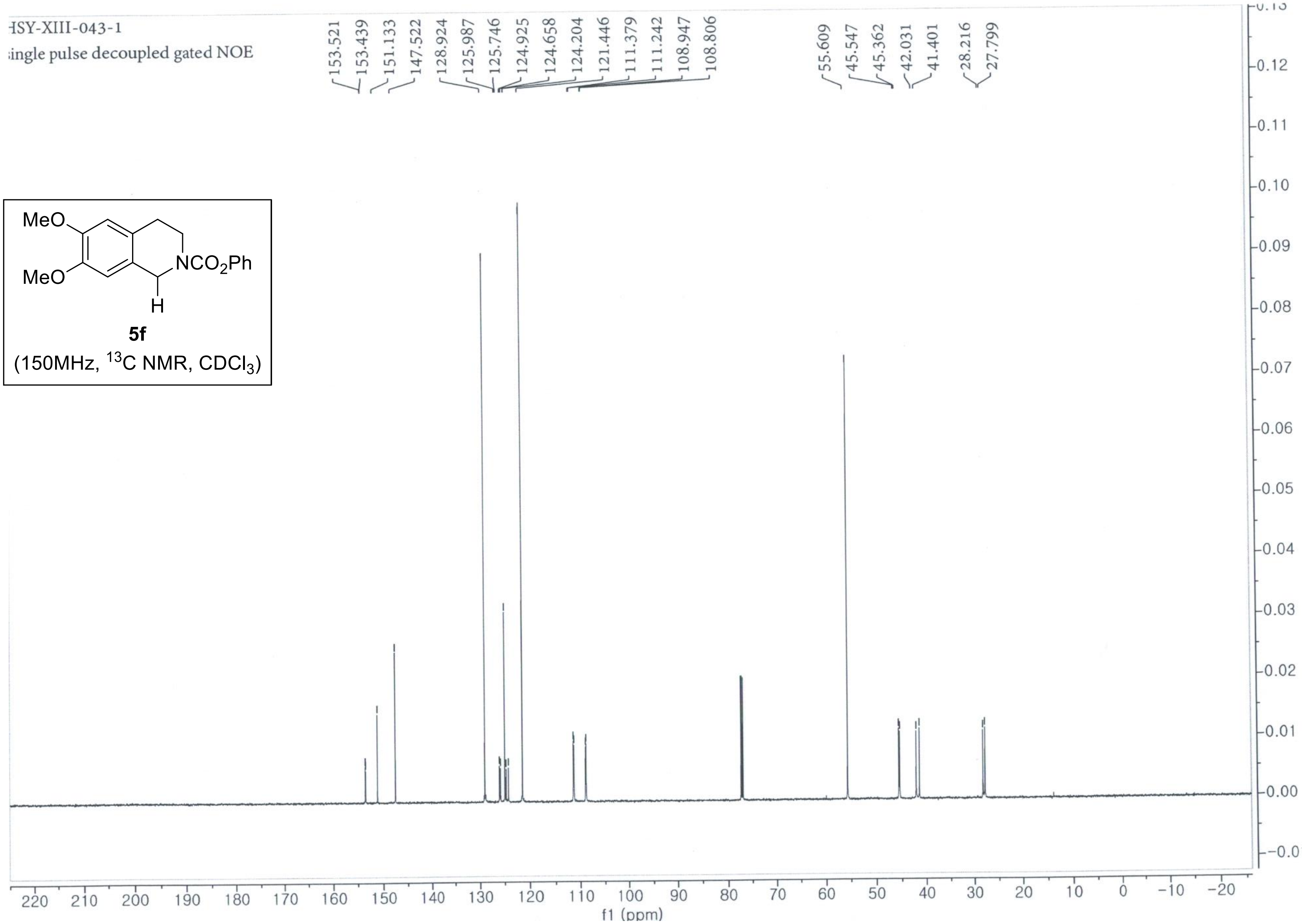
HSY-XIII-043-1
single pulse decoupled gated NOE
153.521
153.439
151.133
147.522
128.924
125.987
125.746
124.925
124.658
124.204
121.446
111.379
111.242
108.947
108.806
55.609
45.547
45.362
42.031
41.401
28.216
27.799
MeO
MeO
NCO2Ph
H
5f
(150MHz, 13C NMR, CDCl3)
f1 (ppm)

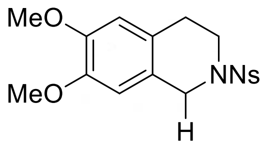

5i
(400MHz, ^{1}H NMR, $CDCl_3$)

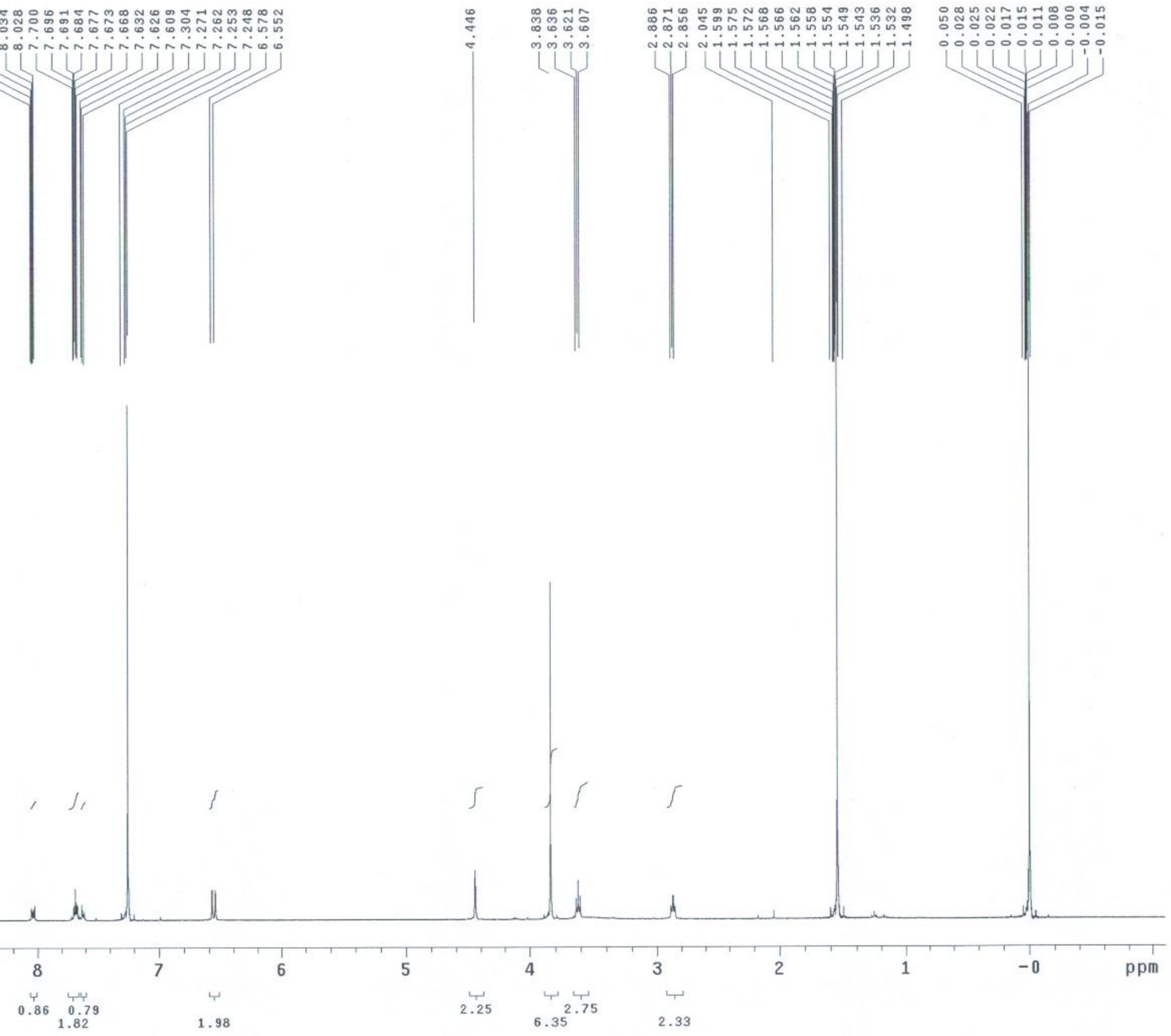

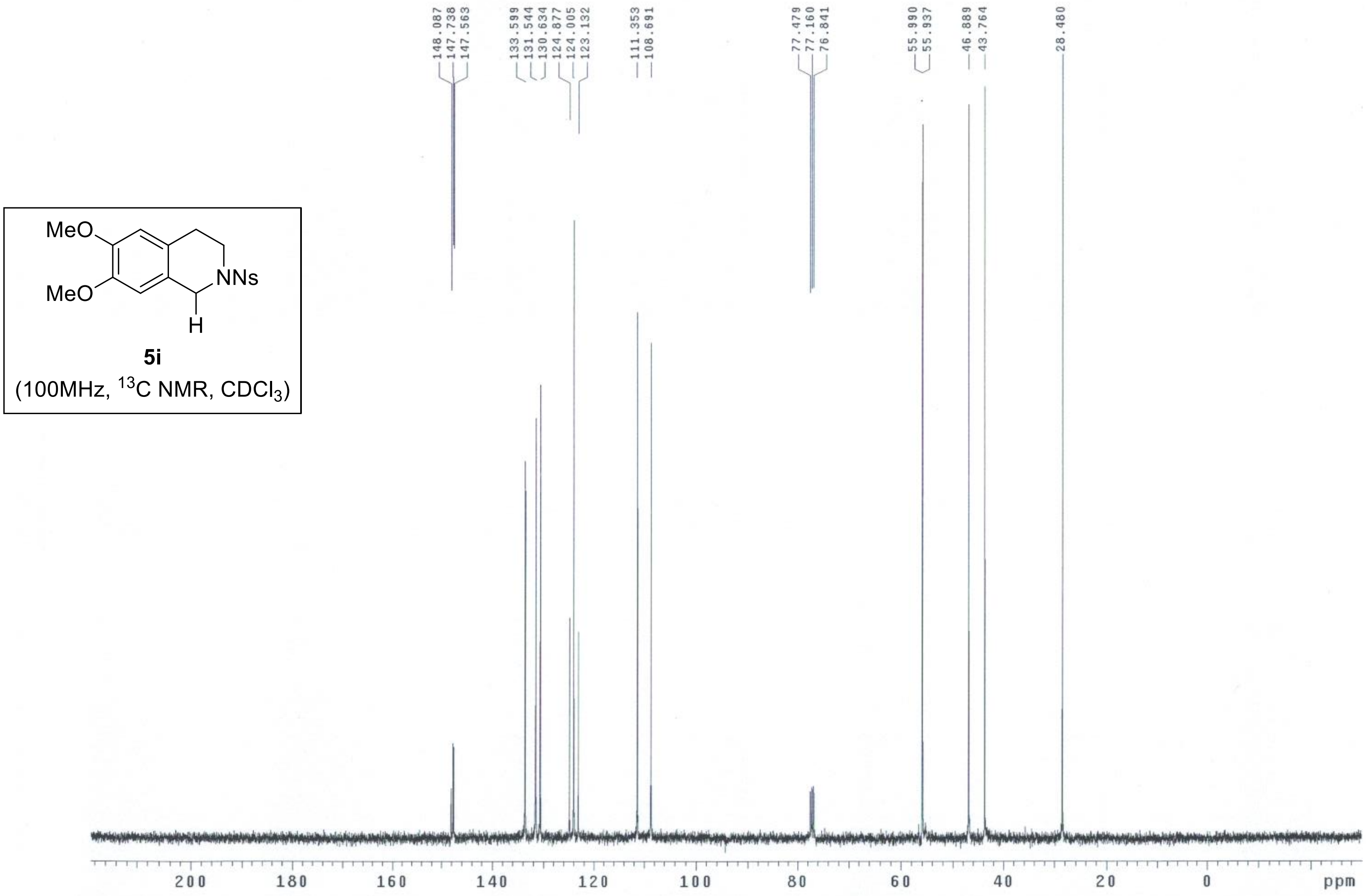

5i

(100MHz, ^{13}C NMR, $CDCl_3$)

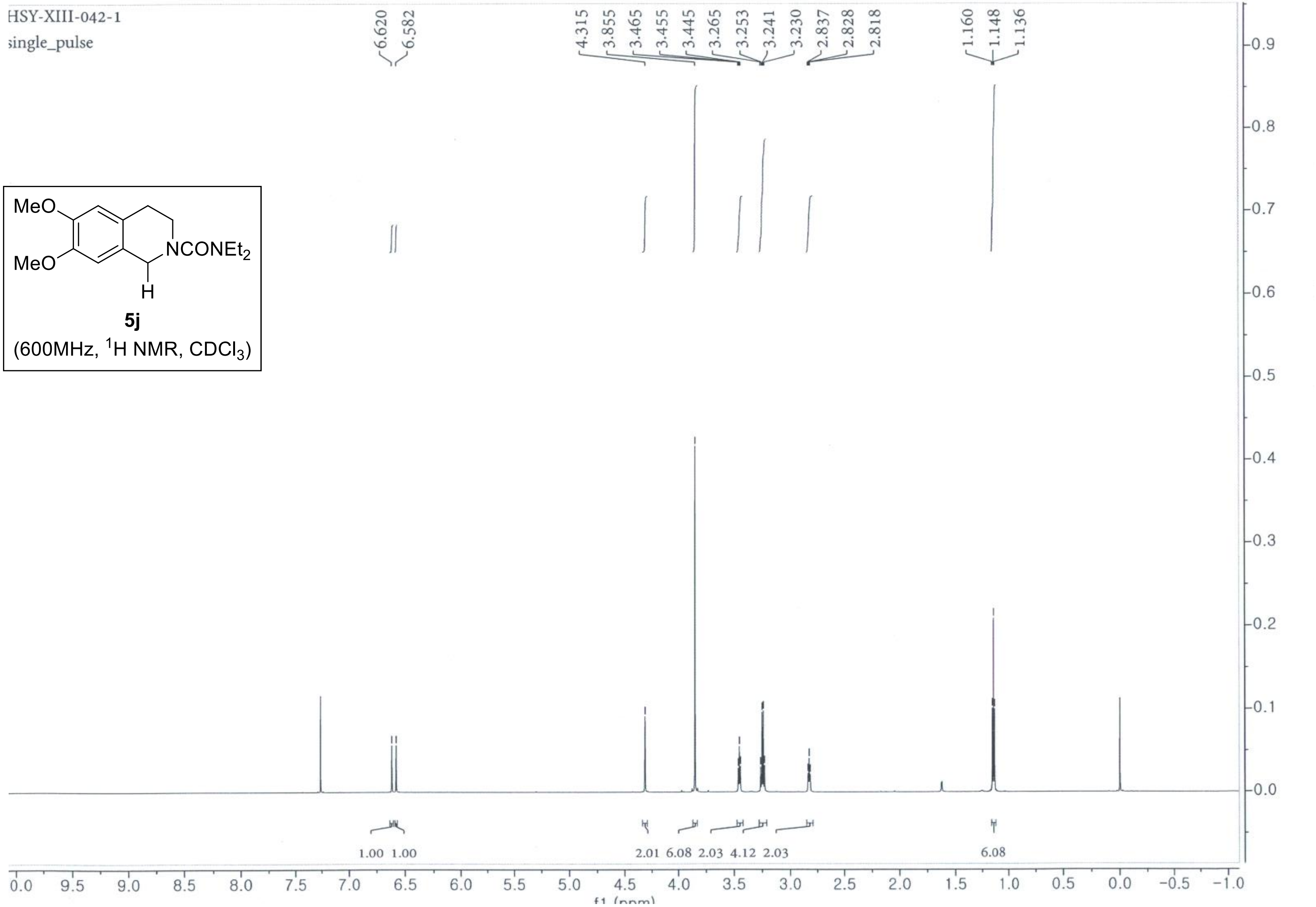
HSY-XIII-042-1
single_pulse
MeO
MeO
NCONEt2
H
5j
(600MHz, 1H NMR, CDCl3)
f1 (ppm)

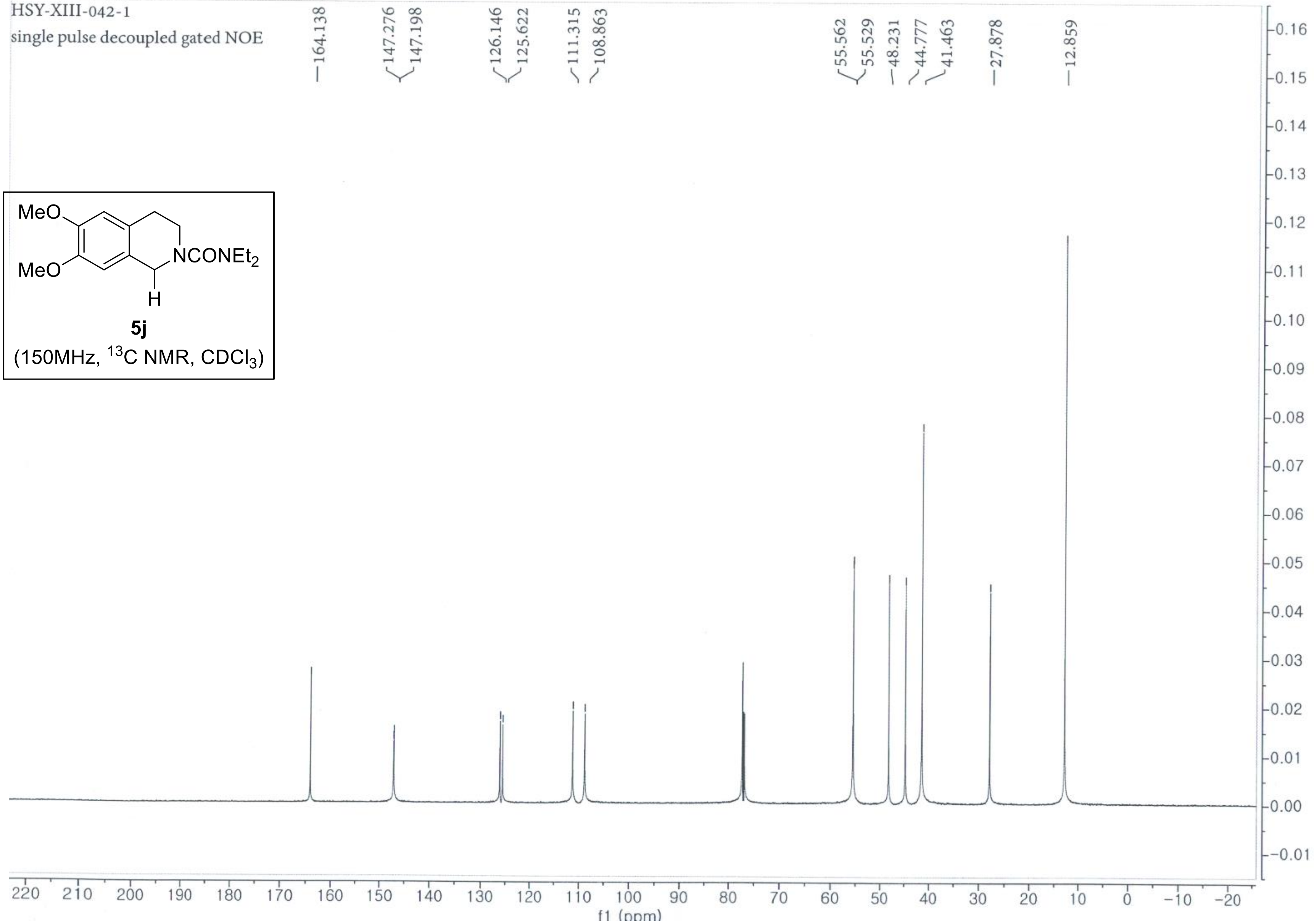

5j

(150MHz, ^{13}C NMR, $CDCl_3$)

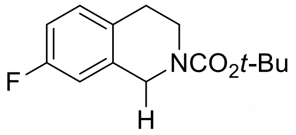

5p

(400MHz, ^{1}H NMR, $CDCl_3$)

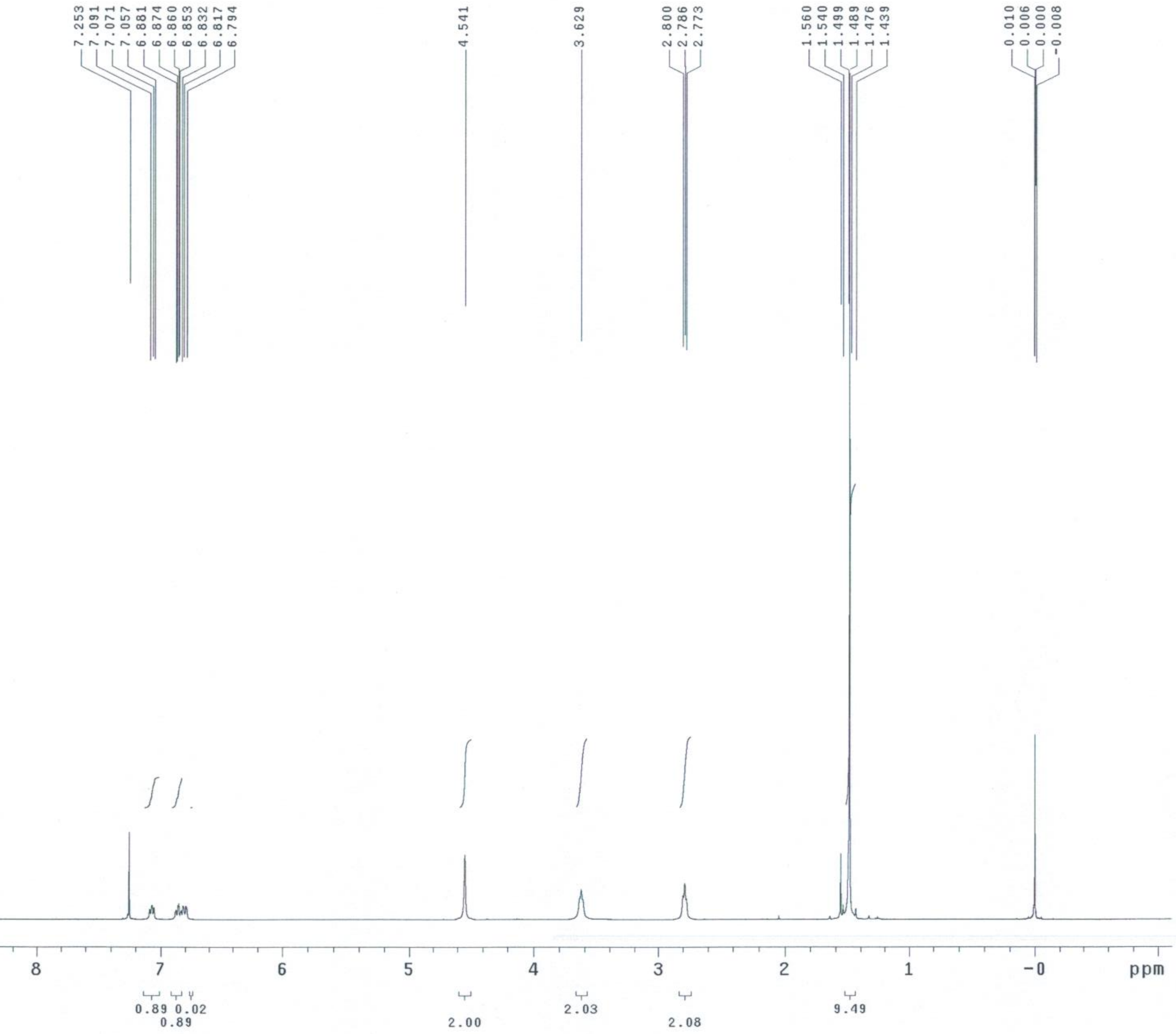

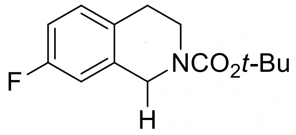

5p
(100MHz, ^{13}C NMR, $CDCl_3$)

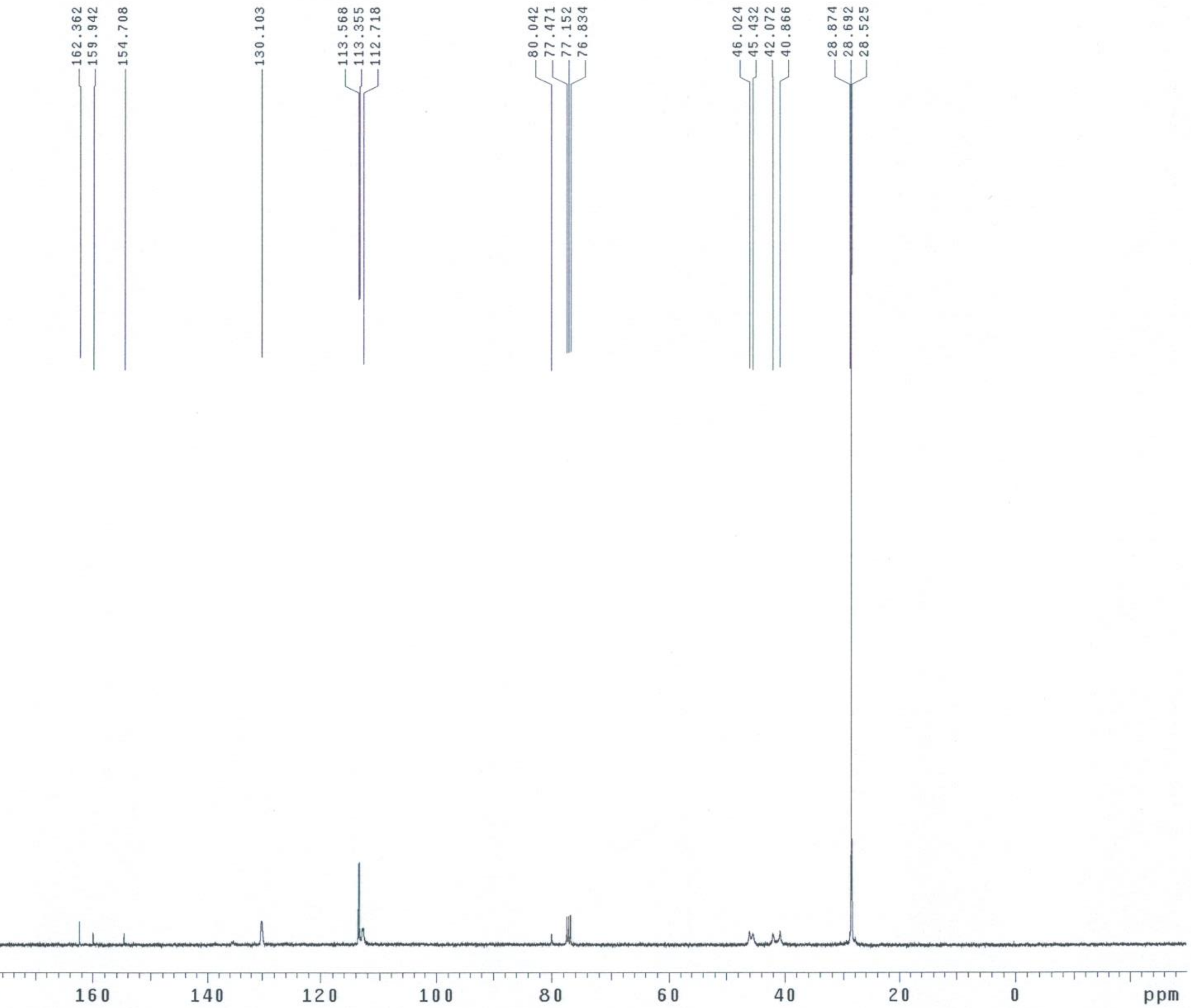

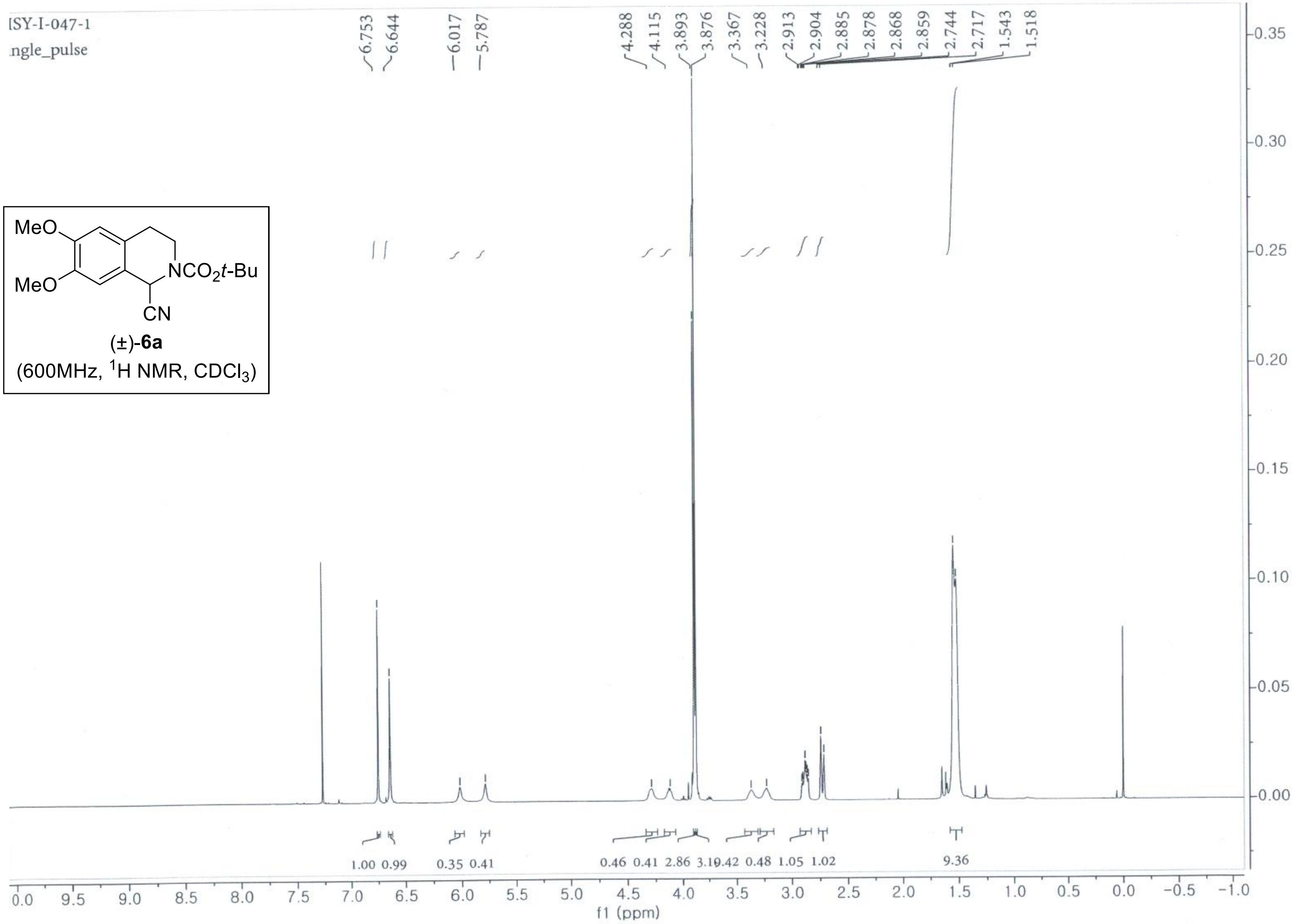

SY-I-047-1
ngle_pulse
6.753
6.644
6.017
5.787
4.288
4.115
3.893
3.876
3.367
3.228
2.913
2.904
2.885
2.878
2.868
2.859
2.744
2.717
1.543
1.518
MeO
MeO
NCO2t-Bu
CN
(±)-6a
(600MHz, 1H NMR, CDCl3)
1.00 0.99 0.35 0.41 0.46 0.41 2.86 3.10 0.42 0.48 1.05 1.02 9.36
f1 (ppm)

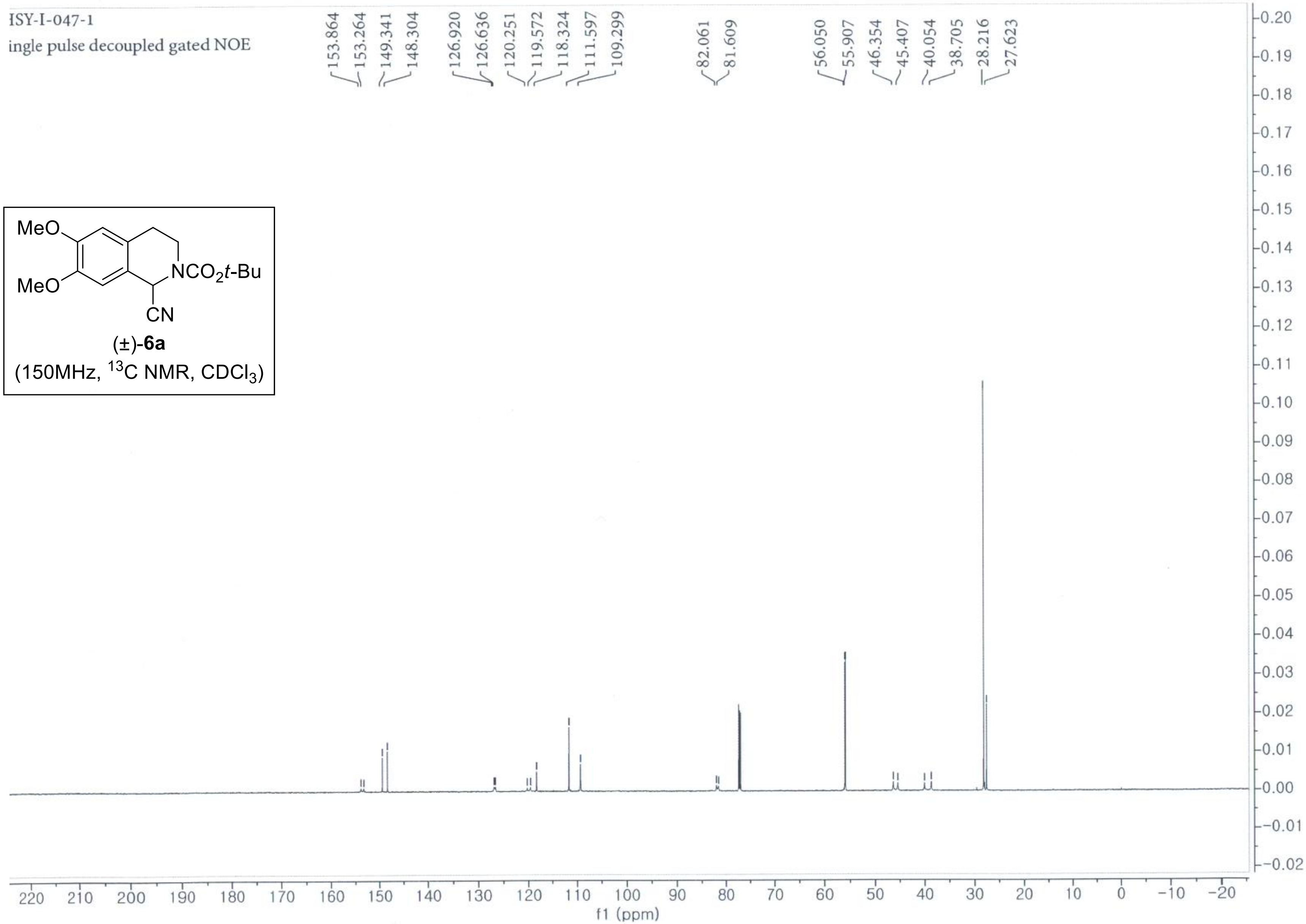

HSY-I-047-1
ingle pulse decoupled gated NOE
153.864
153.264
149.341
148.304
126.920
126.636
120.251
119.572
118.324
111.597
109.299
82.061
81.609
56.050
55.907
46.354
45.407
40.054
38.705
28.216
27.623
MeO
MeO
NCO2t-Bu
CN
(±)-6a
(150MHz, 13C NMR, CDCl3)
f1 (ppm)

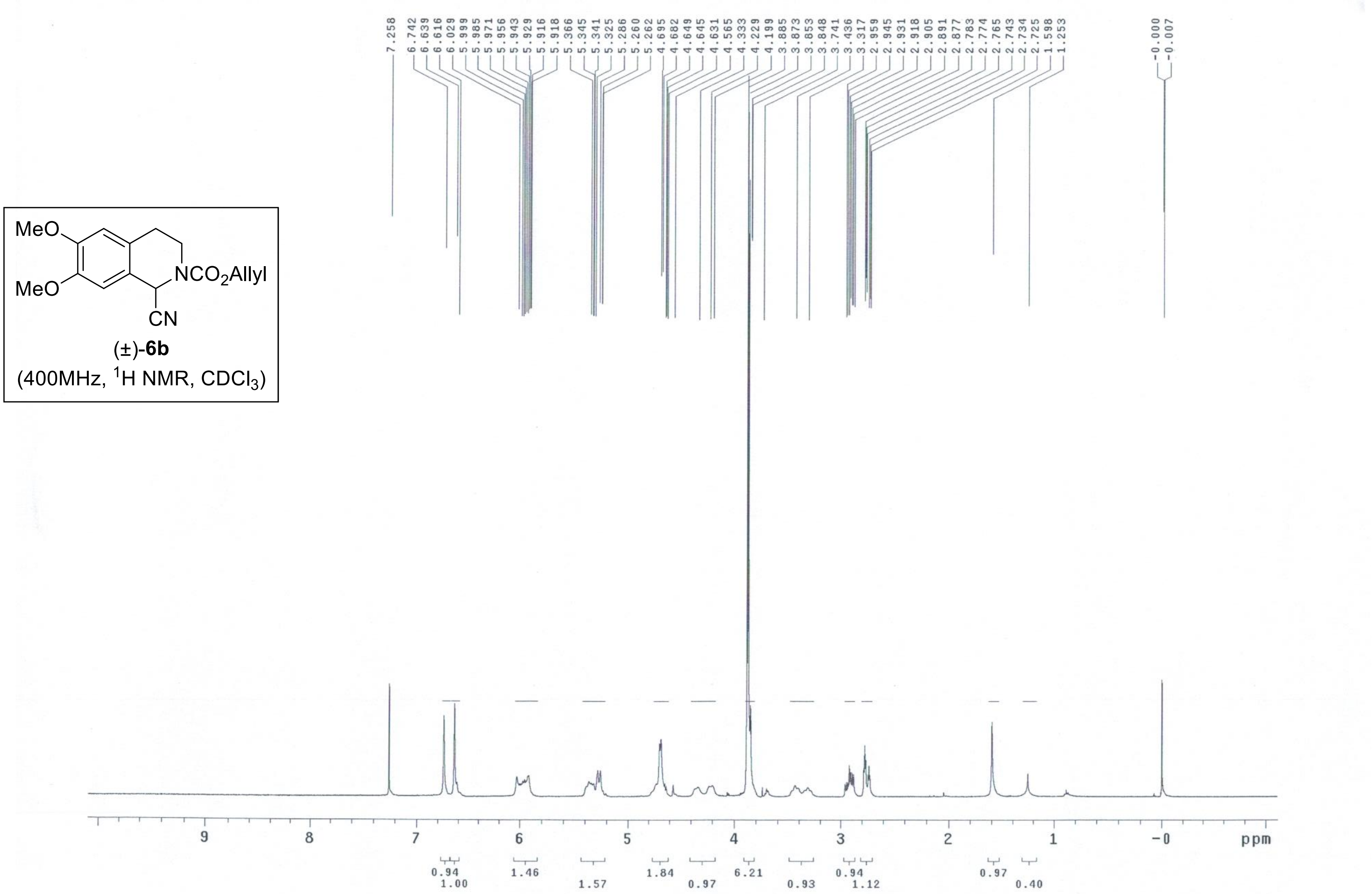
MeO
MeO
NCO2Allyl
CN
(±)-6b
(400MHz, 1H NMR, CDCl3)
ppm
0.94
1.00
1.46
1.57
1.84
0.97
6.21
0.93
0.94
1.12
0.97
0.40

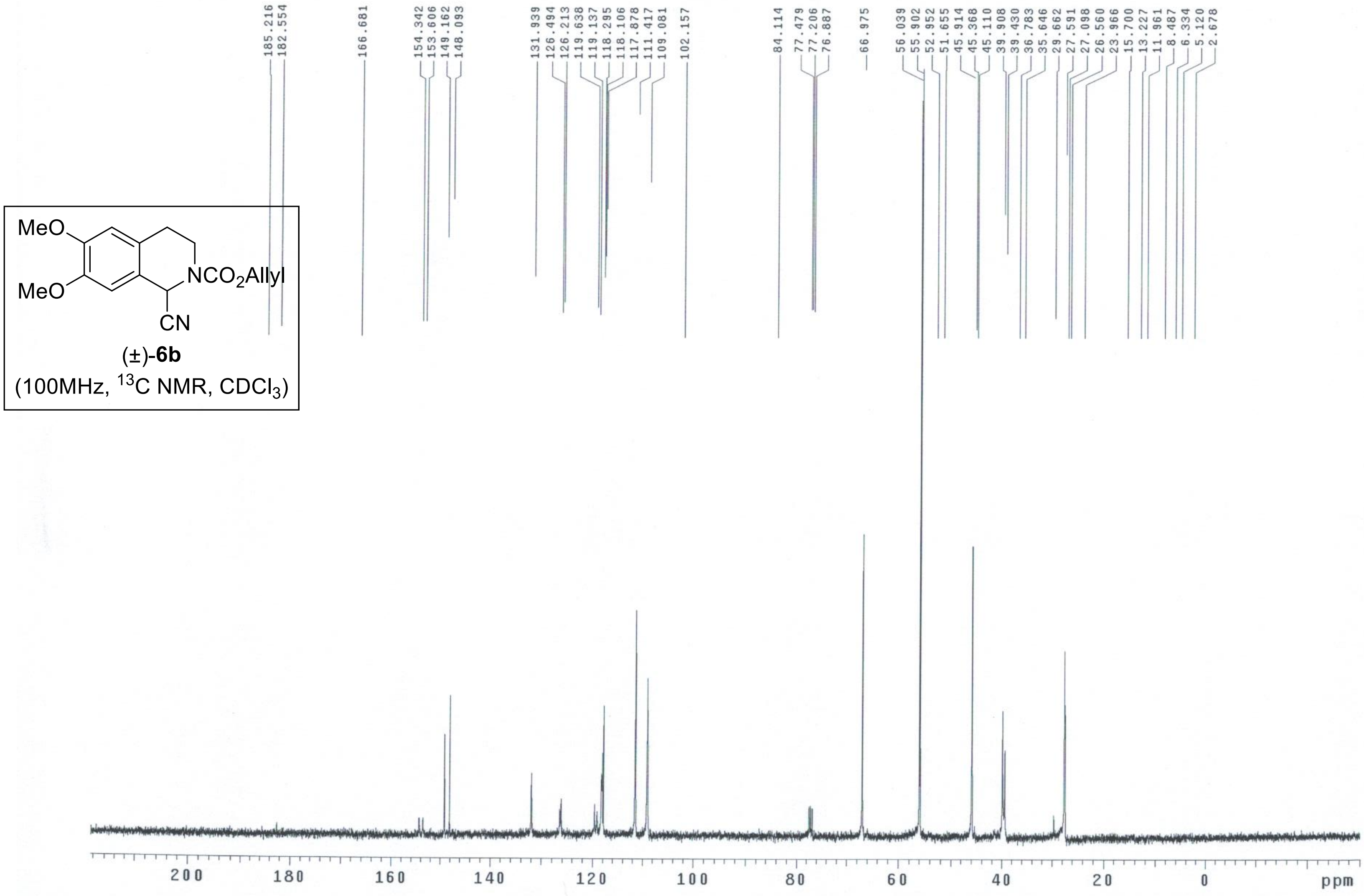
185.216
182.554
166.681
154.342
153.606
149.162
148.093
131.939
126.494
126.213
119.638
119.137
118.295
118.106
117.878
111.417
109.081
102.157
84.114
77.479
77.206
76.887
66.975
56.039
55.902
52.952
51.655
45.914
45.368
45.110
39.908
39.430
36.783
35.646
29.662
27.591
27.098
26.560
23.966
15.700
13.227
11.961
8.487
6.334
5.120
2.678
MeO
MeO
NCO$_2$Allyl
CN
(±)-**6b**
(100MHz, ^{13}C NMR, $CDCl_3$)
200
180
160
140
120
100
80
60
40
20
0
ppm

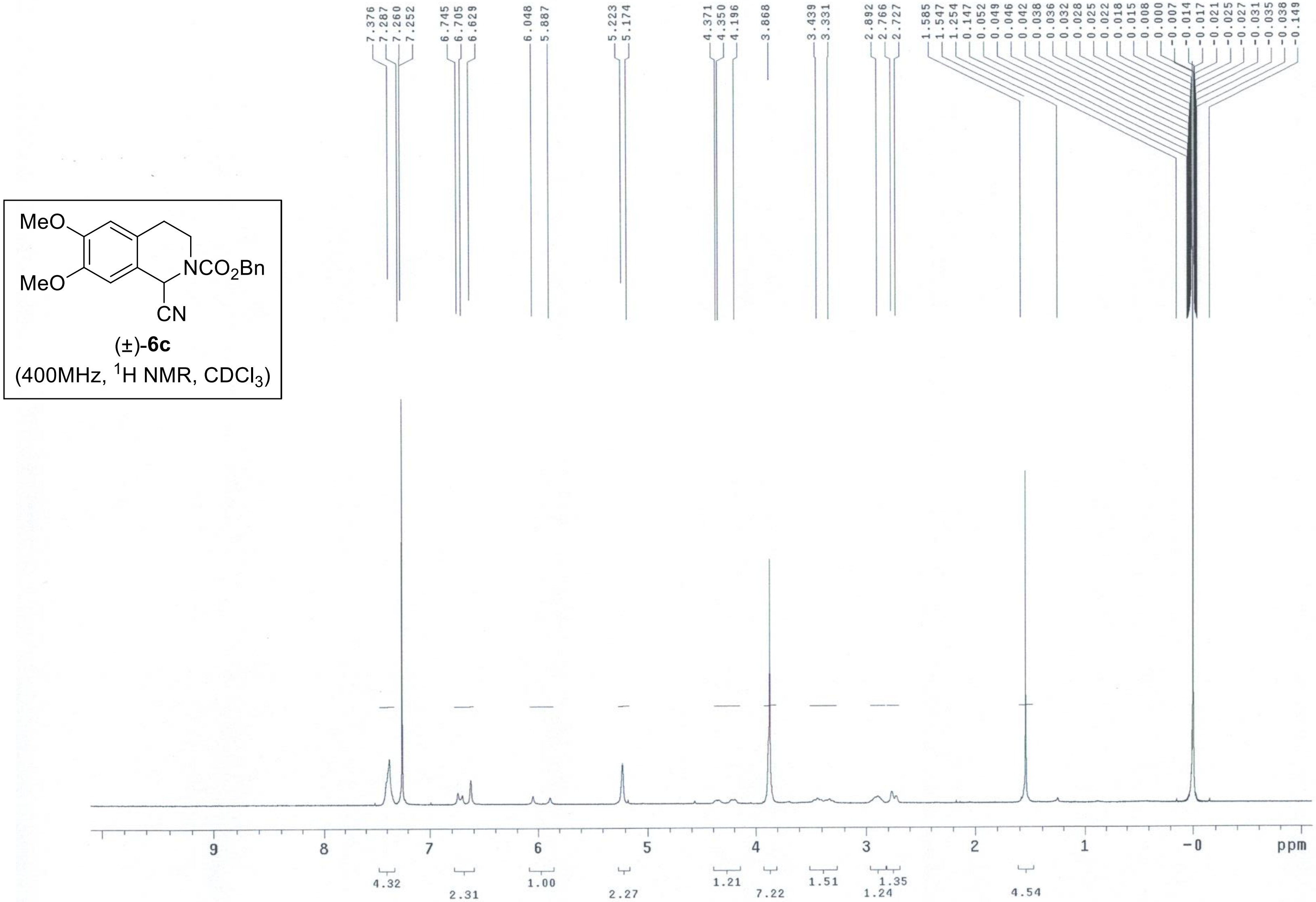

MeO
MeO
NCO2Bn
CN
(±)-6c
(400MHz, 1H NMR, CDCl3)
7.376
7.287
7.260
7.252
6.745
6.705
6.629
6.048
5.887
5.223
5.174
4.371
4.350
4.196
3.868
3.439
3.331
2.892
2.766
2.727
1.585
1.547
1.254
0.147
0.052
0.049
0.046
0.042
0.038
0.036
0.032
0.028
0.025
0.022
0.018
0.015
0.008
0.000
-0.007
-0.014
-0.017
-0.021
-0.025
-0.027
-0.031
-0.035
-0.038
-0.149
9
8
7
6
5
4
3
2
1
-0
ppm
4.32
2.31
1.00
2.27
1.21
7.22
1.51
1.24
1.35
4.54

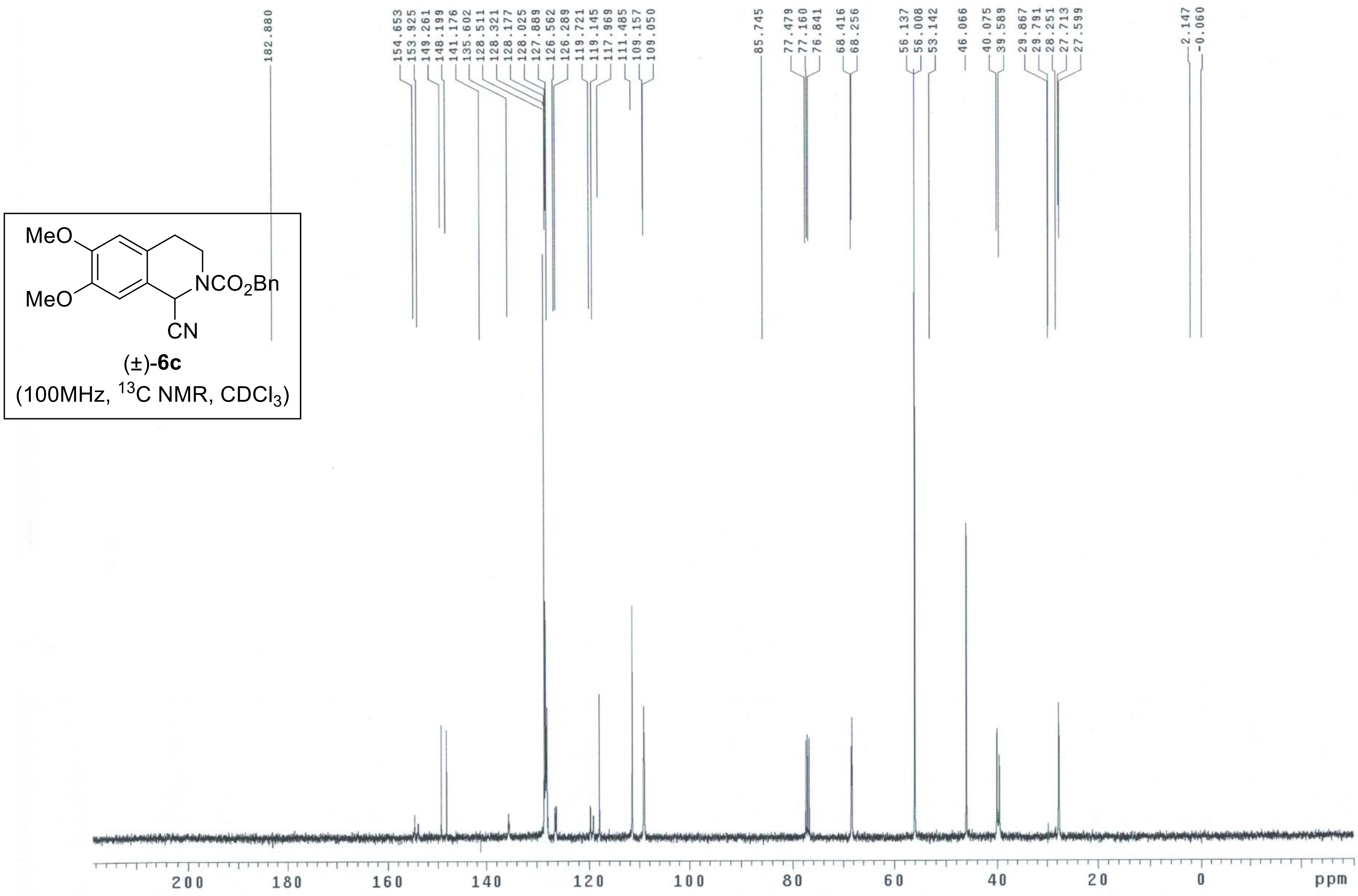
182.880
154.653
153.925
149.261
148.199
141.176
135.602
128.511
128.321
128.177
128.025
127.889
126.562
126.289
119.721
119.145
117.969
111.485
109.157
109.050
85.745
77.479
77.160
76.841
68.416
68.256
56.137
56.008
53.142
46.066
40.075
39.589
29.867
29.791
28.251
27.713
27.599
2.147
-0.060
MeO
MeO
NCO_2Bn
CN
(±)-**6c**
(100MHz, ^{13}C NMR, $CDCl_3$)
200
180
160
140
120
100
80
60
40
20
0
ppm

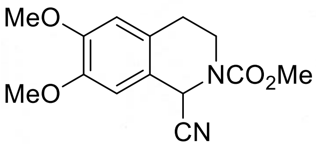

(±)-**6d**

(400MHz, ^{1}H NMR, $CDCl_3$)

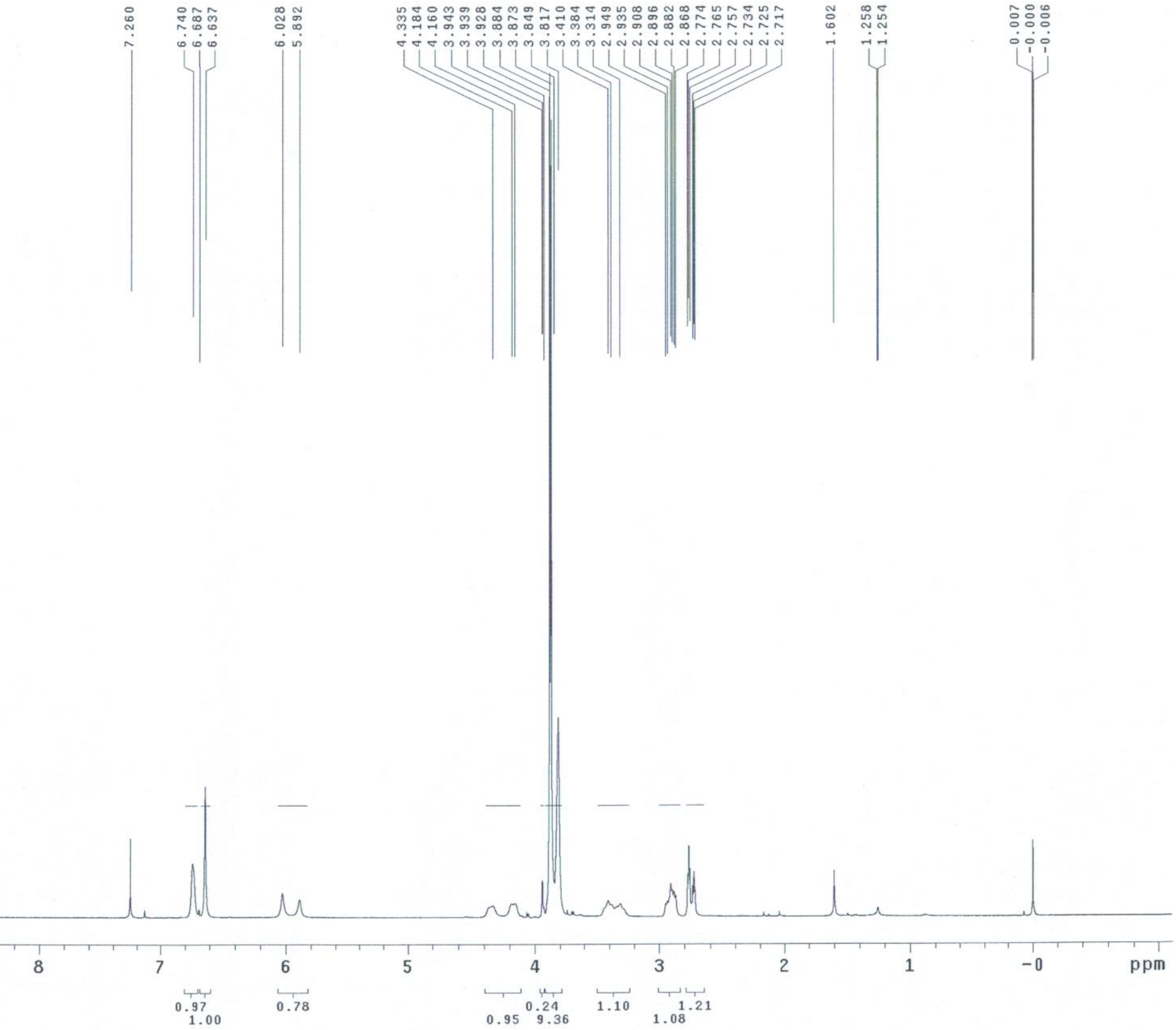

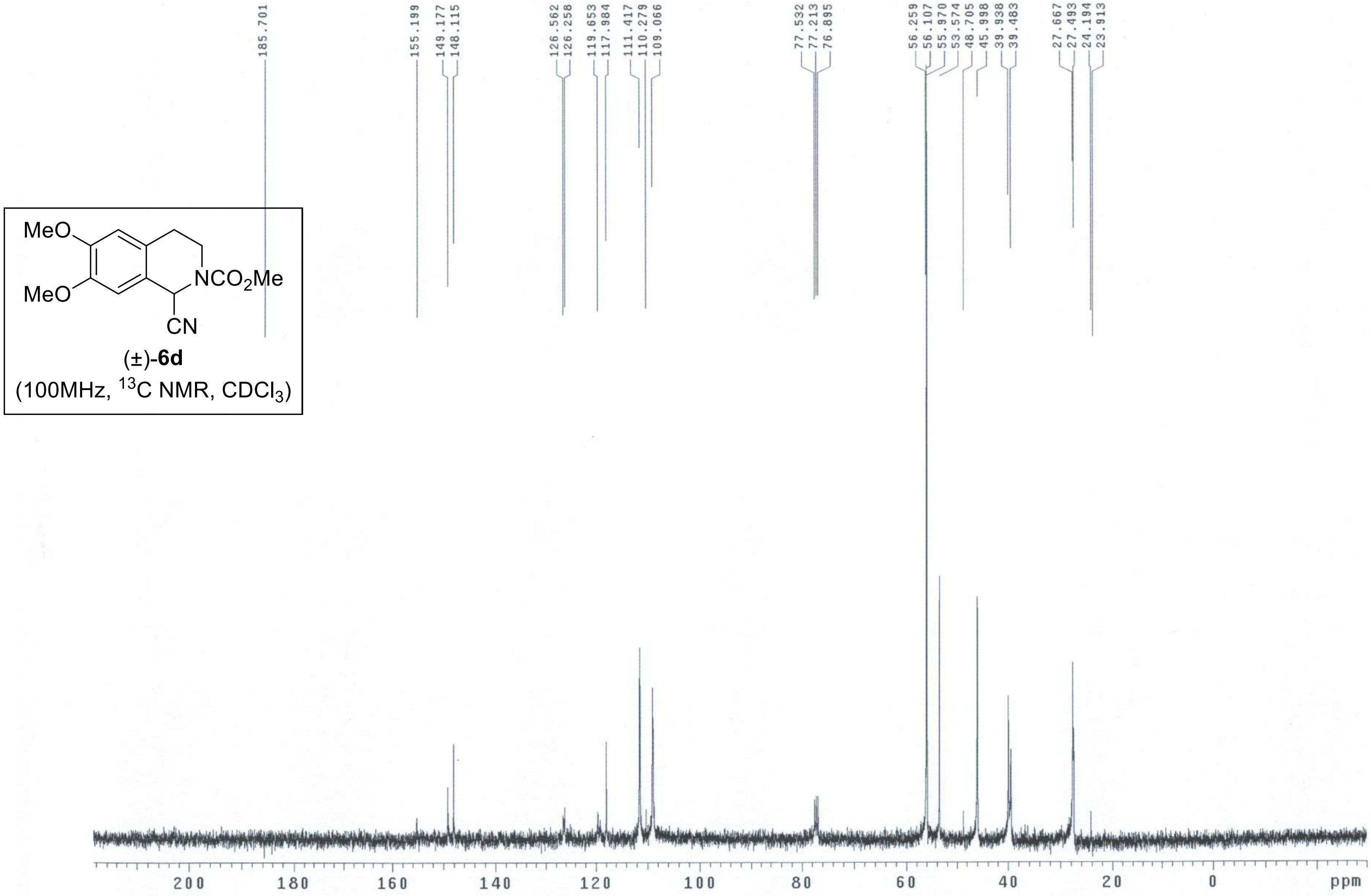
MeO
MeO
NCO2Me
CN
(±)-6d
(100MHz, 13C NMR, CDCl3)
185.701
155.199
149.177
148.115
126.562
126.258
119.653
117.984
111.417
110.279
109.066
77.532
77.213
76.895
56.259
56.107
55.970
53.574
48.705
45.998
39.938
39.483
27.667
27.493
24.194
23.913
200
180
160
140
120
100
80
60
40
20
0
ppm

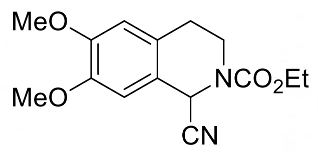

(±)-**6e**
(400MHz, ^{1}H NMR, $CDCl_3$)

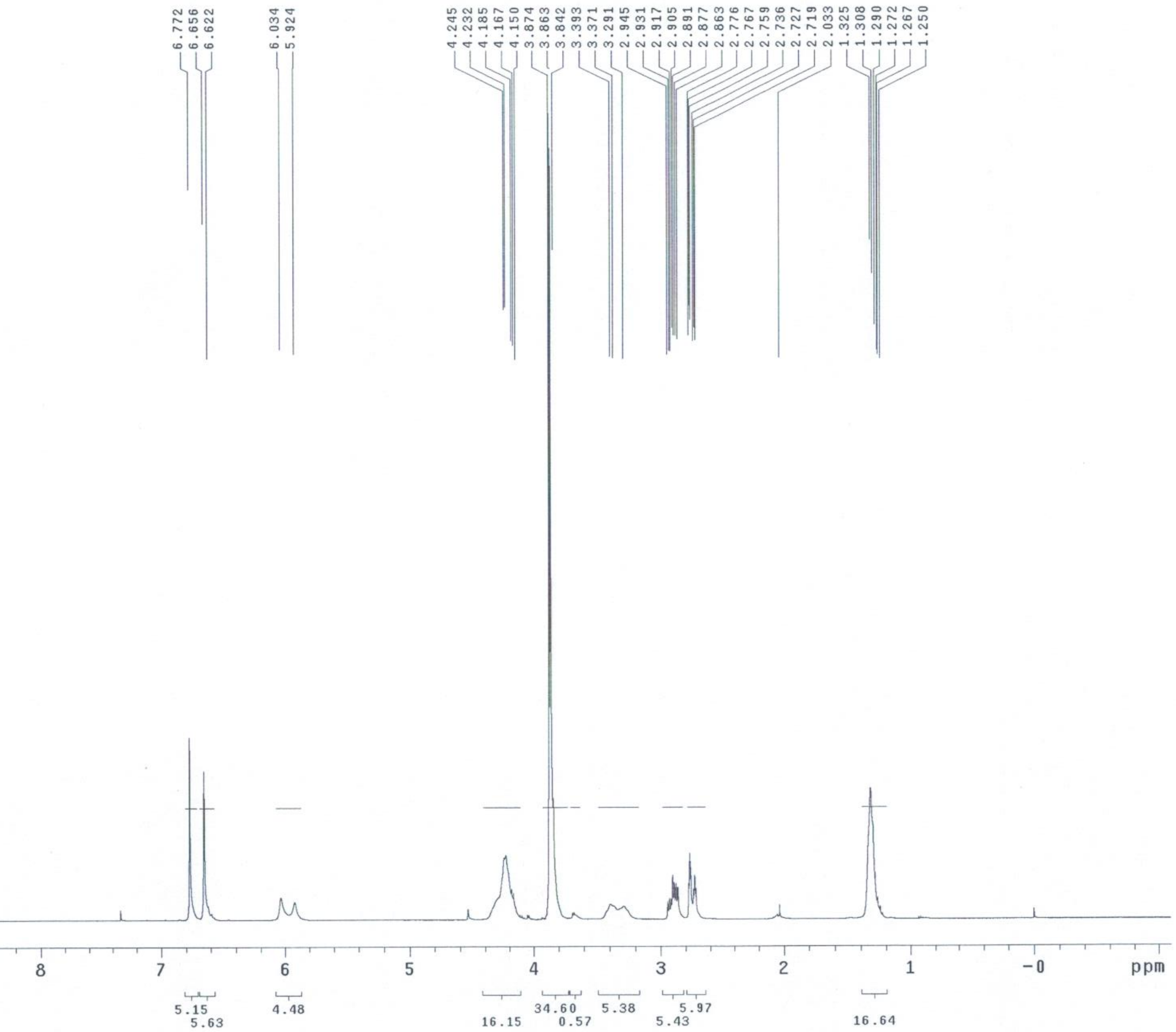

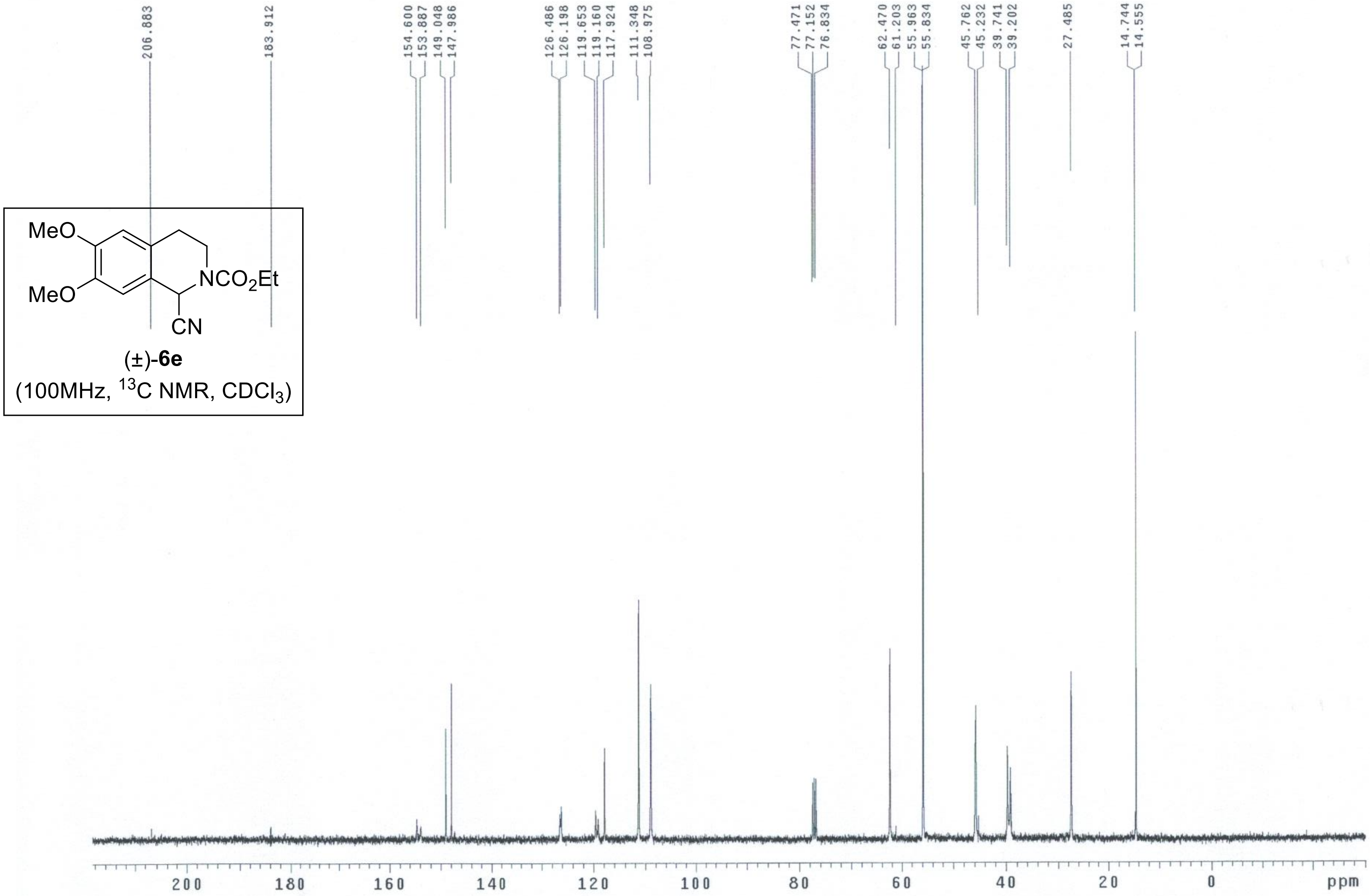

206.883
183.912
154.600
153.887
149.048
147.986
126.486
126.198
119.653
119.160
117.924
111.348
108.975
77.471
77.152
76.834
62.470
61.203
55.963
55.834
45.762
45.232
39.741
39.202
27.485
14.744
14.555
MeO
MeO
NCO2Et
CN
(±)-6e
(100MHz, 13C NMR, CDCl3)
200
180
160
140
120
100
80
60
40
20
0
ppm

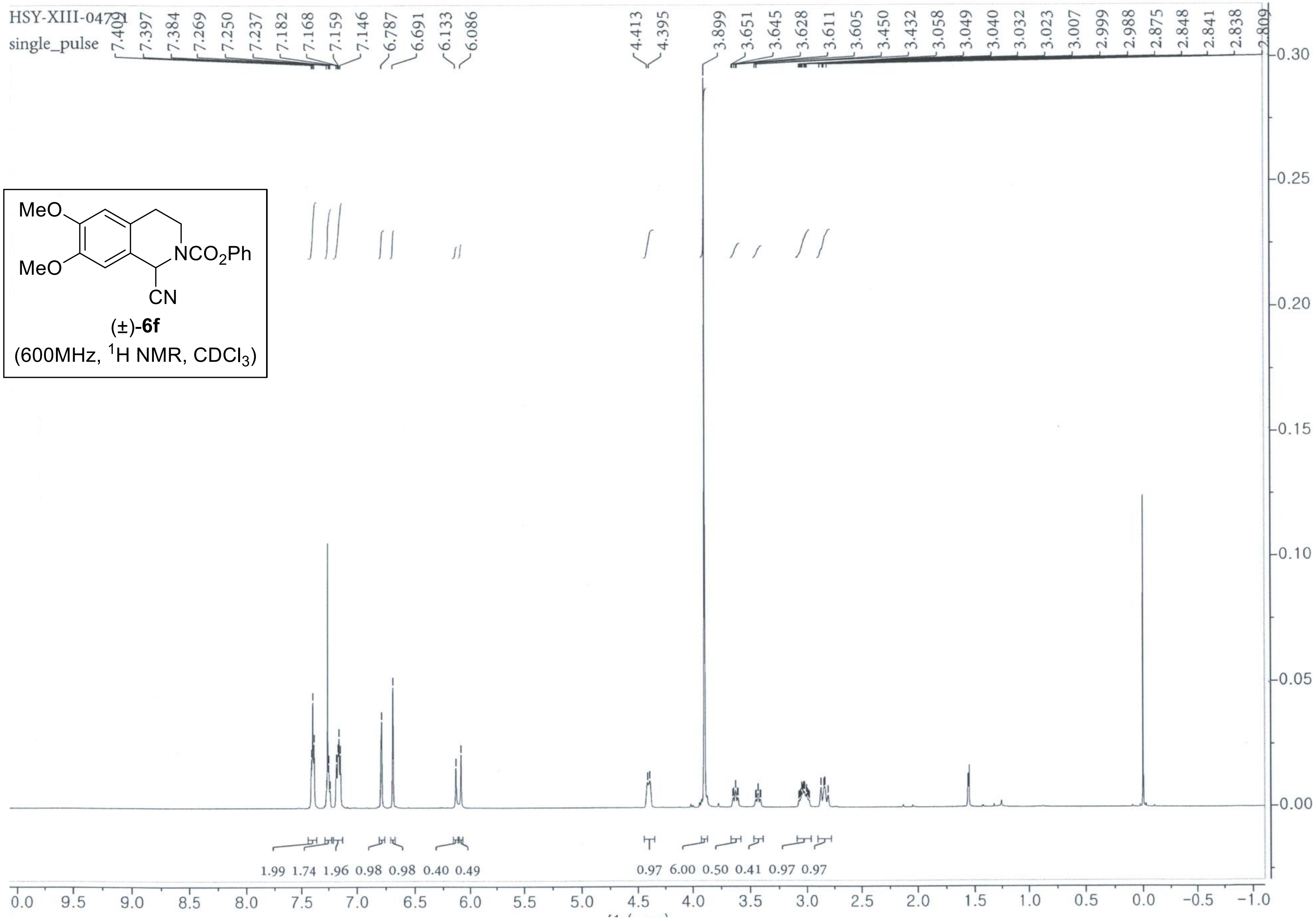
HSY-XIII-0471
single_pulse
7.401 7.397 7.384 7.269 7.250 7.237 7.182 7.168 7.159 7.146 6.787 6.691 6.133 6.086
4.413 4.395
3.899 3.651 3.645 3.628 3.611 3.605 3.450 3.432 3.058 3.049 3.040 3.032 3.023 3.007 2.999 2.988 2.875 2.848 2.841 2.838 2.809
MeO
MeO
NCO2Ph
CN
(±)-6f
(600MHz, 1H NMR, CDCl3)
1.99 1.74 1.96 0.98 0.98 0.40 0.49
0.97 6.00 0.50 0.41 0.97 0.97

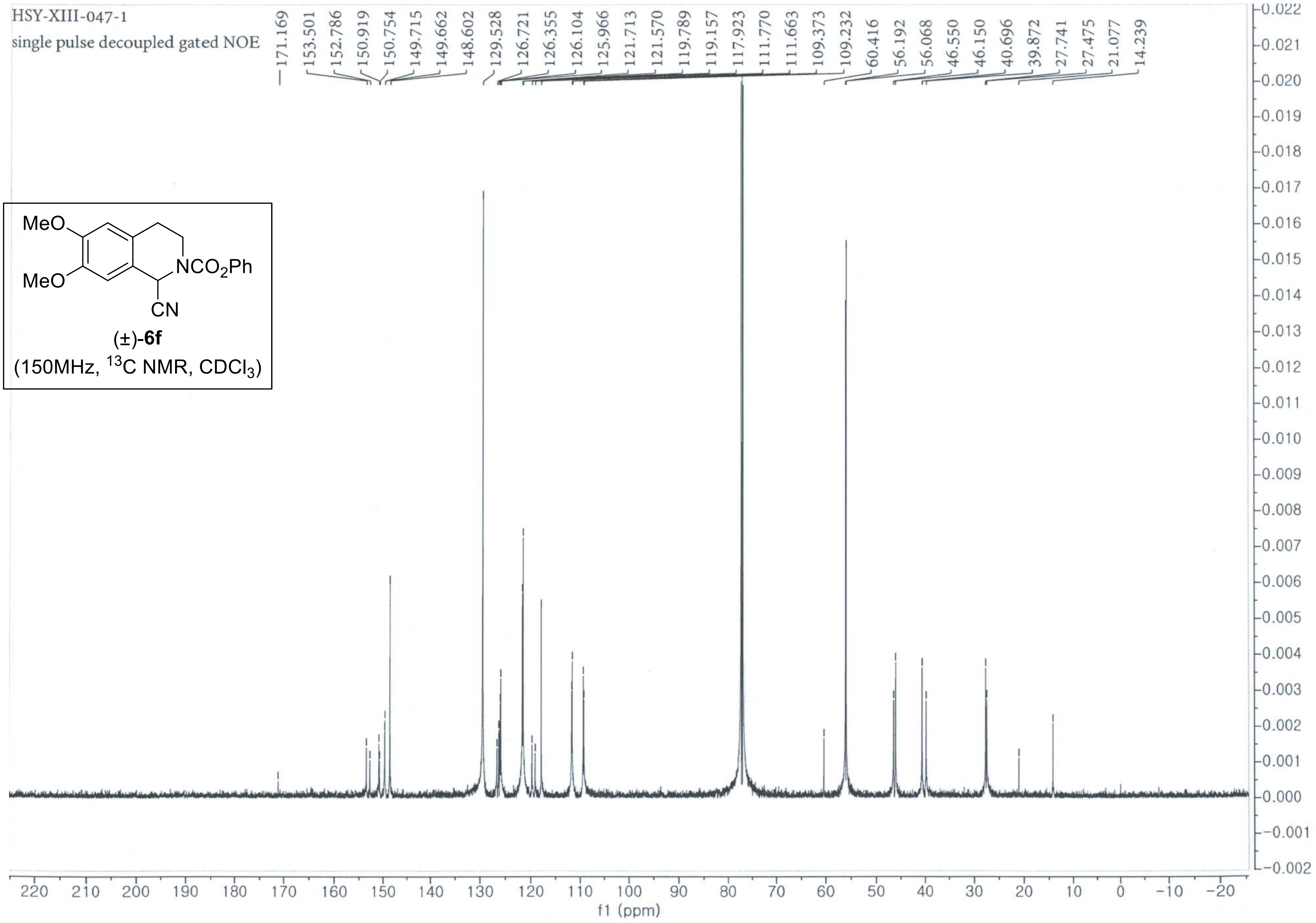
HSY-XIII-047-1
single pulse decoupled gated NOE
171.169
153.501
152.786
150.919
150.754
149.715
149.662
148.602
129.528
126.721
126.355
126.104
125.966
121.713
121.570
119.789
119.157
117.923
111.770
111.663
109.373
109.232
60.416
56.192
56.068
46.550
46.150
40.696
39.872
27.741
27.475
21.077
14.239
MeO
MeO
NCO2Ph
CN
(±)-6f
(150MHz, 13C NMR, CDCl3)
f1 (ppm)

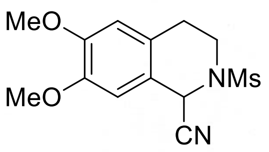

(±)-**6g**
(400MHz, ^{1}H NMR, $CDCl_3$)

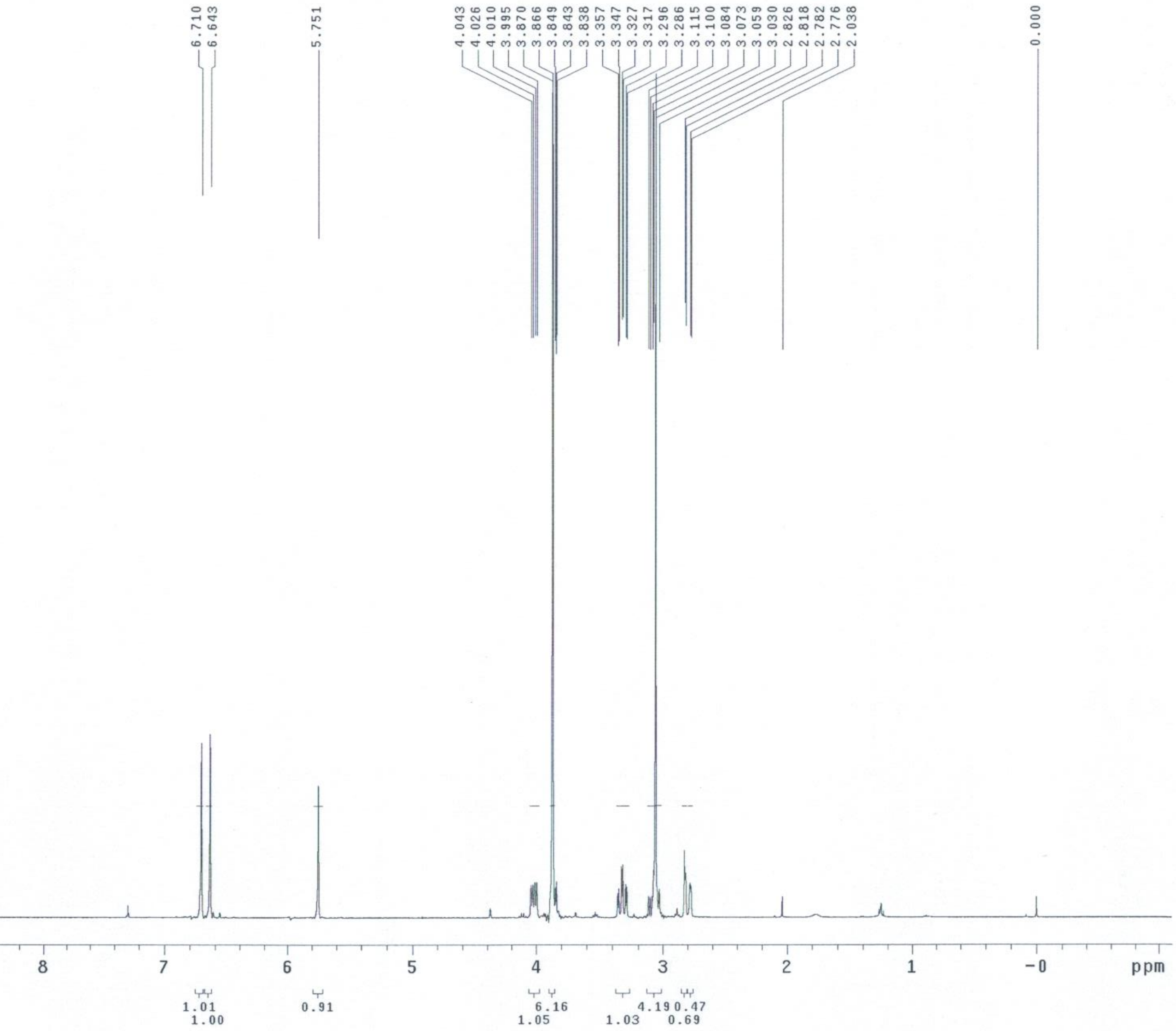

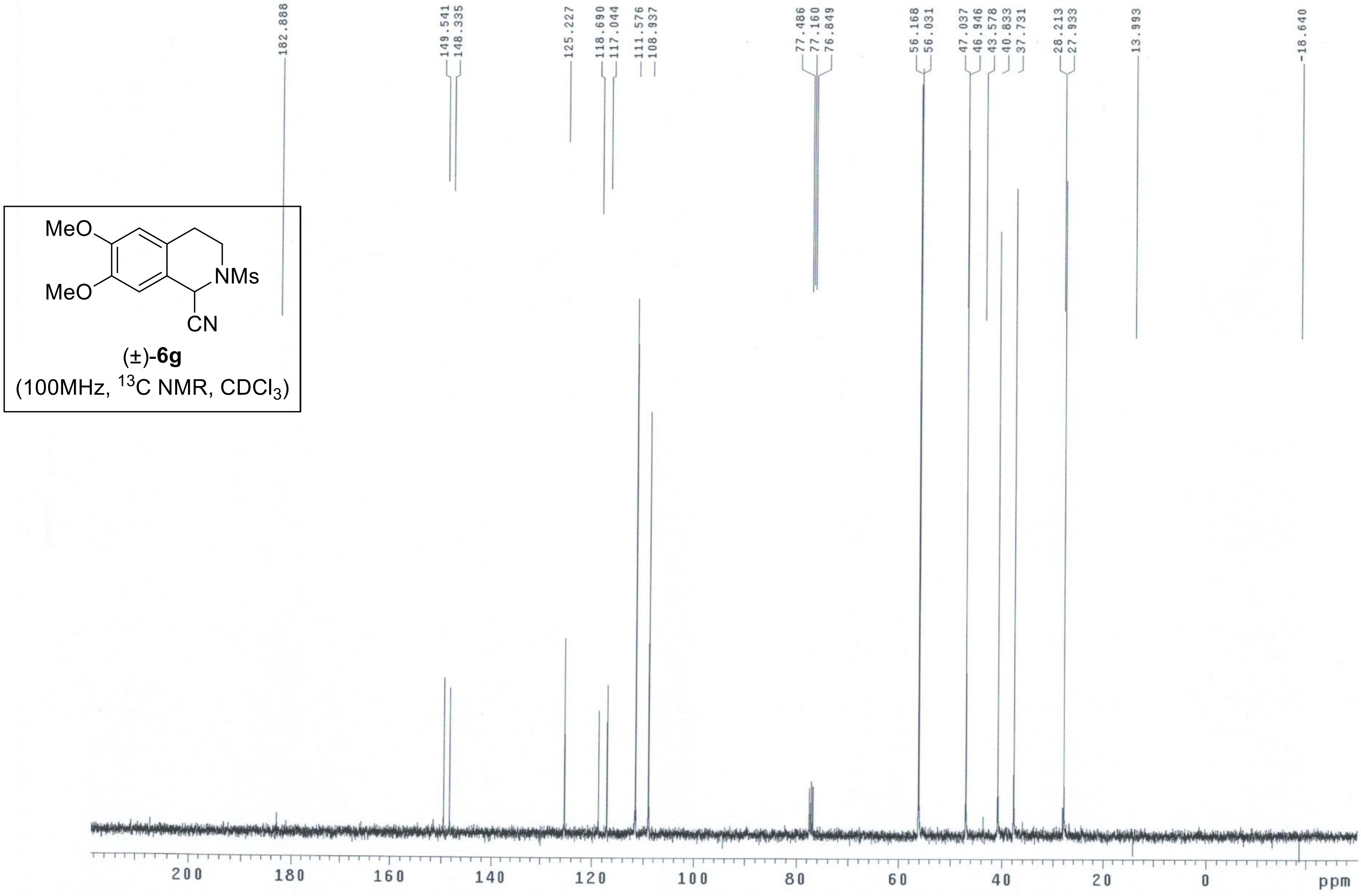
182.888
149.541
148.335
125.227
118.690
117.044
111.576
108.937
77.486
77.160
76.849
56.168
56.031
47.037
46.946
43.578
40.833
37.731
28.213
27.933
13.993
-18.640
MeO
MeO
NMs
CN
(±)-6g
(100MHz, 13C NMR, CDCl3)
200
180
160
140
120
100
80
60
40
20
0
ppm

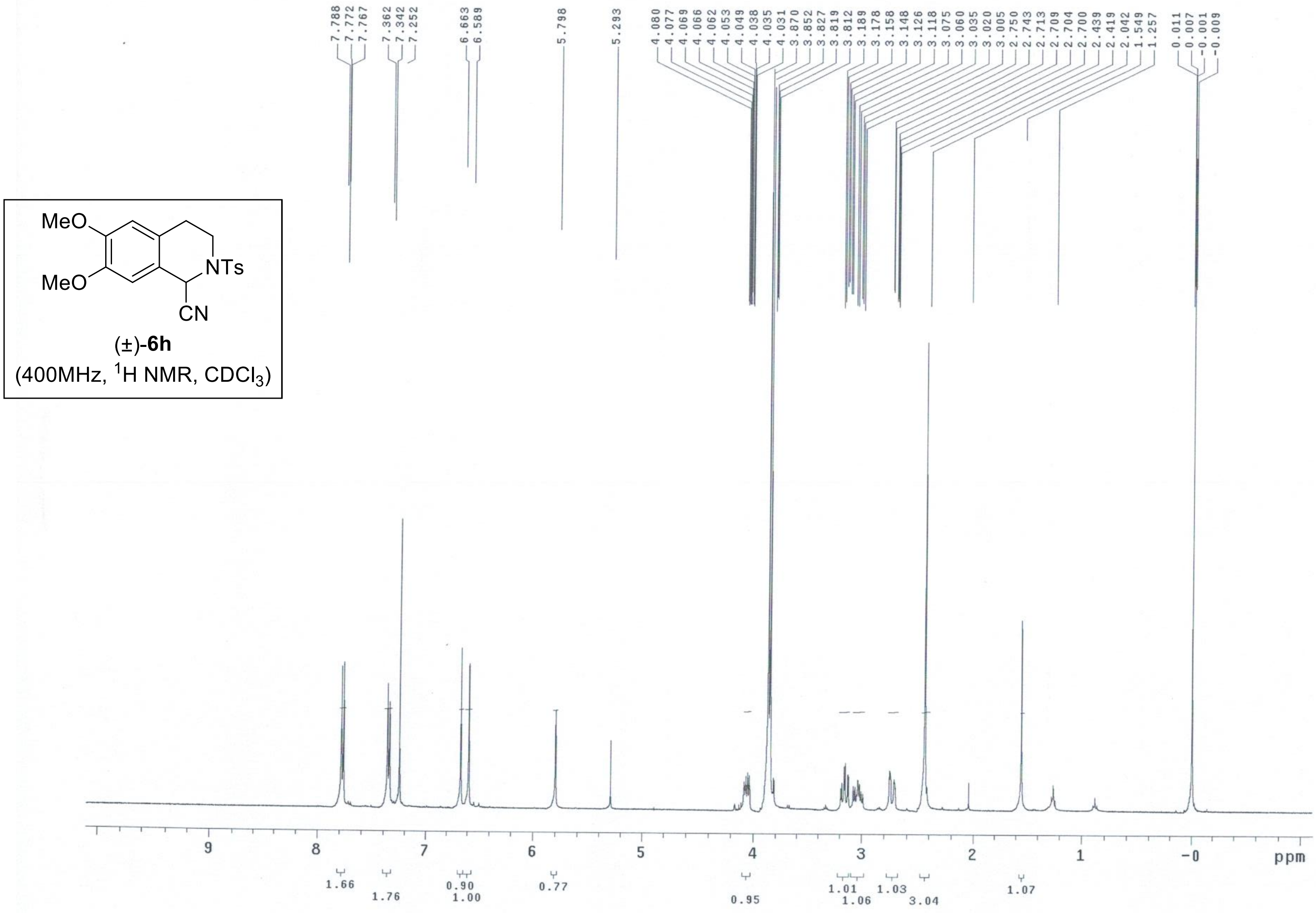

(±)-**6h**
(400MHz, ^{1}H NMR, $CDCl_3$)

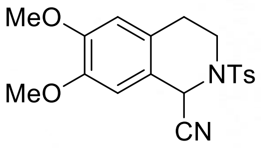

(±)-**6h**
(100MHz, ^{13}C NMR, $CDCl_3$)

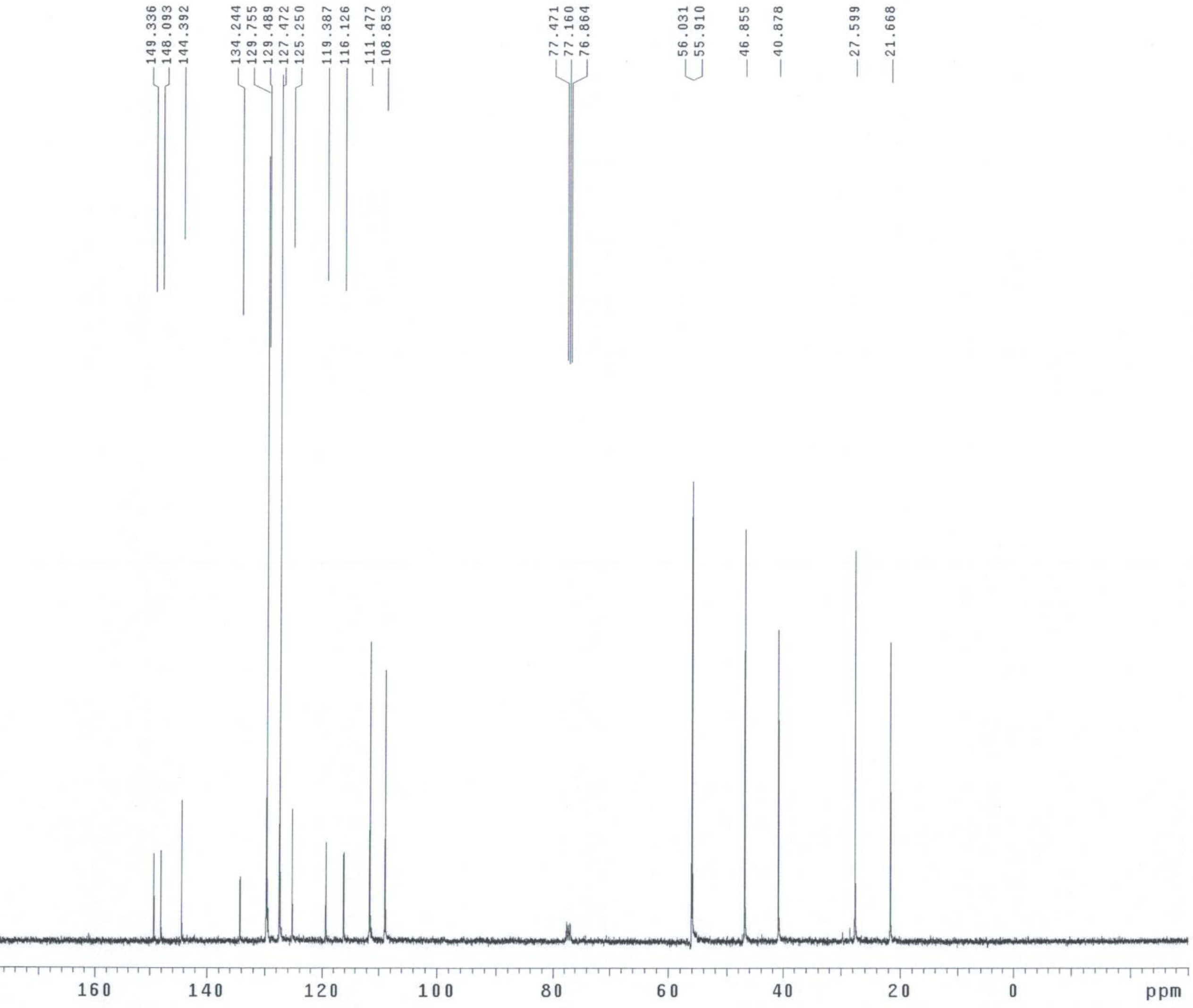

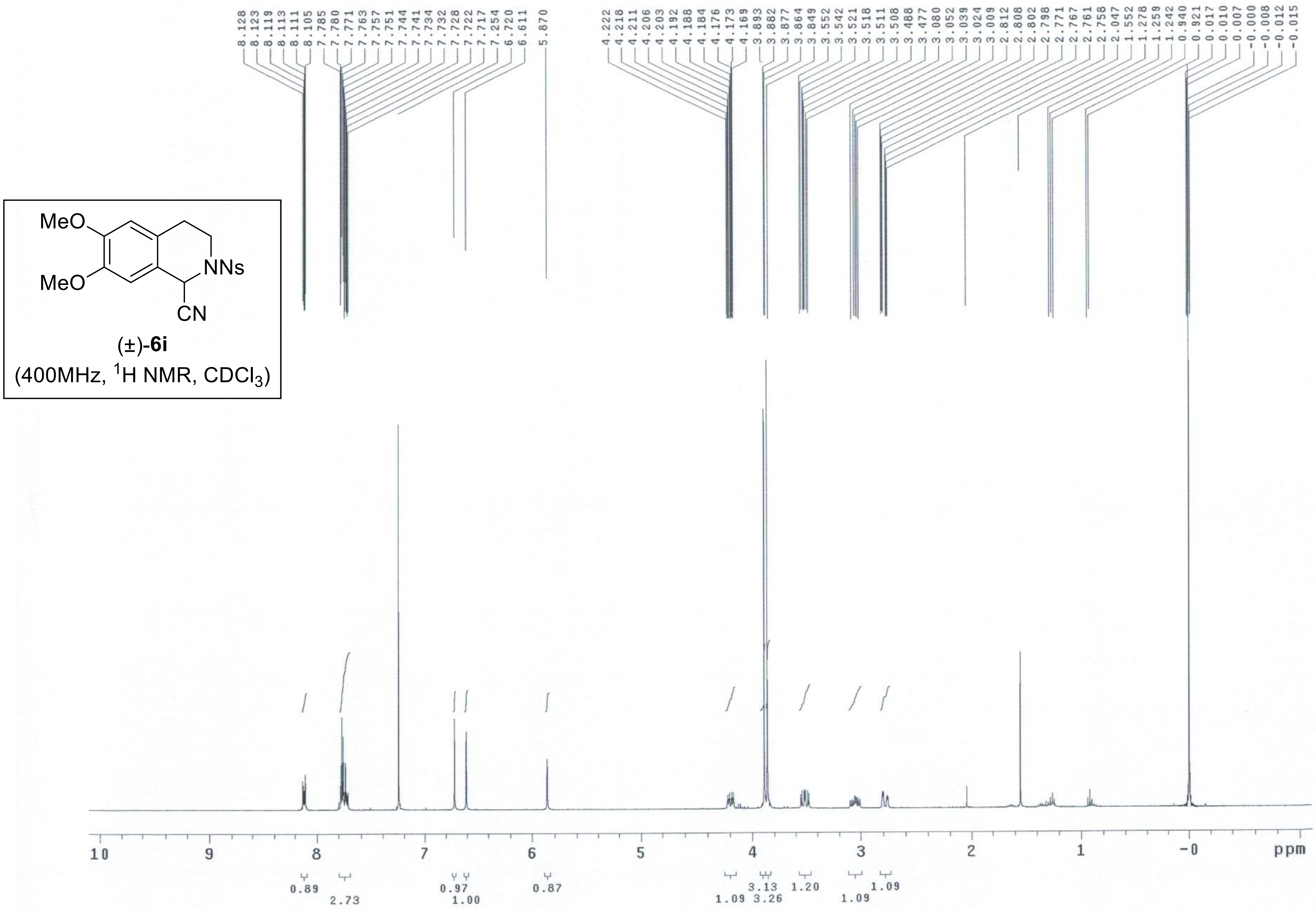
MeO
MeO
NNs
CN
(±)-6i
(400MHz, 1H NMR, CDCl3)
ppm

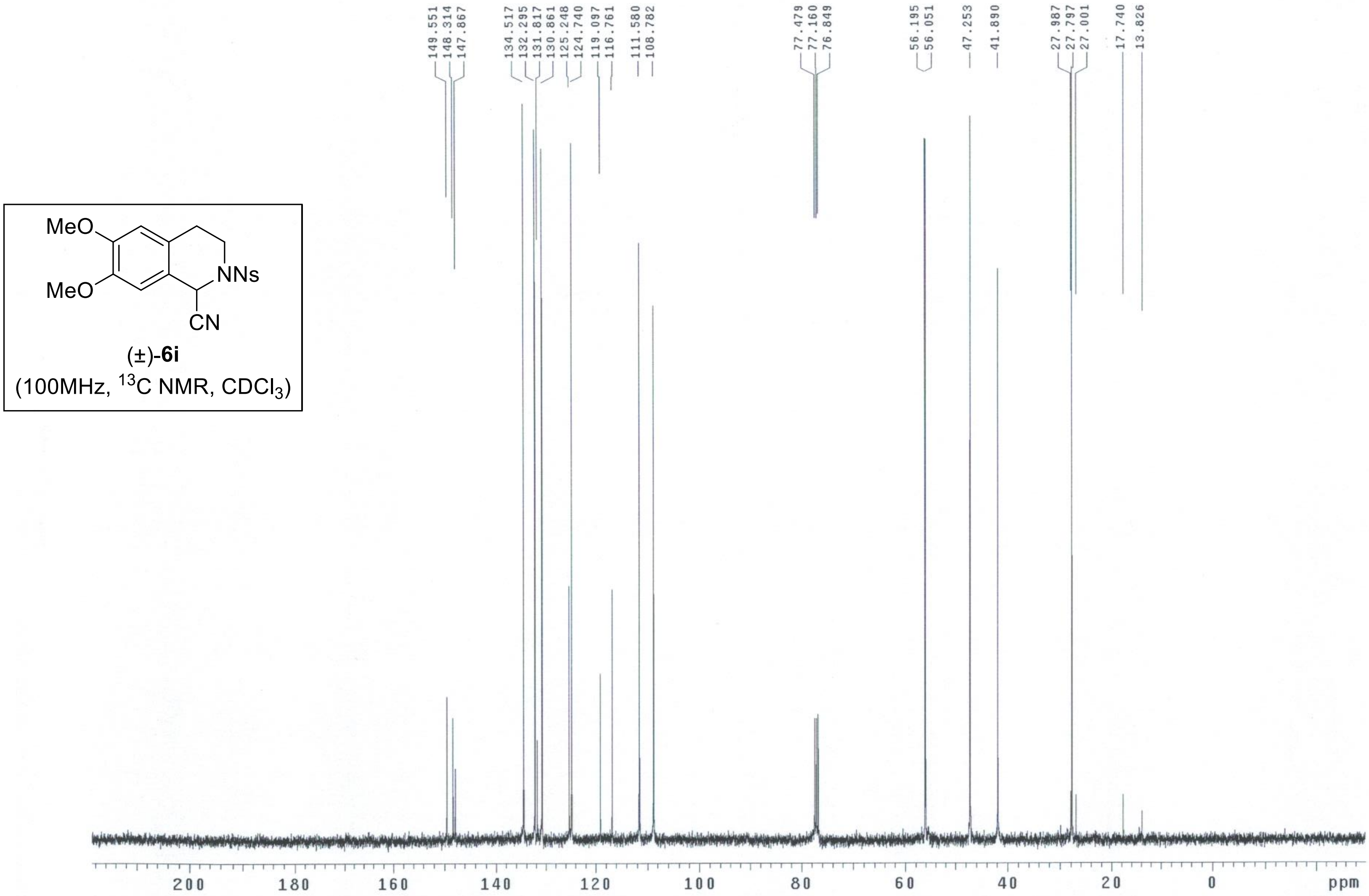

(±)-**6i**
(100MHz, ^{13}C NMR, $CDCl_3$)

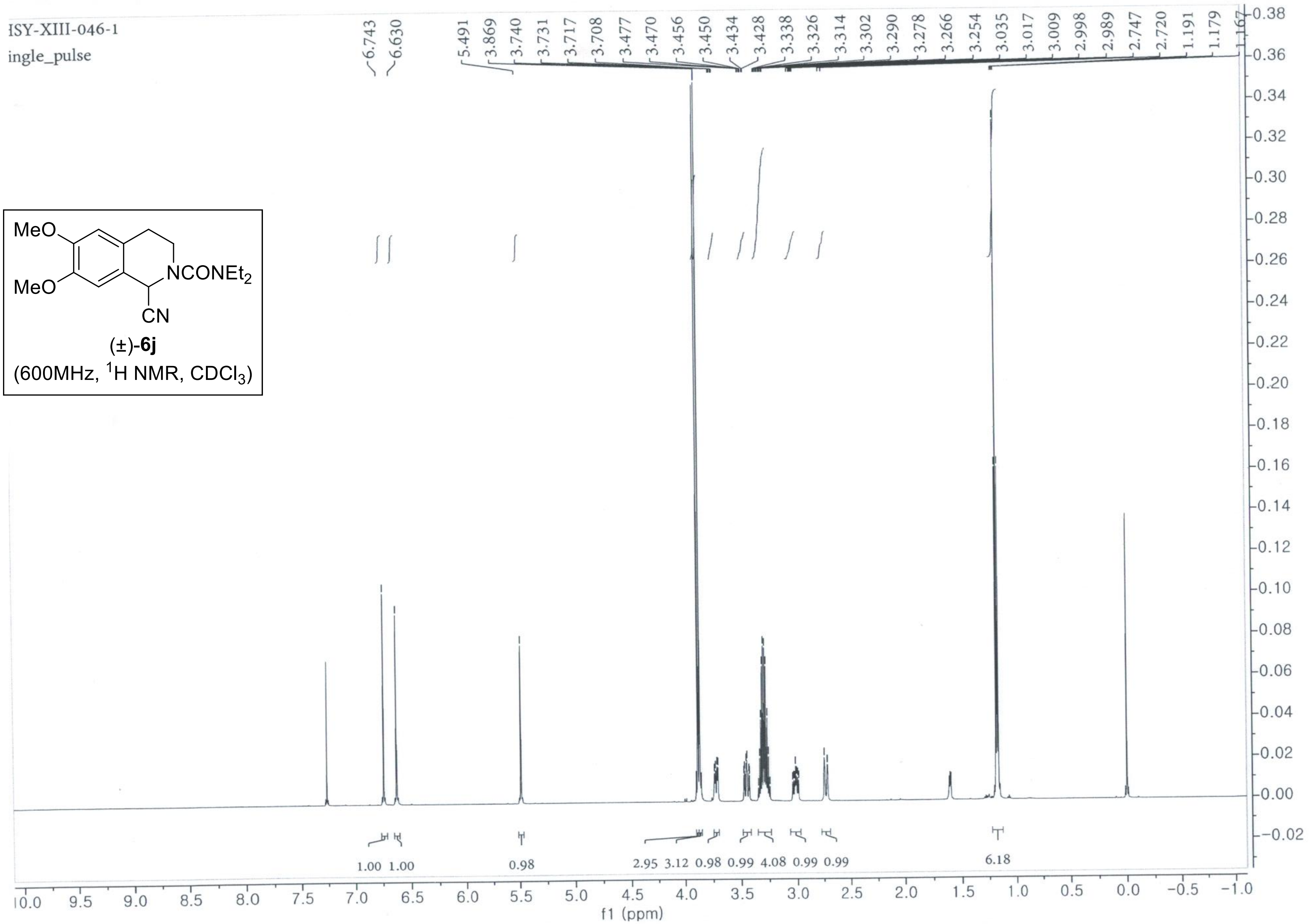

ISY-XIII-046-1
ingle_pulse
MeO
MeO
NCONEt2
CN
(±)-6j
(600MHz, 1H NMR, CDCl3)
6.743
6.630
5.491
3.869
3.740
3.731
3.717
3.708
3.477
3.470
3.456
3.450
3.434
3.428
3.338
3.326
3.314
3.302
3.290
3.278
3.266
3.254
3.035
3.017
3.009
2.998
2.989
2.747
2.720
1.191
1.179
1.167
1.00 1.00 0.98 2.95 3.12 0.98 0.99 4.08 0.99 0.99 6.18
f1 (ppm)

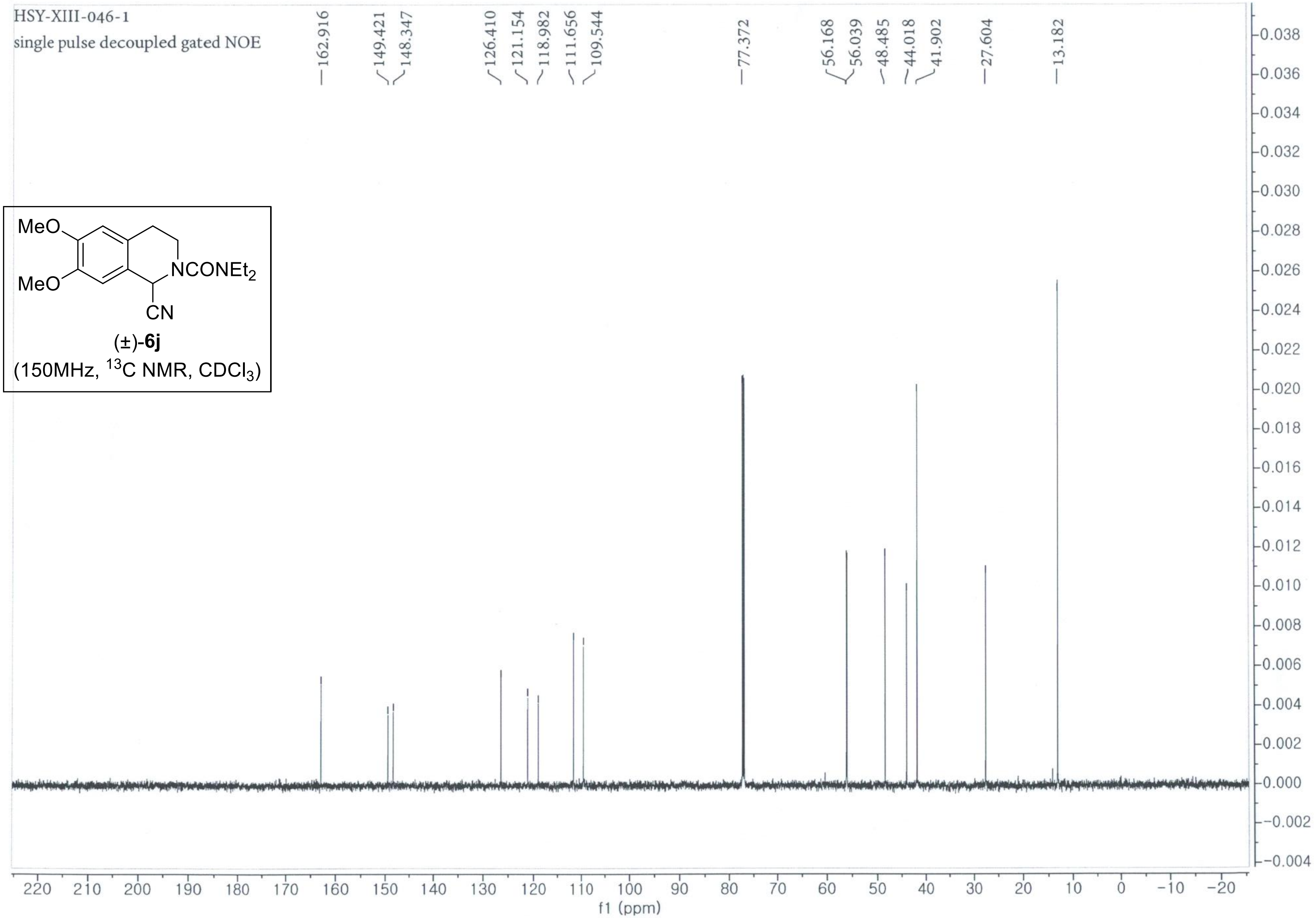
HSY-XIII-046-1
single pulse decoupled gated NOE
162.916
149.421
148.347
126.410
121.154
118.982
111.656
109.544
77.372
56.168
56.039
48.485
44.018
41.902
27.604
13.182
MeO
MeO
NCONEt2
CN
(±)-6j
(150MHz, 13C NMR, CDCl3)
f1 (ppm)

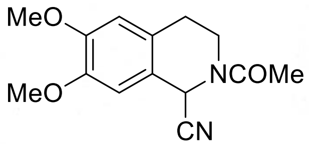

(±)-**6k**

(400MHz, ^{1}H NMR, $CDCl_3$)

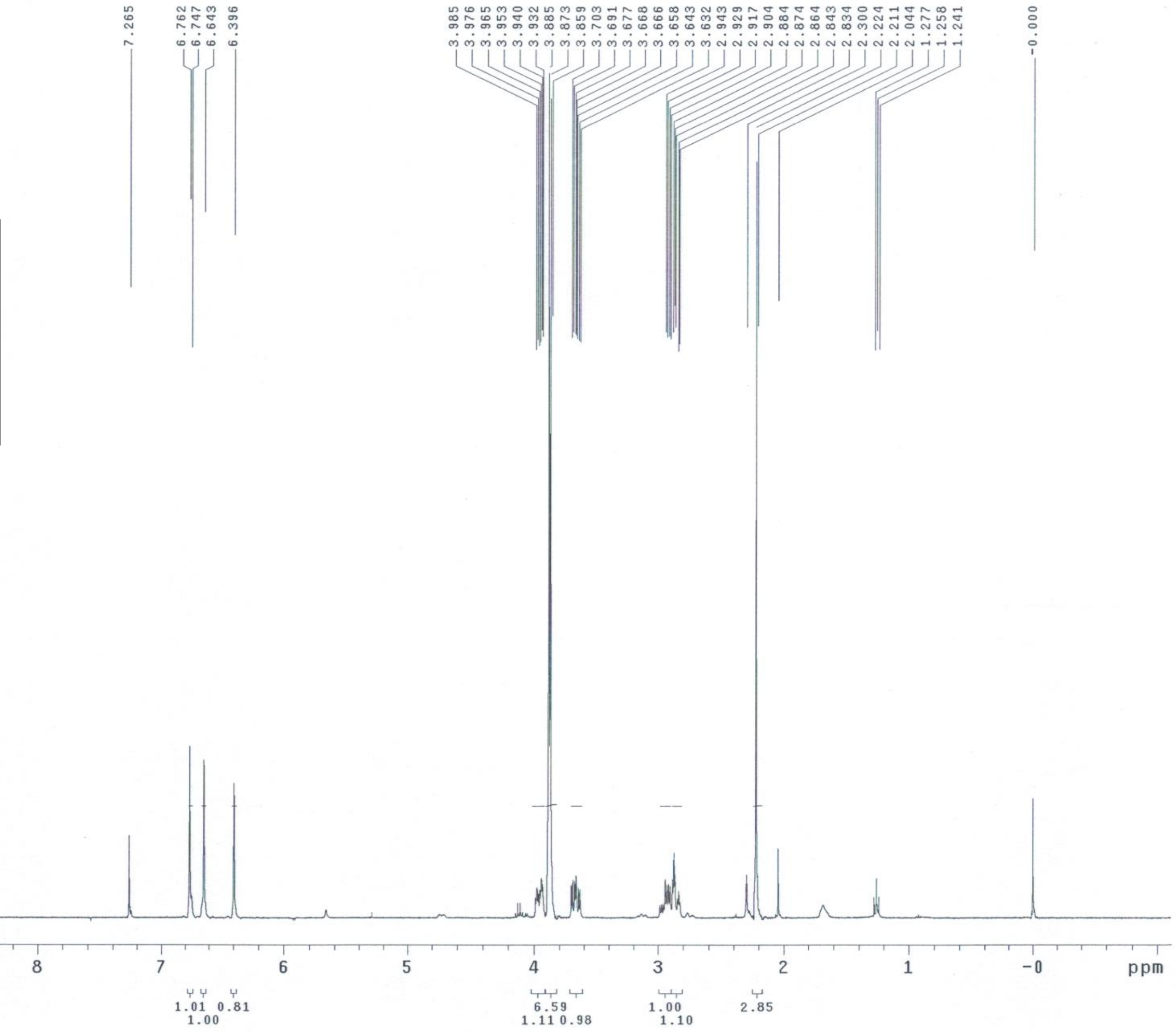

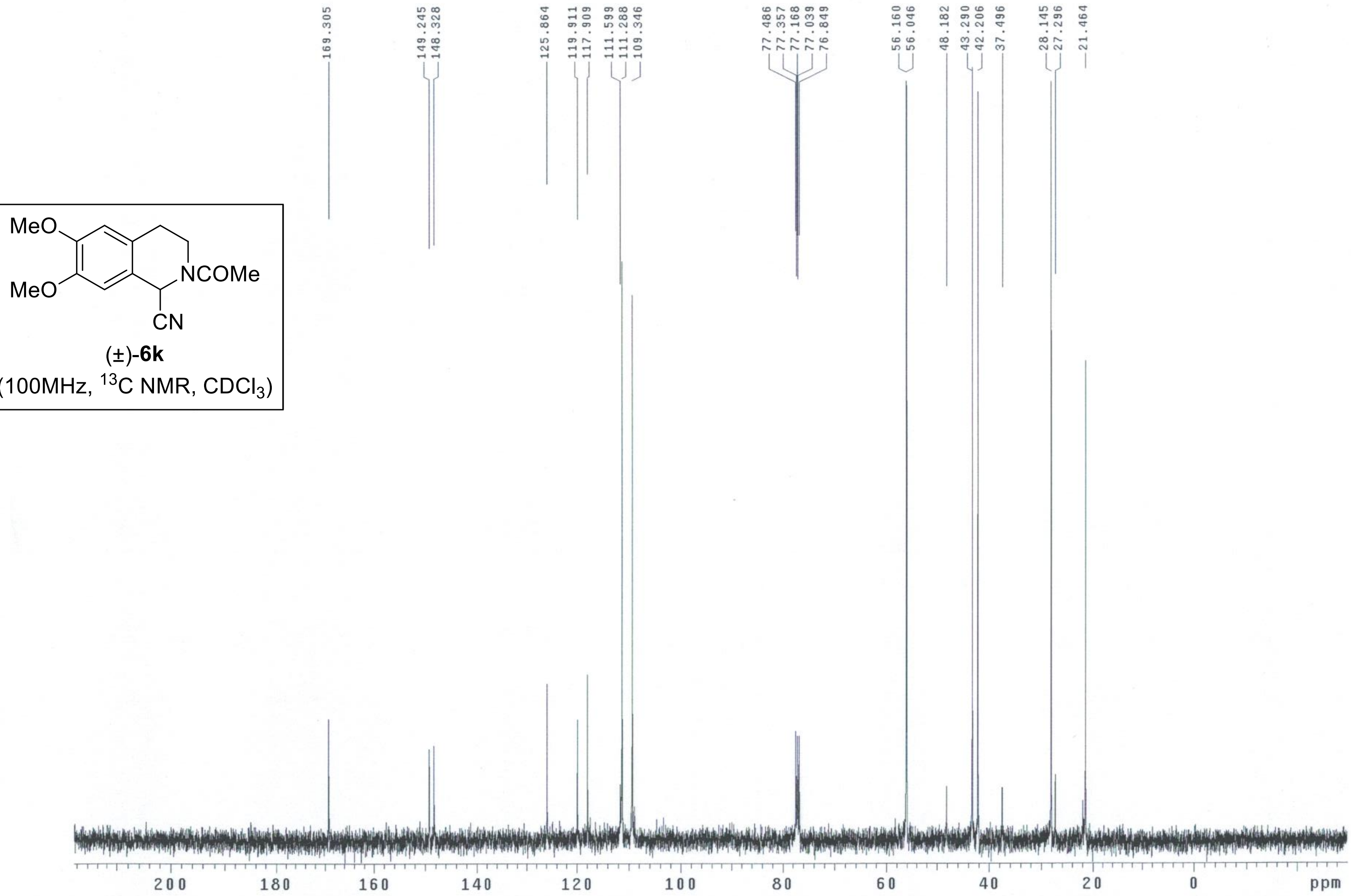

(±)-**6k**
(100MHz, ^{13}C NMR, $CDCl_3$)

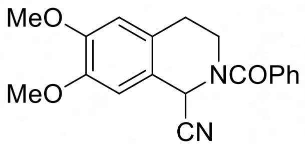

(±)-**6l**

(400MHz, ^{1}H NMR, $CDCl_3$)

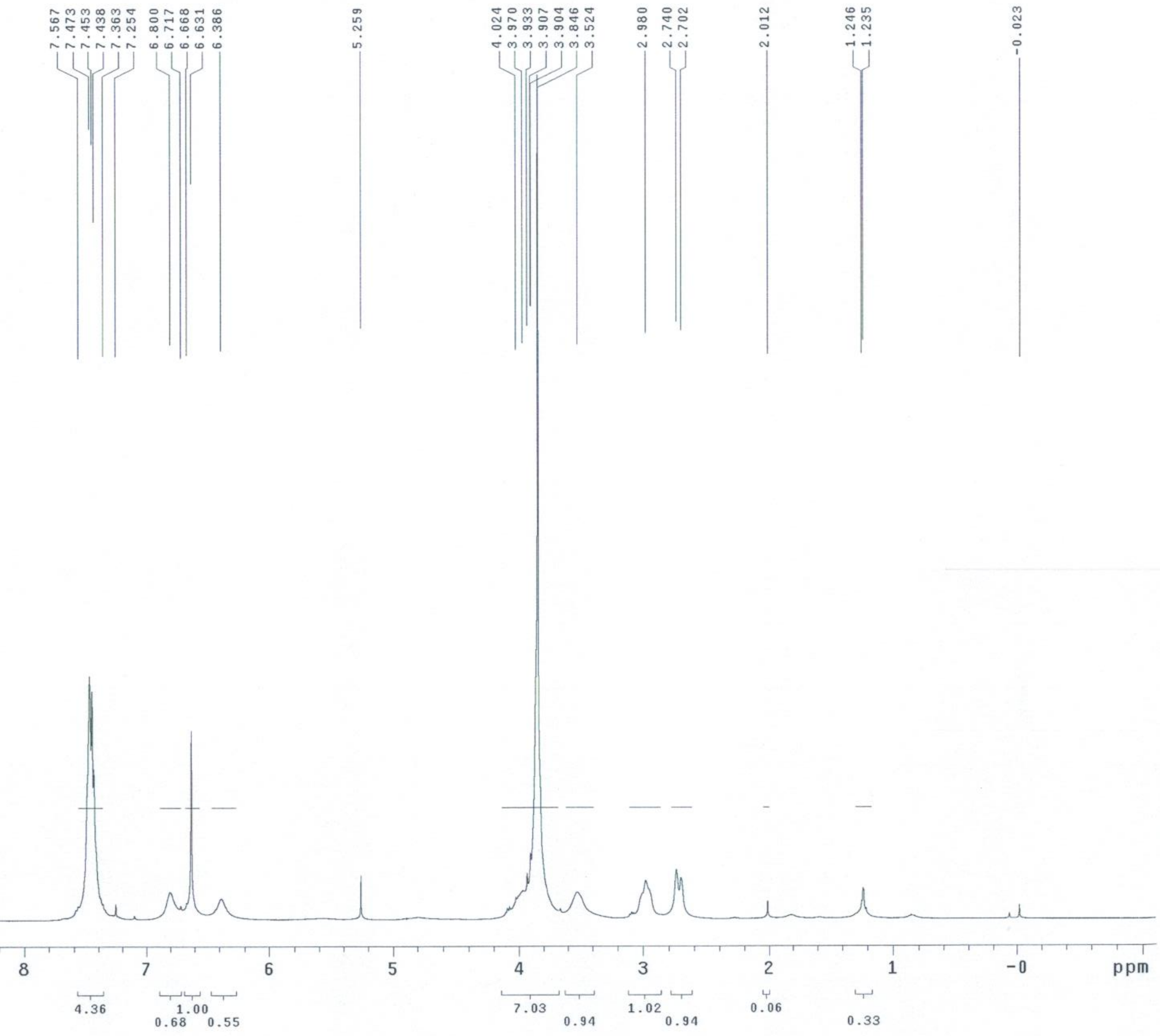

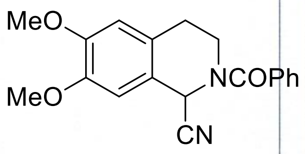

(±)-**6l**
(100MHz, ^{13}C NMR, $CDCl_3$)

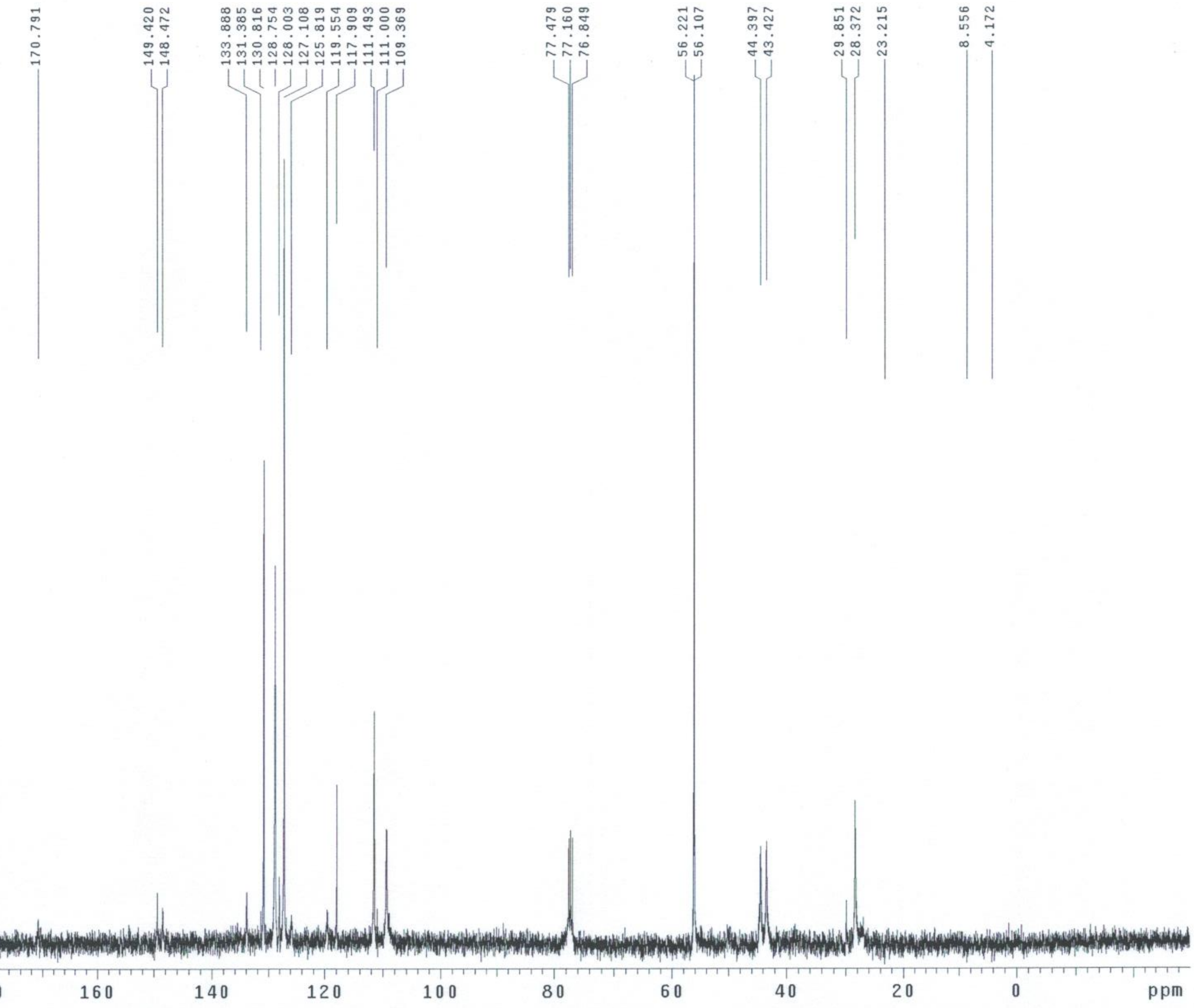

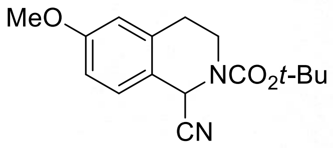

(±)-**6m**

(400MHz, ^{1}H NMR, $CDCl_3$)

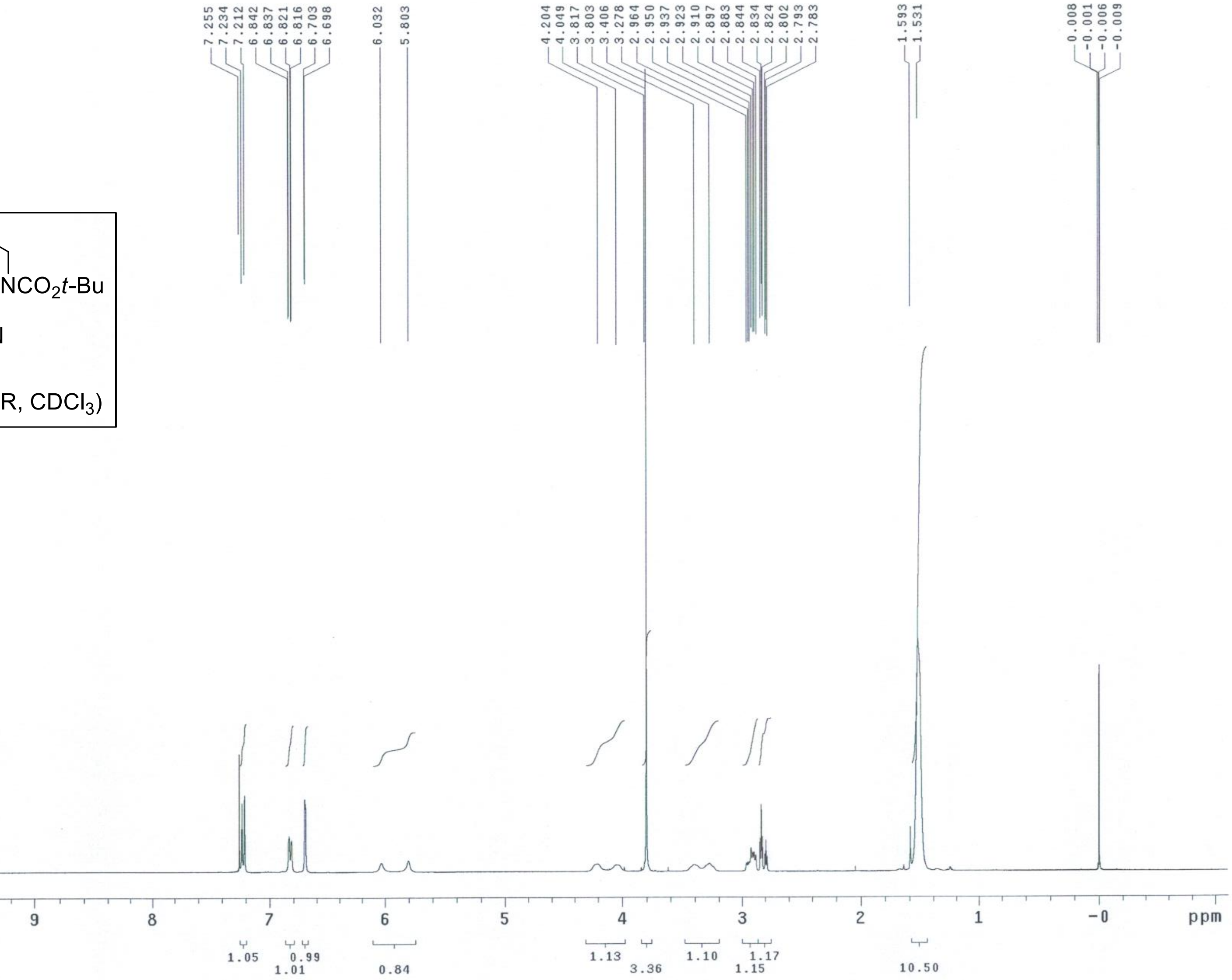

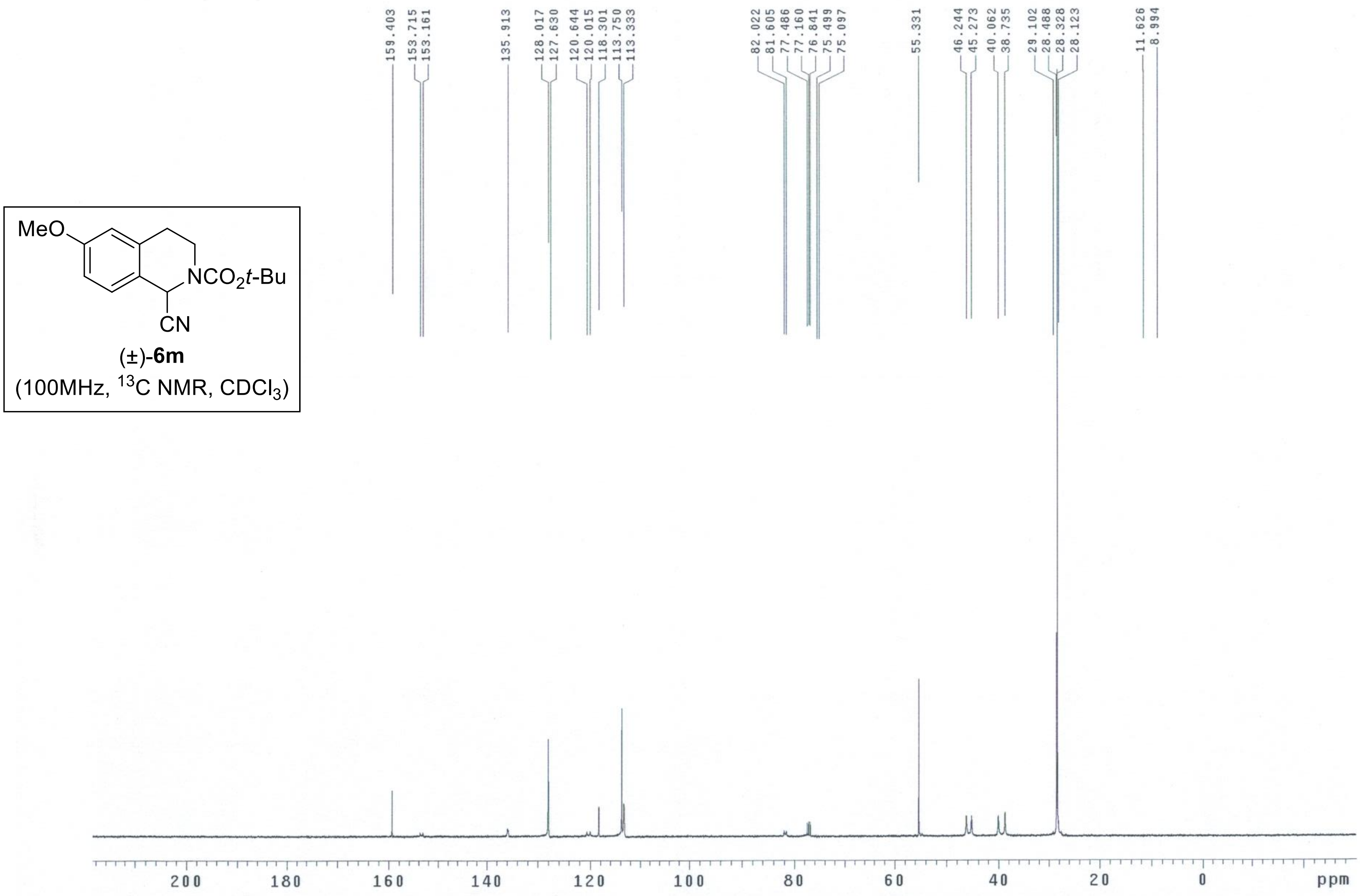

(±)-**6m**

(100MHz, ^{13}C NMR, $CDCl_3$)

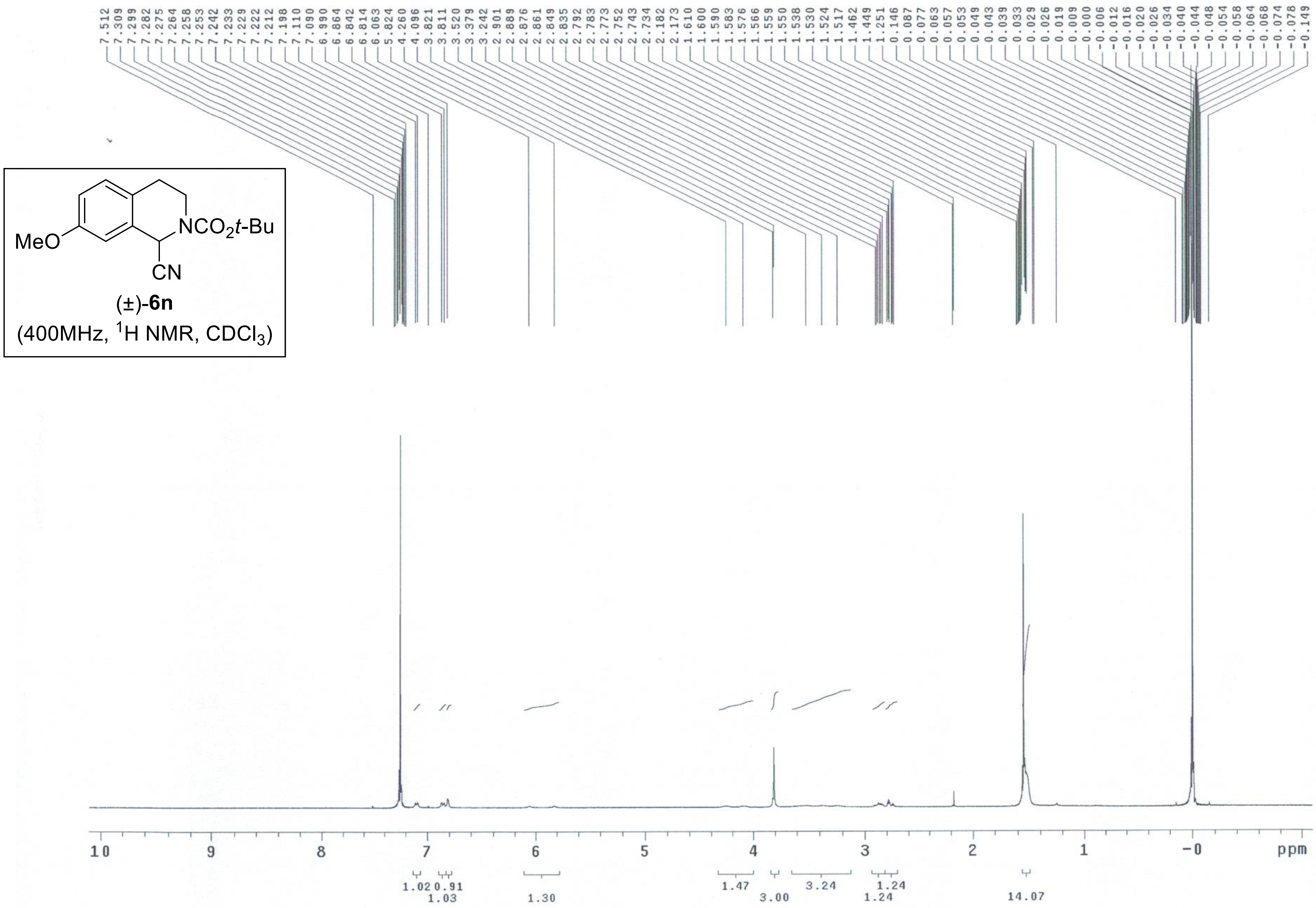
MeO
NCO2t-Bu
CN
(±)-6n
(400MHz, 1H NMR, CDCl3)
ppm

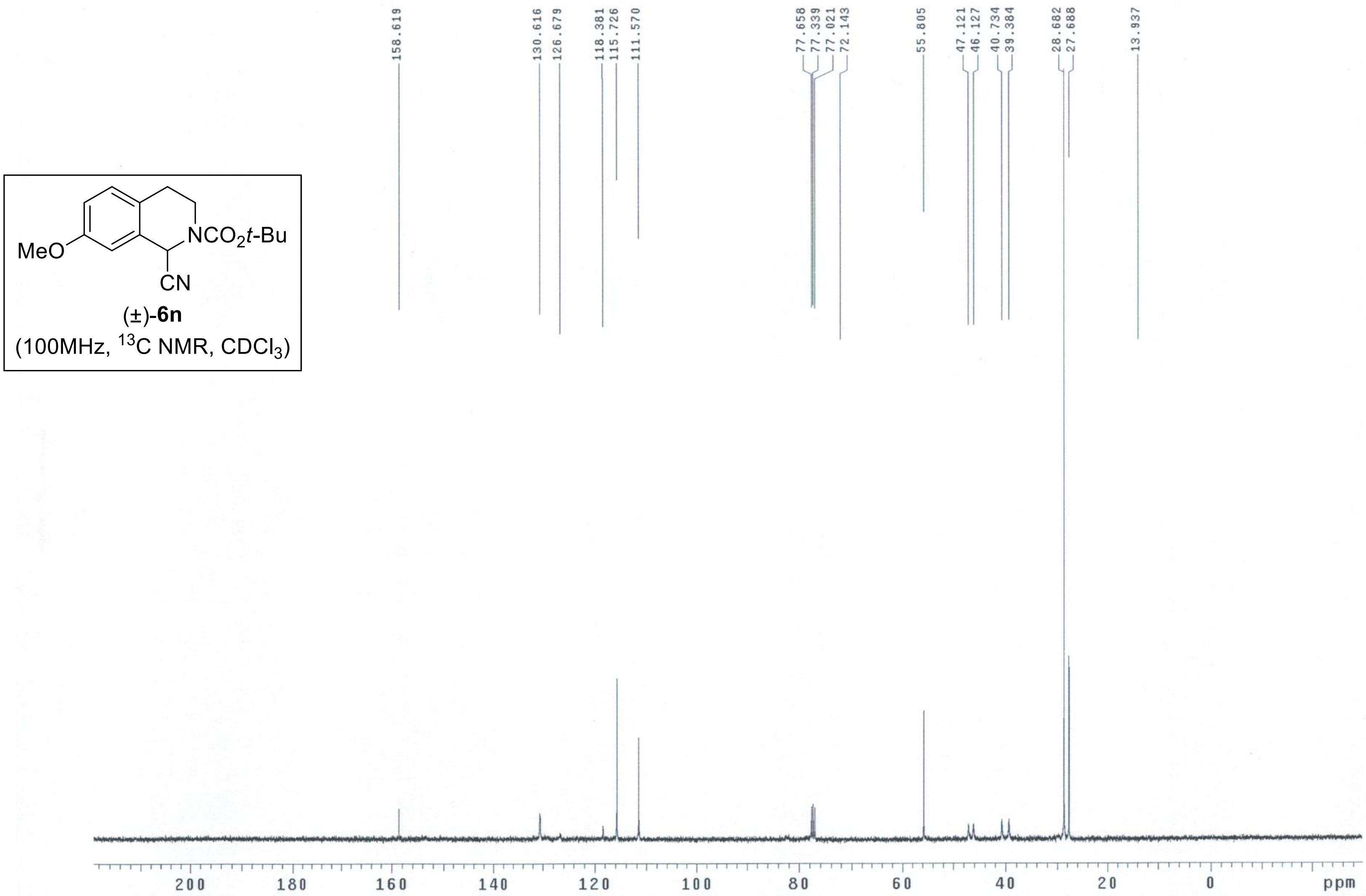

(±)-**6n**
(100MHz, ^{13}C NMR, $CDCl_3$)

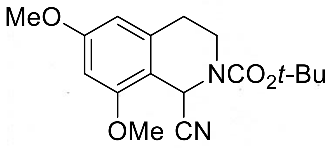

(±)-**6o**

(400MHz, ^{1}H NMR, $CDCl_3$)

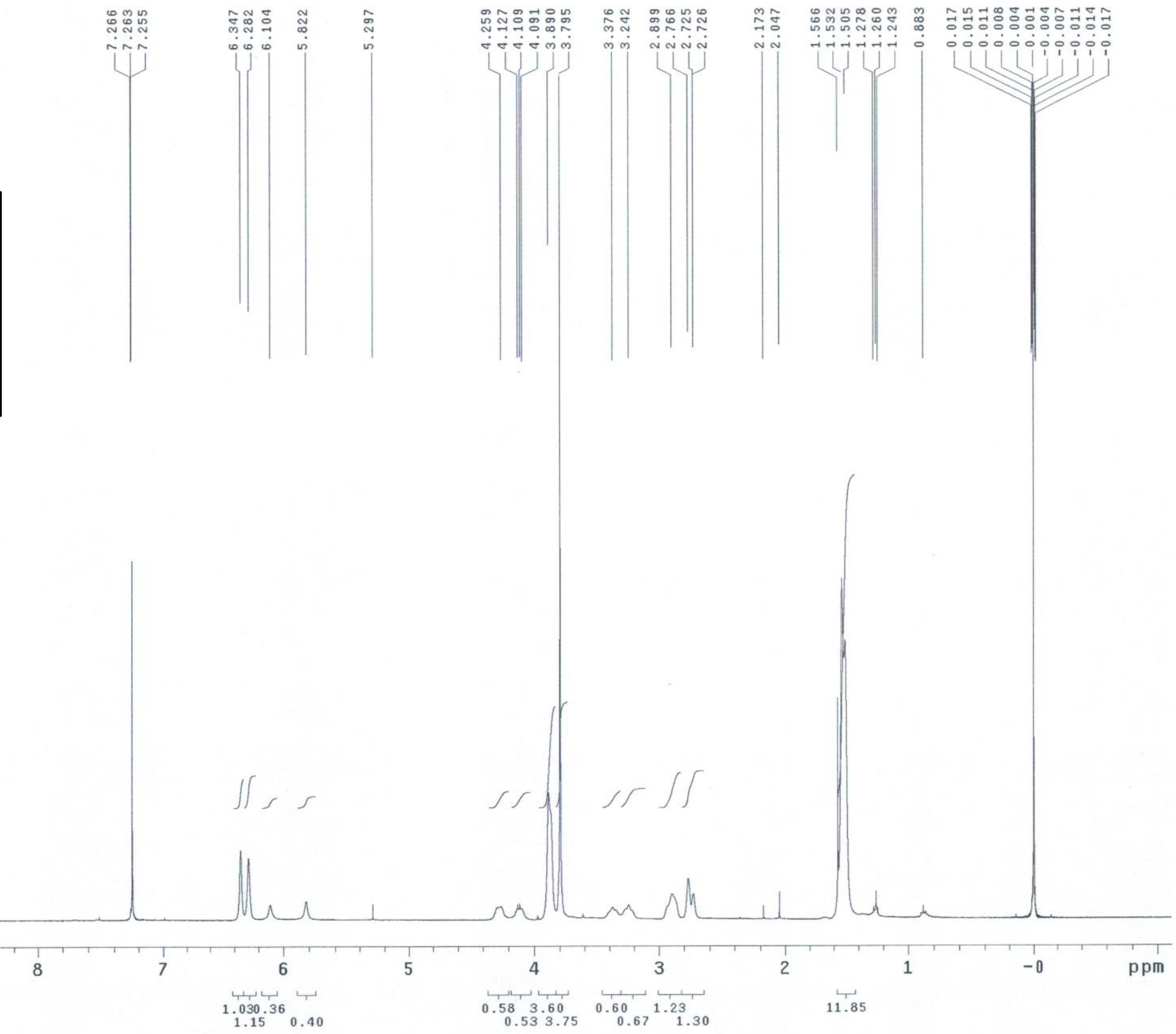

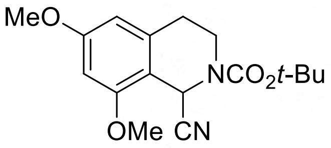

(±)-**6o**

(100MHz, ^{13}C NMR, $CDCl_3$)

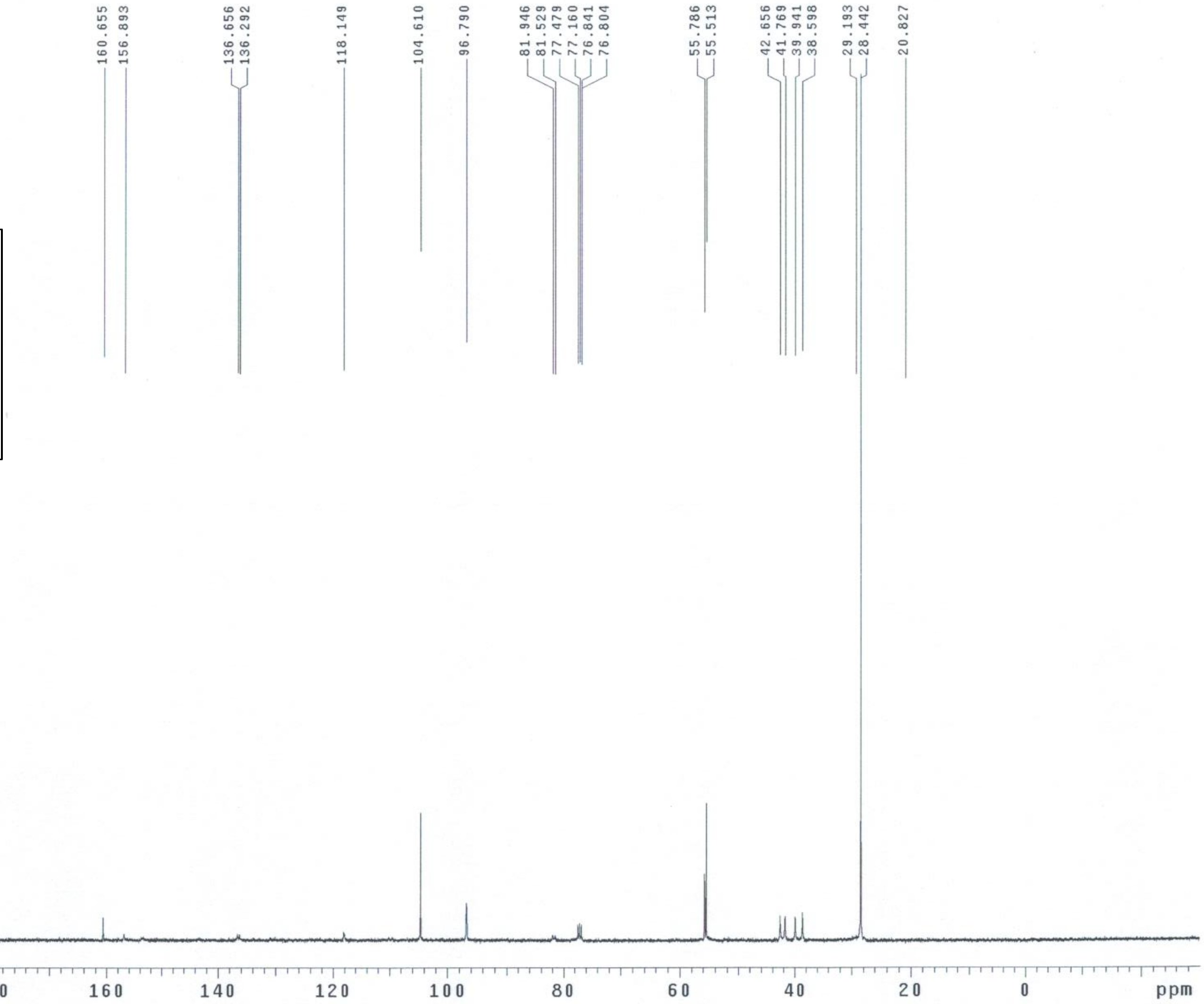

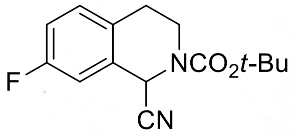

(±)-**6p**

(400MHz, ^{1}H NMR, $CDCl_3$)

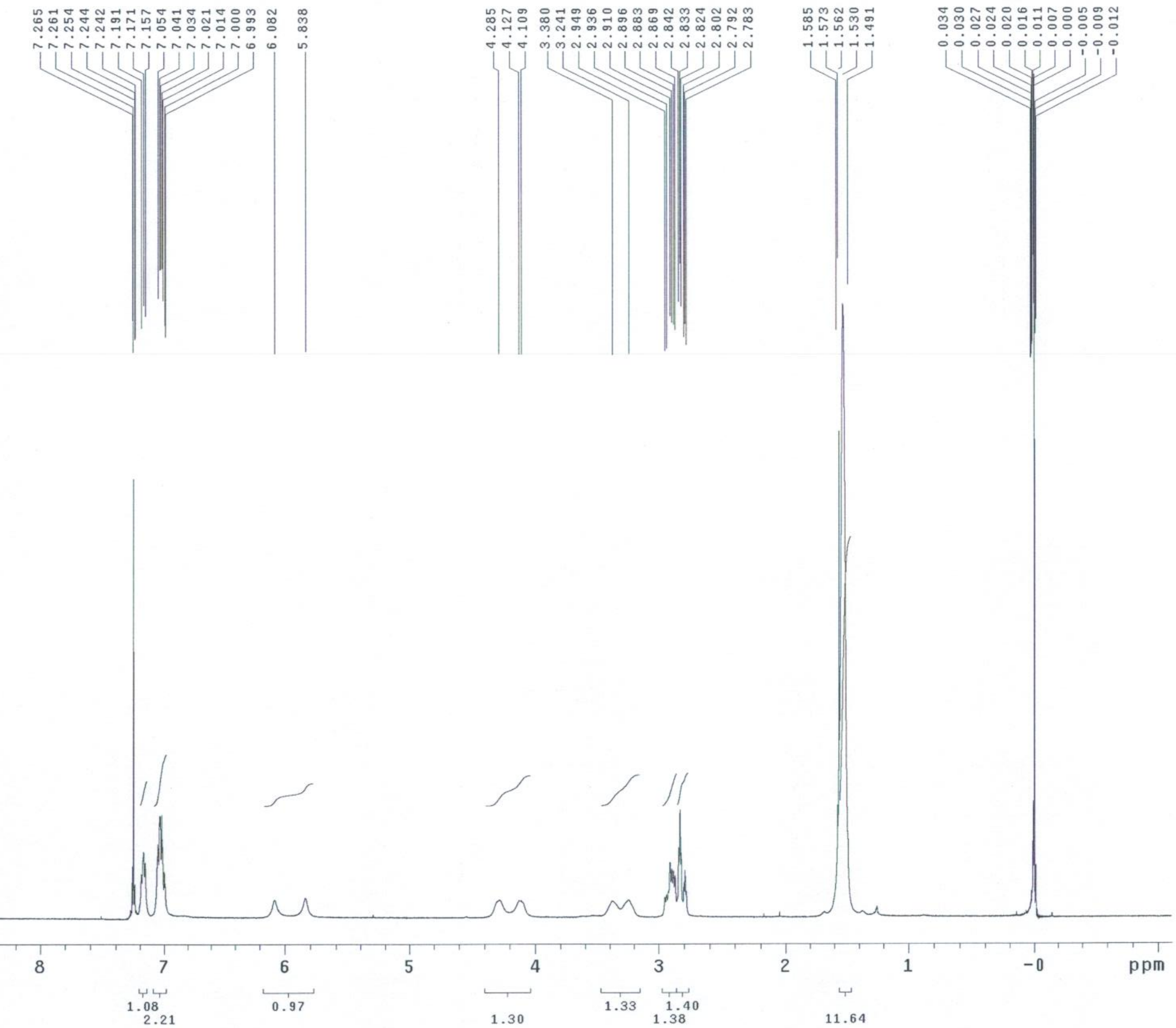

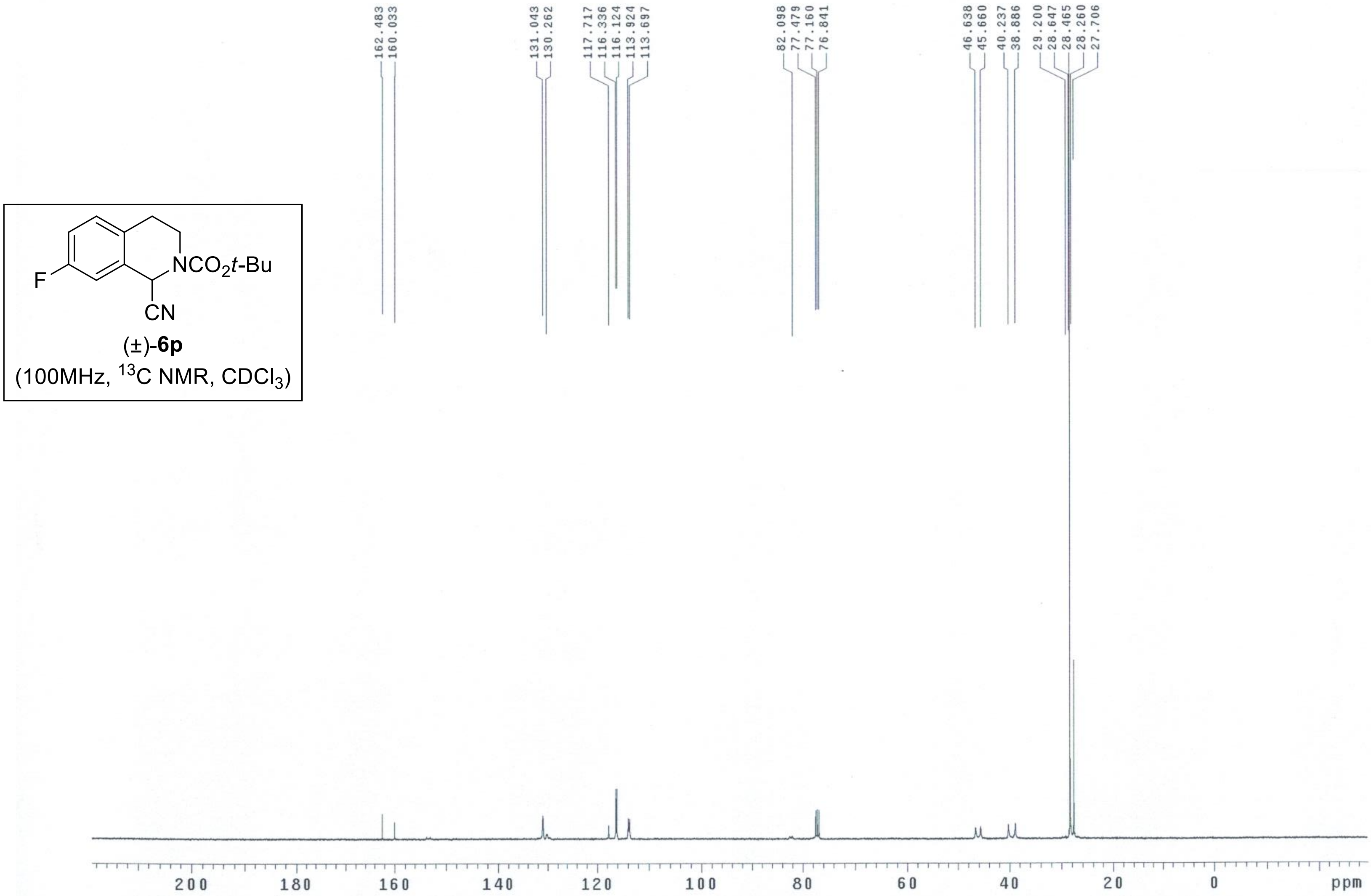

(±)-**6p**

(100MHz, ^{13}C NMR, $CDCl_3$)

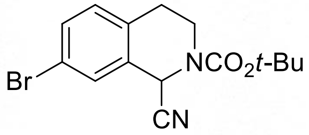

(±)-**6q**

(400MHz, ^{1}H NMR, $CDCl_3$)

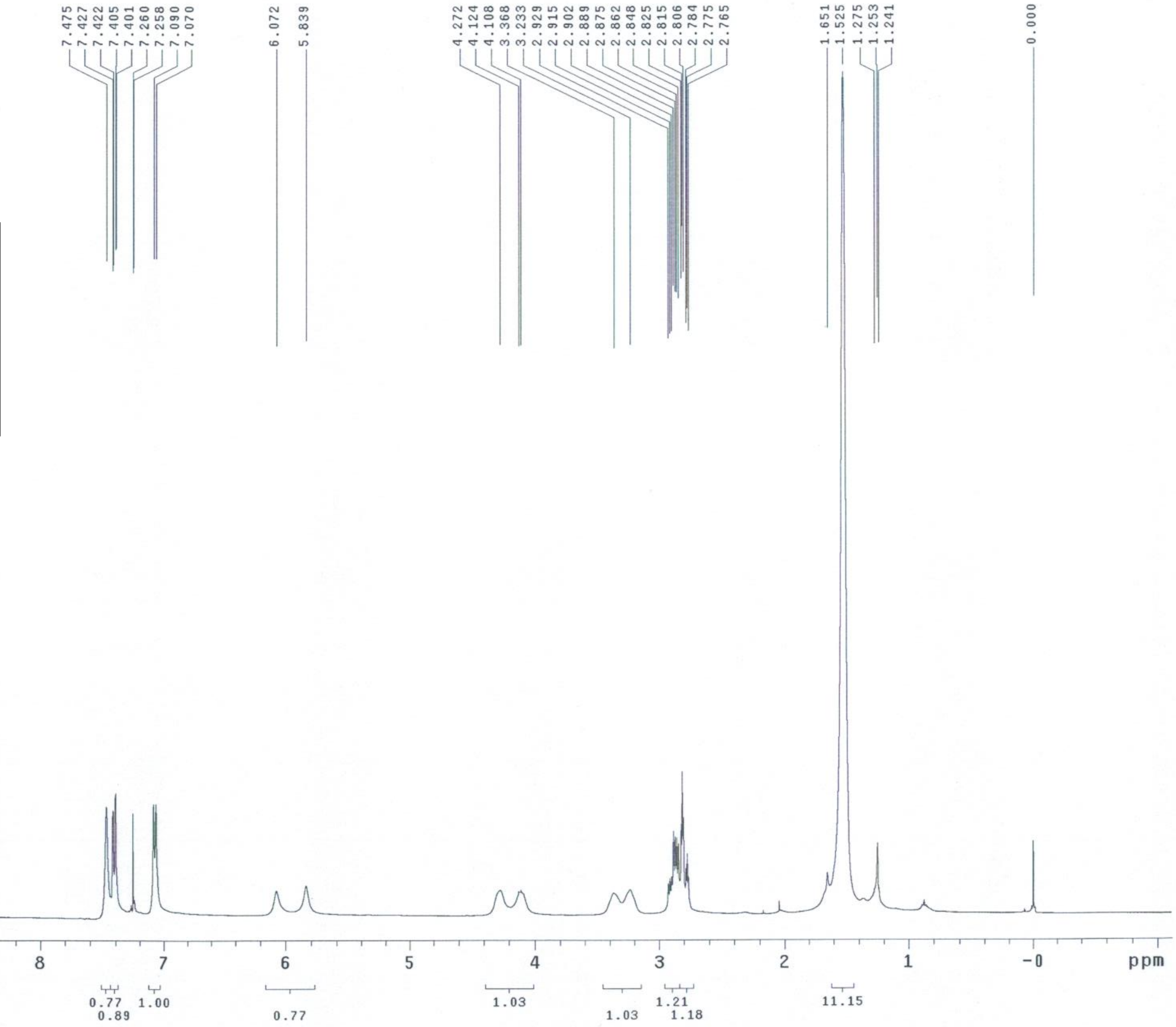

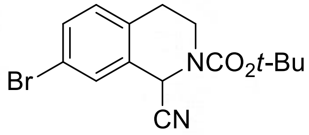

(±)-**6q**
(100MHz, ^{13}C NMR, $CDCl_3$)

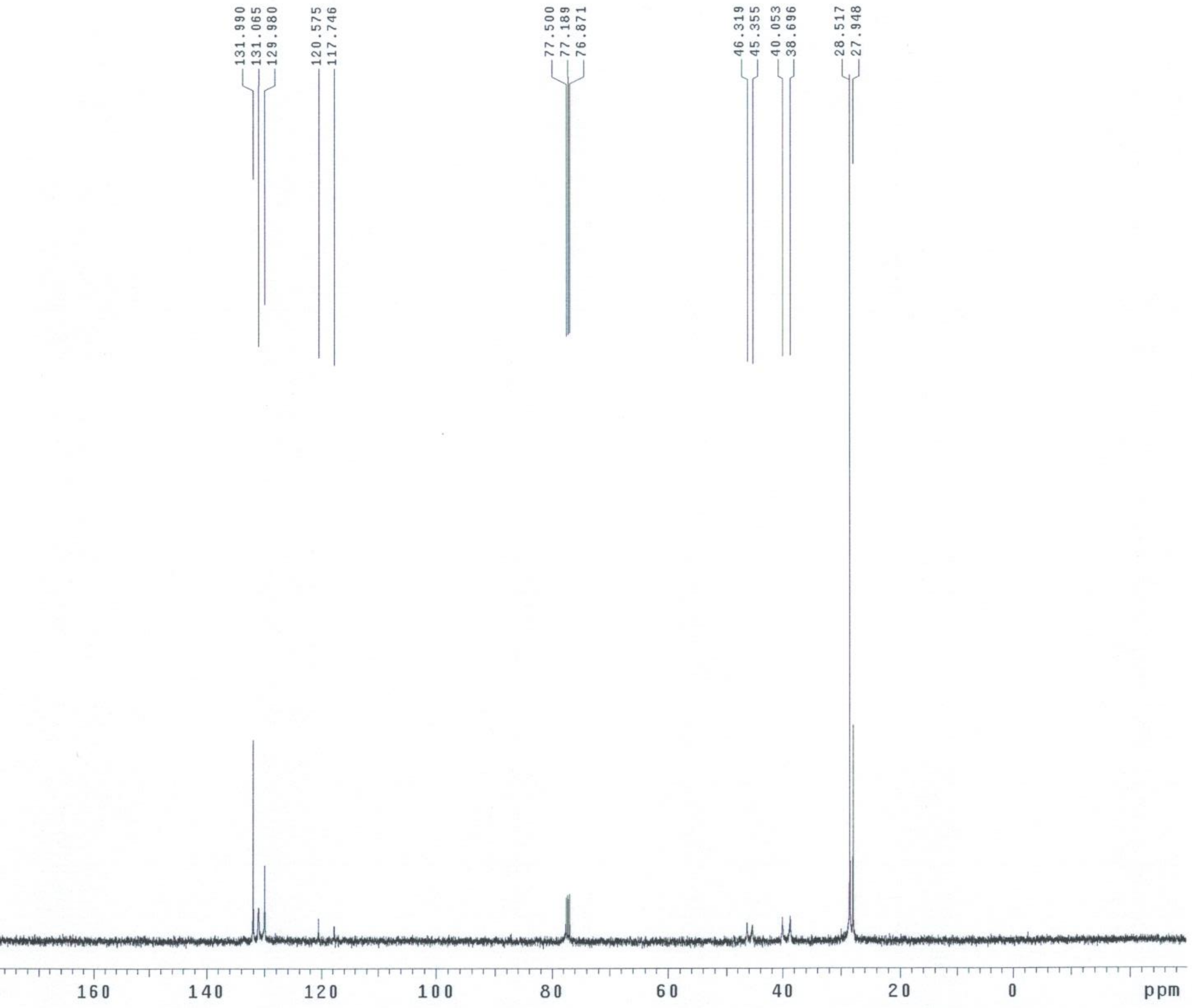

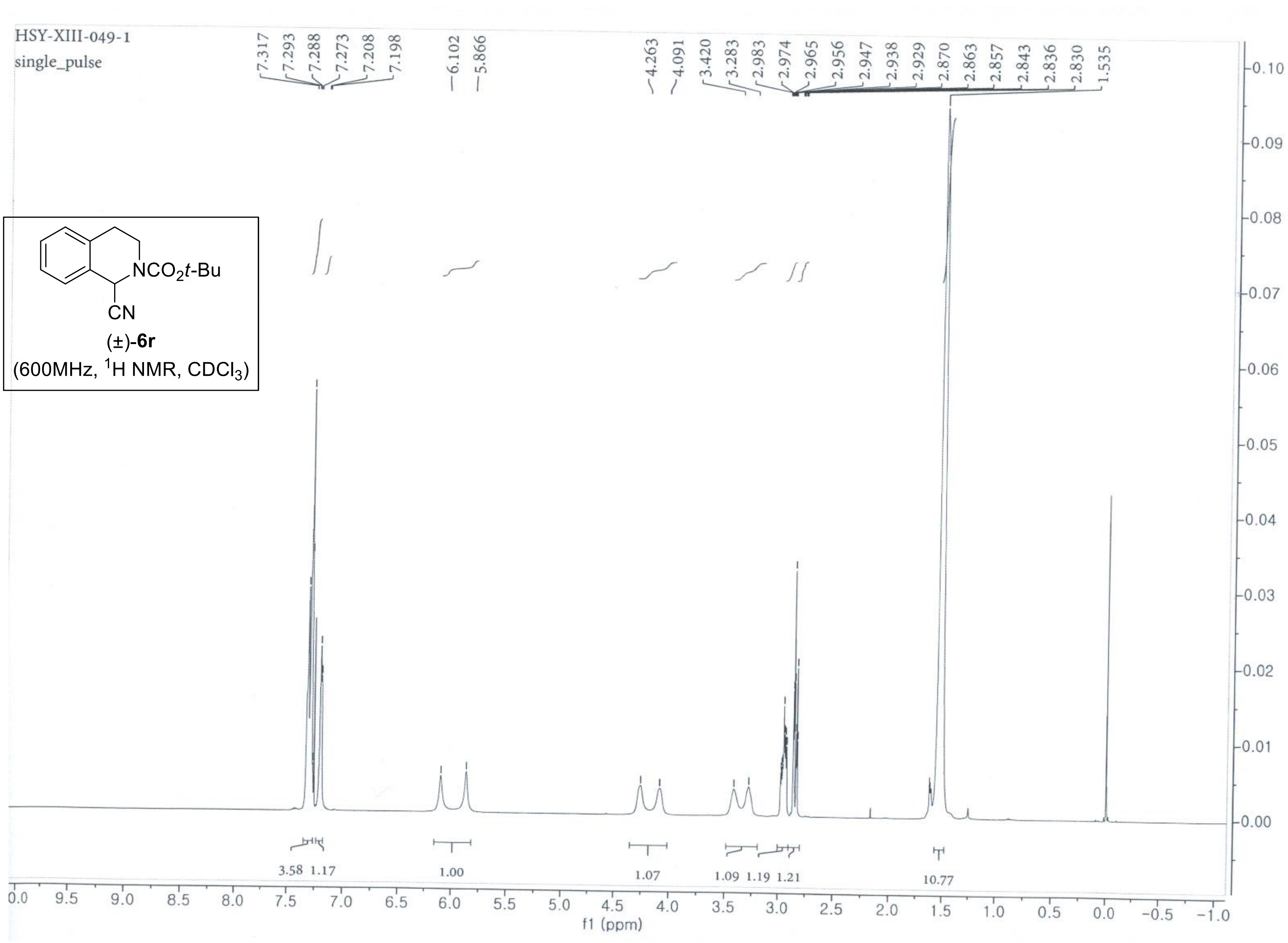
HSY-XIII-049-1
single_pulse
7.317
7.293
7.288
7.273
7.208
7.198
6.102
5.866
4.263
4.091
3.420
3.283
2.983
2.974
2.965
2.956
2.947
2.938
2.929
2.870
2.863
2.857
2.843
2.836
2.830
1.535
NCO2t-Bu
CN
(±)-6r
(600MHz, 1H NMR, CDCl3)
3.58 1.17
1.00
1.07
1.09 1.19 1.21
10.77
f1 (ppm)

HSY-XIII-049-1
single pulse decoupled gated NOE

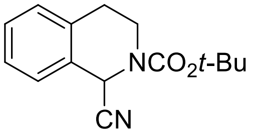

(±)-**6r**
(150MHz, ^{13}C NMR, $CDCl_3$)

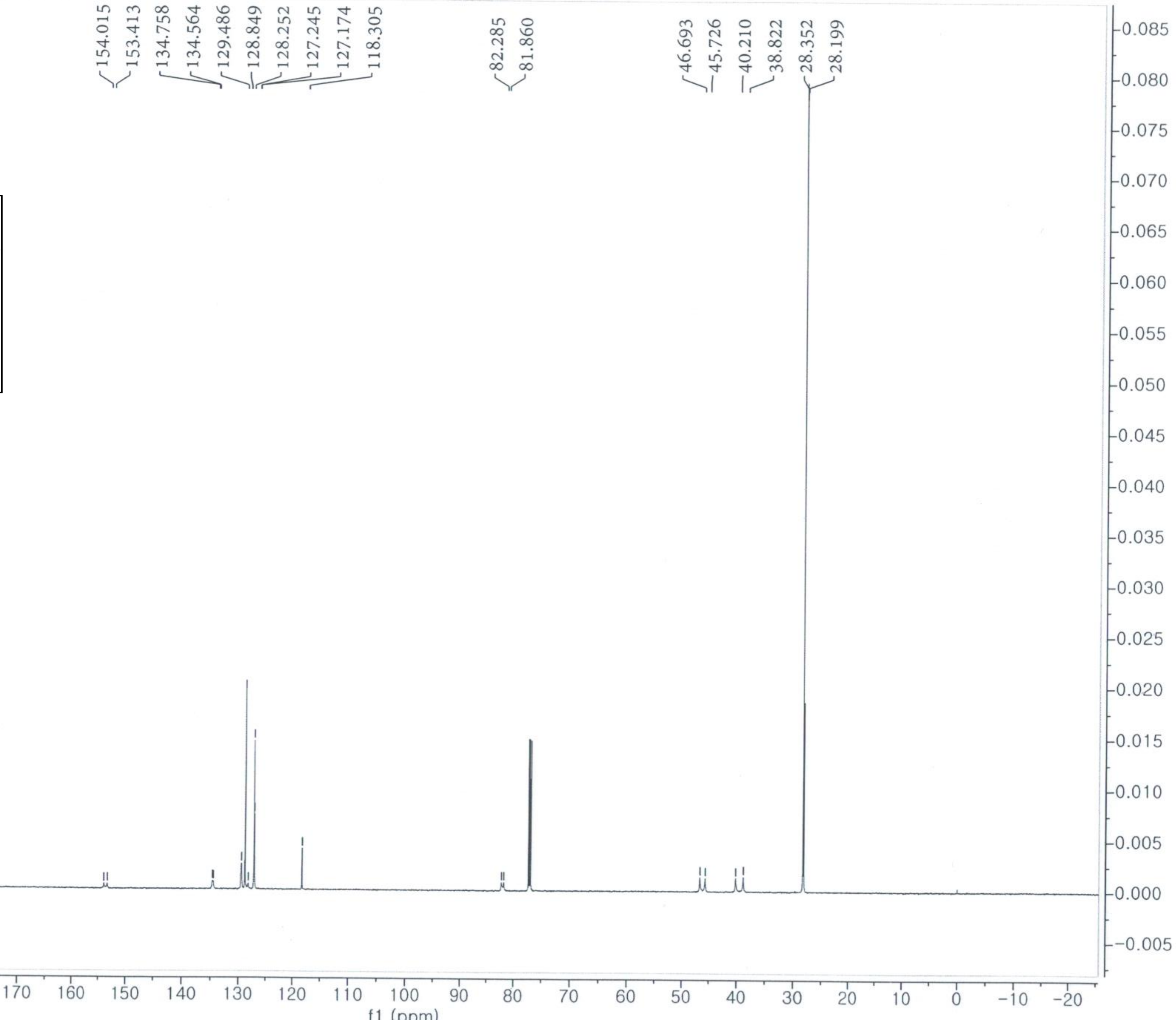

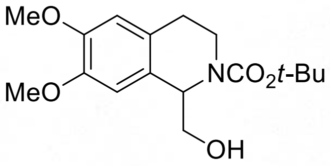

(±)-**8**

(400MHz, ^1H NMR, $CDCl_3$)

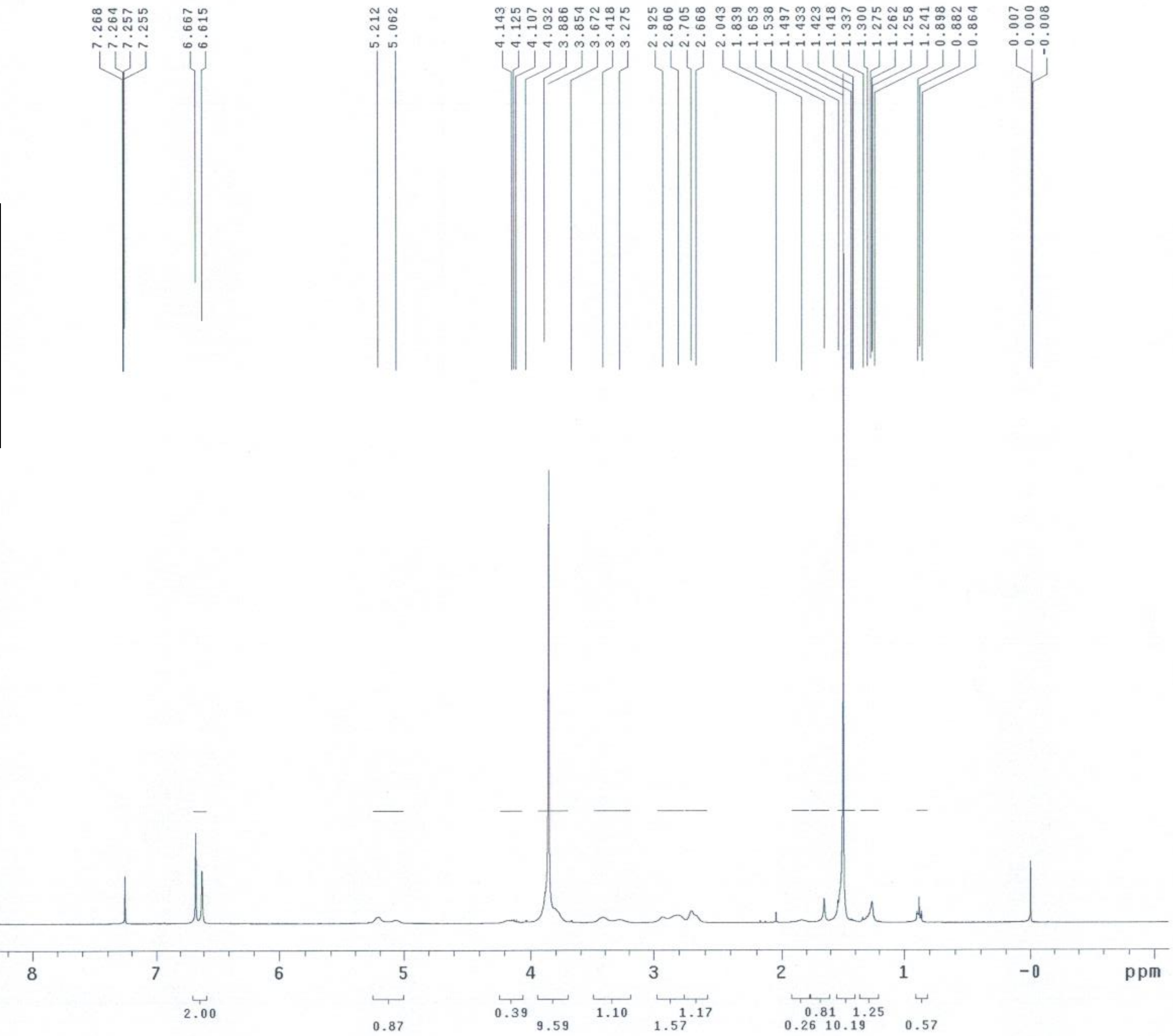

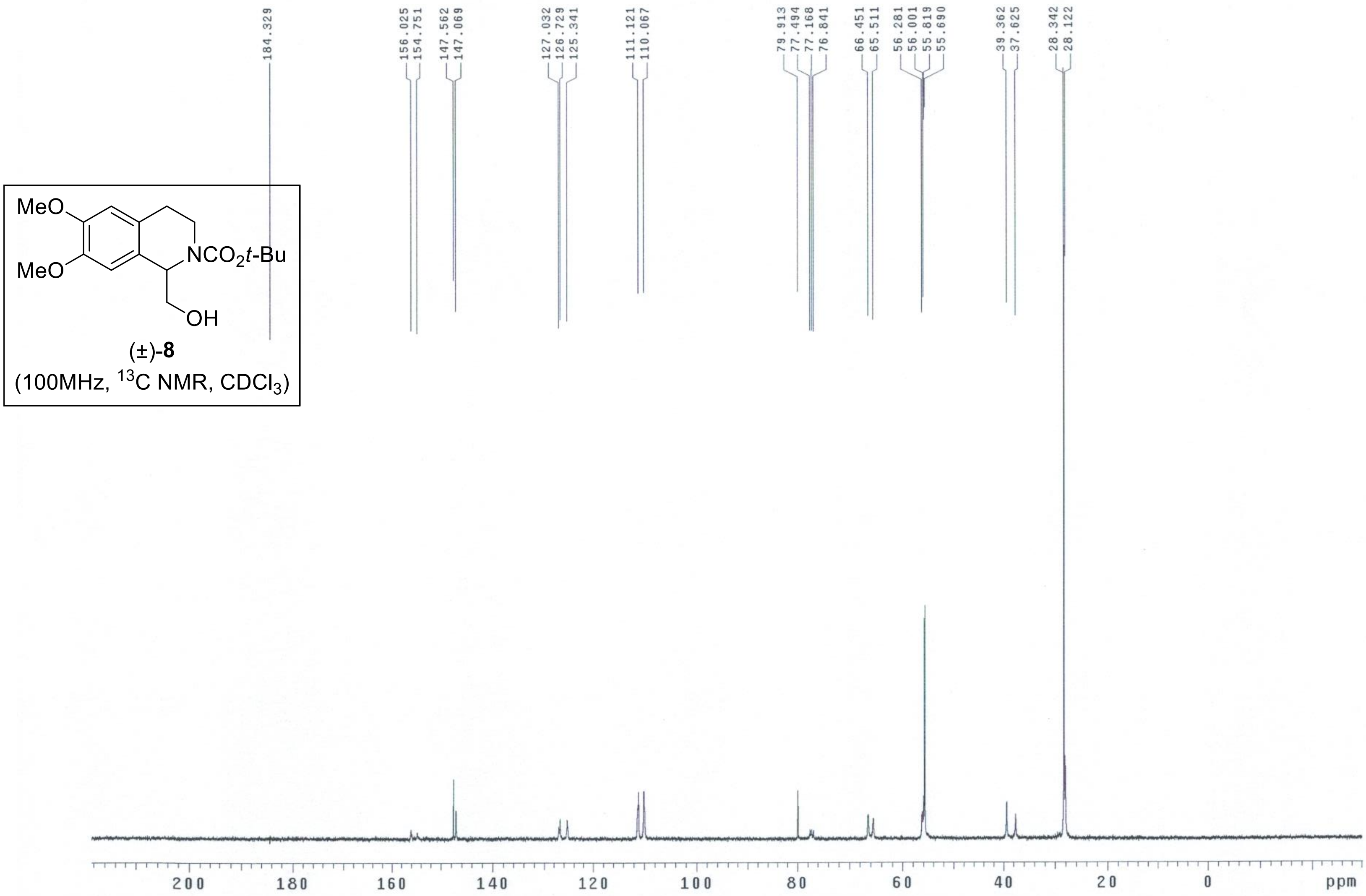

(±)-**8**

(100MHz, ^{13}C NMR, $CDCl_3$)
